# HPCO—A Phosphorus‐Containing Analogue of Isocyanic Acid

**DOI:** 10.1002/anie.201700368

**Published:** 2017-03-02

**Authors:** Alexander Hinz, René Labbow, Chris Rennick, Axel Schulz, Jose M. Goicoechea

**Affiliations:** ^1^ Department of ChemistryUniversity of OxfordChemistry Research Laboratory12 Mansfield RoadOxfordOX1 3TAUK; ^2^ Institut für ChemieUniversität RostockAlbert-Einstein-Strasse 3a18059RostockGermany; ^3^ National Physics LaboratoryHampton RoadTeddington, MiddlesexTW11 0LWUK

**Keywords:** 2-phosphaethynolate, gas-phase IR spectroscopy, isocyanic acid, NMR spectroscopy, phosphorus

## Abstract

We describe the isolation and spectroscopic characterization of the heavier phosphorus‐containing analogue of isocyanic acid (HPCO), and its isotopologue (DPCO). This fundamental small molecule, which has been postulated to exist in interstellar space, has thus far only been observed at low gas phase concentrations or in inert gas matrices. In this report we describe its synthesis, spectroscopic properties, and reactivity in solution.

In 1830, Wöhler and Liebig investigated cyanic acid (HOCN) and its reactivity towards ammonia in an effort to synthesize ammonium cyanate, which was found to afford urea on mild heating.^1^ Given the technological limitations at the time, very little was deduced about the constitution of the species studied. However these discoveries were fundamental in defining the concept of isomerism, for example, the authors discovered that the silver salts of cyanate and fulminate have the same composition. Historic interest in such simple acids has largely focused on fulminic acid (and salts of its conjugate base) in large part due to its explosive properties.^2^, ^3^ The molecular structures of isocyanic (HNCO) and fulminic acid (HCNO), were not determined until 1950 by Herzberg and Reid,^4^ and 1966 by Beck, respectively.^5^ Nowadays, spectroscopic evidence for all four isomeric species isocyanic acid,^6^ cyanic acid,^7^ fulminic acid,^5^ and isofulminic acid is available (Scheme 1).^8^ HNCO,^9^ HOCN,^10^ and HCNO have even been detected in interstellar space.^11^

**Scheme 1:**
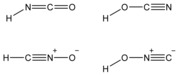
Known isomers in the HNCO system.

In contrast, analytical data for the heavier phosphorus‐containing homologues HPCO are scarce. Several reports by Matveev claim to have synthesized HOCP from phosphaalkyne precursors,^12^, ^13^ however these studies were found to not be reproducible by the group of Becker and others.^14^ Becker and co‐workers suggested the existence of HOCP after protonation of the 2‐phosphaethynolate anion, PCO^−^,^14^, ^15^ but clearly emphasized that this hypothesis was doubtful because the compound could neither be isolated nor further characterized.^16^

Numerous theoretical studies have focused on the different isomers of the HPCO system, predicting HPCO to be 23 kcal mol^−1^ more stable than HOCP, with an activation barrier for interconversion of 70 kcal mol^−1^.^17^, ^18^, ^19^, ^20^, ^21^, ^22^ These computational studies have stimulated further research, indicating that HPCO might be stable in interstellar space. All successful experimental HPCO studies thus far have tried to mimic interstellar conditions, such as low gas‐phase concentration of HPCO or the use of noble gas matrices. Thus, there are two studies of the generation of HPCO by gas discharges of highly dilute PH_3_/CO mixtures (in He or Ar), which allowed the recording of infrared^23^ and microwave spectra of HPCO.^24^, ^25^ These studies have corroborated the presence of HPCO and not the isomeric HOCP. More recently, HPCO has been proposed as an intermediate in the formation of phosphinecarboxamides and phosphinidene‐carbene adducts in the reaction of PCO^−^ with ammonium and imidazolium salts, respectively.^26^, ^27^, ^28^ Without a suitable nucleophile present, protonation of PCO^−^ in solution phase affords an insoluble yellow precipitate and PH_3_, in agreement with previous reports by Becker et al.

HPCO has been proposed to exist in interstellar space as well, but it has not yet been detected there. The only known phosphorus‐containing species in space are HCP,^29^ PC,^30^ PC_2_,^31^ PO,^32^ and PN.^33^, ^34^ However, it is not necessary to look for HPCO in space, or mimic interstellar conditions. As we show in this study, it is possible to generate metastable solutions of this species with standard Schlenk line techniques.

The method employed has been utilized repeatedly in the past to generate HN_3_ since Günther, Meyer, and Müller‐Skjøld introduced it in 1935.^35^ More recently, improved versions have been used for the generation of HN_3_,^36^ HNCO,^6^ and HNSO.^37^ A solid mixture of stearic acid and [Na(dioxane)_*x*_]PCO (*x*=1) was heated in vacuo (Scheme 2),^38^ thereby releasing a gas which was condensed into an NMR tube containing a solvent (toluene, dichloromethane or THF) in a bath of liquid nitrogen. The condensate was yellow and upon warming to −78 °C dissolved in the solvent. The yellow solution was subjected to low temperature NMR experiments. A representative spectrum is shown in Figure 1. The resonance at −211.6 ppm can be attributed to diphosphine (P_2_H_4_). On prolonged standing a resonance at −240.6 ppm also grows in which corresponds to phosphine (PH_3_). In addition to these trace impurities, there is a more upfield shifted resonance corresponding to HP^12^CO at −316.7 ppm, which, upon proton coupling transforms to a doublet with ^1^ *J*_P‐H_ =188 Hz (Table 1). The resonance shifts slightly in dependence on solvent polarity (CD_2_Cl_2_: −312.8 (*189*), [D_8_]THF: −316.7 (*192)* ppm; see the Supporting Information for additional spectra). The ^13^C‐coupled ^31^P NMR resonance for HP^13^CO was also detected at −316.9 ppm in [D_8_]toluene (^1^ *J*_P‐H_ =188, ^1^ *J*_P‐C_ =102 Hz).

**Figure 1:**
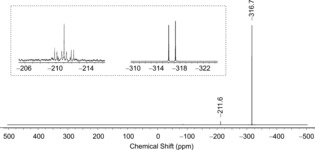
^31^P{^1^H} NMR spectrum of the condensate from the reaction of [Na(dioxane)_*x*_]PCO (*x*=1) and stearic acid ([D_8_]toluene, 223 K). Inset: ^31^P NMR multiplets.

**Scheme 2:**
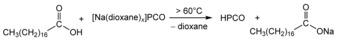
Generation of HPCO.

**Table 1:** NMR data for HPCO. Chemical shift values in ppm, coupling constants in Hz

nucleus	[D_8_]toluene	CD_2_Cl_2_	[D_8_]THF^[a]^	calc.^[b]^
*δ*(^1^H) (^1^ *J*_H‐P_)	0.25 (188)	1.09 (189)	1.32 (192)	1.85
*δ*(^13^C) (^1^ *J*_C‐P_)	201.4 (102)	201.5 (98)	202.2 (101)	207
*δ*(^31^P) (^1^ *J*_P‐H_)	−316.7 (188)	−312.8 (189)	−316.7 (192)	−309^[c]^

[a] Recorded at 193 K. [b] See Ref. 39. [c] Referenced to δ(PH_3_) = −240 ppm.

This finding is in good agreement with computations which predict a chemical shift of −309 ppm.^39^, ^40^ There is a corresponding doublet resonance in the ^1^H NMR spectrum at 0.25 ppm in [D_8_]toluene (CD_2_Cl_2_: 1.09, [D_8_]THF: 1.32; calc. 1.85 ppm, Figure 2), which collapses to a singlet upon ^31^P decoupling. The ^13^C NMR resonance of HPCO was observed as a doublet of doublets at 201.4 ppm in [D_8_]toluene (CD_2_Cl_2_ 201.5, [D_8_]THF 202.2; calc. 207 ppm), showing ^1^ *J*_C‐P_ =102 Hz as well as coupling to the proton with a ^2^ *J*_C‐H_ of 11 Hz.

**Figure 2:**
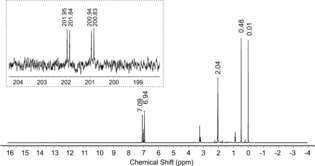
^1^H NMR spectrum of HPCO ([D_8_]toluene, 223 K). Inset: ^13^C NMR spectrum.

The observed spectra for HPCO deviate significantly from the known anion PCO^−^, for which δ(^31^P)=−396.8 and δ(^13^C)=170.3 ppm (^1^ *J*_C‐P_ =62 Hz) was recorded,^41^ but are well within the broad range of shifts observed for known phosphaketenes [for example, (DippNCH_2_)_2_P−PCO: δ(^31^P)=−232.6 ppm (d, ^1^ *J*_P‐P_ =252.5 Hz);^42^ [E]−PCO (E=Re, Co, Cu, Au, Si‐Ge: −200.2 to −397.5 ppm],^43^, ^44^, ^45^, ^46^, ^47^, ^48^ and related species ([U]/[Th]−OCP: −334, −285 ppm).^49^ The chemical shifts also bear resemblance to known R_2_CPH species for which a considerable dependence on solvent polarity was also observed for the δ(^1^H) shift [R_2_C=^Dipp^NHC: δ(^31^P)=−136.7, δ(^1^H)=1.92, ^1^ *J*_P‐H_ =164 Hz; R_2_C=^Ar*^NHC: δ(^31^P)=−134.5, δ(^1^H)=2.10, ^1^ *J*_P‐H_ =172 Hz; R_2_C=various NHC from Ref. 51: δ(^31^P)=−136.7 to −149.3, δ(^1^H)=1.68–2.63, ^1^ *J*_P‐H_ =164–171 Hz; R_2_C=(Me_2_N)_2_C: δ(^31^P)=−62.6, δ(^1^H)=3.10, ^1^ *J*_P‐H_ =159 Hz; all chemical shift values in ppm].^28^, ^50^, ^51^, ^52^

By employing [D_1_]‐stearic acid, it is also possible to generate DPCO, for which the following data were recorded in toluene at 223 K: δ(^2^H)=0.29 (^1^ *J*_D‐P_ =30 Hz; Figure 3), δ(^13^C)=202.1 (^1^ *J*_C‐P_ =101, ^2^ *J*_C‐D_ =2 Hz), δ(^31^P)=−317.7 (^1^ *J*_P‐D_ =30 Hz) ppm. Furthermore, the ^13^C‐coupled ^31^P NMR resonance for ^2^HP^13^CO was found at −317.9 ppm (dd, ^1^ *J*_P‐C_ =30 Hz, ^1^ *J*_P‐D_ =30 Hz).

**Figure 3:**
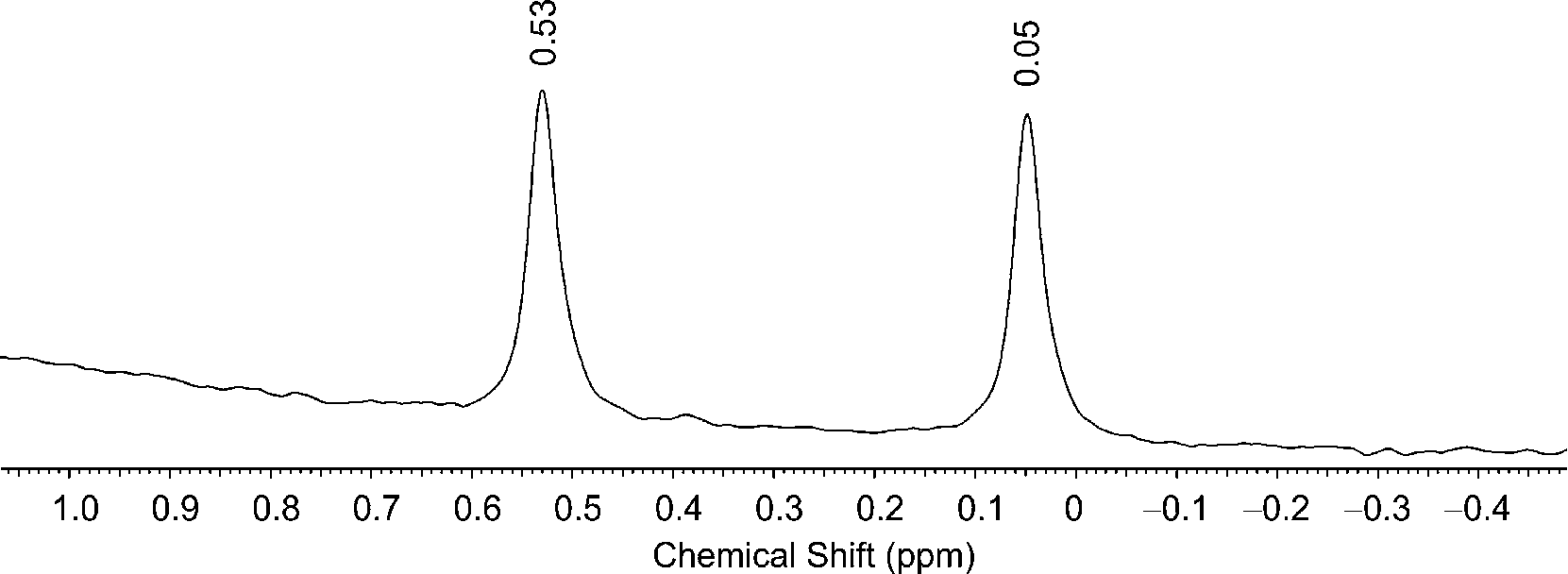
^2^H NMR spectrum of DPCO ([D_8_]toluene, 223 K).

Solutions of HPCO generated by heating mixtures of [Na(dioxane)_*x*_]PCO (*x*=1) and stearic acid are relatively stable in case of dichloromethane or toluene as a solvent, and HPCO persists for more than 8 hours at −50 °C without signs of decomposition. Warmed to ambient temperature, it completely decomposes within 30 minutes. In contrast, in THF solution HPCO decomposes already at low temperature to give various phosphorus‐containing species. On the basis of ^1^H and ^31^P NMR data, several oligomers were identified (Scheme 3). Initially, a dimer of HPCO (**A**) was formed, giving rise to two doublets in the ^31^P{^1^H} NMR spectrum at 305.0 and 75.7 ppm (calc. 367, 101 ppm) with a ^2^ *J*_P‐P_ coupling constant of 17 Hz, as well as a broad resonance at 14.65 and a doublet of doublets at 7.32 ppm (^1^ *J*_P‐H_ =166, ^3^ *J*_P‐H_ =17.0 Hz) in the ^1^H NMR spectrum. Over time, three species with three, and one species with four coupling phosphorus atoms were observed. We have tentatively assigned these species as three trimeric products of HPCO (**B**–**D**) and a tetramer (**E**) on the basis of their ^31^P NMR spectra with the assistance of computational data.

**Scheme 3:**
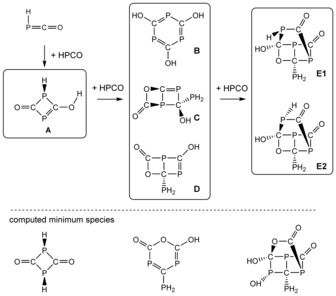
Oligomerization of HPCO in THF at −50°C. Minimum species for the (HPCO)_*n*_ oligomers (*n*=2–4) pictured in dashed squares.

The structures as shown in Scheme 3 were assigned to the following species. Trimer **B** (δ(^31^P)=+130.1, calc. +157 ppm) has previously been observed by the group of Grützmacher, but accessed by a different route.^53^ Trimer **C** (δ(^31^P)=−100.7, 164.0, 289.8; calc. −75, 165, 291 ppm) and **D** (δ(^31^P)=−134.0, 151.7, 298.2, calc. −110, 181, 297 ppm) both bear no PH moieties, but a PH_2_ and an OH group, giving rise to the observed NMR pattern. The formation of tetramer **E** (δ(^31^P)=−92.0, −24.4, 164.6, 299.9; calc. **E1** −100, 8, 205, 297; **E2** −99, −43, 242, 284 ppm) can occur by addition of an HPCO molecule to trimer **D**. Interestingly, none of these oligomers are thermodynamic reaction products (the energetically most favorable isomers are highlighted at the bottom in Scheme 3), but rather intermediates on the pathway of the decomposition reaction.

After warming solutions of HPCO in toluene or dichloromethane to ambient temperature, only phosphine and diphosphine were present in solution. However, in the gas phase, HPCO is stable enough at ambient temperature to allow for recording of gas‐phase IR spectra (Table 2). Heating a mixture of stearic acid and [Na(dioxane)_*x*_]PCO (*x*=1) in an evacuated vessel connected to a gas IR cell allows recording of the gas IR spectrum (HPCO: Figure 4, DPCO: see the Supporting Information). The species do not persist for long under these conditions and after an hour they are no longer observable. The spectra are dominated by a strong characteristic band at 2011 cm^−1^ for HP^12^CO and 2012 cm^−1^ for DP^12^CO which is assigned to the ν_CO_ stretch mode (displacement vectors in the Supporting Information). The corresponding vibrations for HP^13^CO and DP^13^CO were observed at 1963 and 1964 cm^−1^, respectively. These are at higher energy than previously reported for matrix‐isolated HPCO at 32 K (ν_CO_ [cm^−1^]: HP^12^CO 1998, HP^13^CO 1950, DP^12^CO 1999).^23^ All other vibrations are considerably weaker. The P−H stretch vibration was found at 2306 cm^−1^, which is in good agreement for ν_P‐H_ vibrations in known R=PH species (R=Si{CH[(CCH_2_)CMe‐(NDipp)_2_]} 2261,^54^ R=NHC 2291,^50^ 2295, 2311 cm^−1^),^51^ while the corresponding ν_PD_ in DPCO was found at 1620 cm^−1^. Further assignment of vibrations of DPCO is severely hampered by the presence of HDO and D_2_O in the mixture, which prevent the unambiguous identification of DPCO vibrations. However, it also allows some insight into the decomposition of HPCO and DPCO in the gas phase. In both cases, not only CO is formed as evident from its ν_CO_ vibration (see Figure S22 and S24), but also H_2_O and its isotopologues.

**Figure 4:**
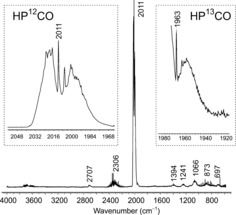
Gas‐phase IR spectrum of HPCO (298 K). Left inset: HP^12^CO ν_CO_ band. Right inset: HP^13^CO ν_CO_ band.

**Table 2:** Gas‐phase IR data for HP^12^CO and DP^12^CO.

vibration	assignment	HPCO	HPCO calc.^39^	DPCO	DPCO calc.^39^
ν_1_	ν_PH_^[a]^	2306	2278	1620	1634
ν_2_	ν_CO_	2011	2018	2012	2018
ν_3_	δ_HPC_	873	891	–	761
ν_4_	ν_PC_^[a]^	697	730	–	678
ν_5_	δ_PCO_^[b]^	368	387	–	345
ν_6_	δ_PCO_^[c]^	–	472	–	471
ν_2_ + ν_4_		2707	2663	–	2619
2 × ν_4_		1394	1411	–	1327
ν_3_ + ν_5_		1241	1225	–	1091
ν_4_ + ν_5_		1066	1081	–	1001

[a] Partially obscured by CO_2_. [b] Determined from combination and overtone bands. [c] Not observed due to low IR activity. Calculated data scaled by 0.95.

Attempts to utilize HPCO for further syntheses were hampered due to its relative instability. At low temperature, no reactions were occurred for mixtures of HPCO and acetonitrile, dimethylbutadiene, 2‐butyne, diphenylacetylene, diphenylketene, or DippOH, while upon warming to room temperature only decomposition was observed. Metal complexes like Ru(cymanthrene)Cl_2_, Cp*Ru(dppe)Cl, and Vaska's complex (Ir(PPh_3_)_2_(CO)Cl) give only proof on the presence of PH_3_, while W(dppe)_2_(N_2_)_2_ does not react with HPCO at all. No reaction was observed with Lewis acids such as B(C_6_F_5_)_3_ or GaCl_3_, which is in accord with computations since the association products are not minima on the potential energy hypersurface. Also, due to low solubility of silylium salts of the type [(Me_3_Si)_2_H][CHB_11_Cl_11_] in toluene at low temperatures and thermal instability of HPCO, no adduct formation to give the hypothetical [Me_3_SiOCPH]^+^ or [Me_3_SiO(H)CP]^+^ could be observed.

In an attempt to induce a [3+2] cycloaddition reaction between HPCO and N_3_^−^, signals for PCO^−^ were observed in the NMR spectrum. HPCO reacts with amines, for example, cyclohexylamine, to give phosphinecarboxamides, and with the carbene 1,3‐bis(2,4,6‐trimethylphenyl)imidazol‐2‐ylidene (^Mes^NHC) to give the corresponding ^Mes^NHC=PH, however, these reactions can be carried out more conveniently by treating PCO^−^ with either an amine and a proton source or an imidazolium salt, respectively.

The molecular orbitals of HPCO that are expected to influence its reactivity most are depicted in Figure 5: The HOMO−1 bears pronounced lone‐pair character on the P atom, the HOMO is dominantly π bonding between P and C and the LUMO bears antibonding character on the P−H and the C−O bond. An explanation for this surprising lack of reactivity, despite its unstable nature, can be found in the low polarity of the molecule. NBO analyses (Figure 5) reveal partial charges of HPCO of +0.033 (H), +0.025 (P), +0.344 (C), and −0.403 *e* (O) which indicates no significant polarization of the P−H bond, hence the species is only a poor nucleophile or electrophile, rendering its existence in solution possible.

**Figure 5:**
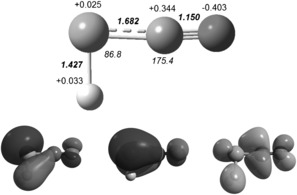
Top: Optimized structure (bold: bond lengths [Å], italics: angles [°]) of HPCO and NBO charges in *e*. Bottom: HOMO−1 (left), HOMO (middle), and LUMO (right) of HPCO.

In conclusion, metastable solutions of HPCO can be generated using standard laboratory means. This elusive reactive species has now been observed in solution for the first time. In the previously reported reactions of [Na(dioxane)_2.5_]PCO with ammonium and imidazolium salts, which yield phosphinecarboxamides (H_2_P‐C(O)‐NHR; R=H, alkyl, alkenyl, alkynyl) and carbene‐stabilized primary phosphinidenes (R=PH; R=carbene), respectively, HPCO is a plausible intermediate. Phosphinecarboxamides and carbene‐stabilized primary phosphinidenes can also be generated by reactions of HPCO with amines and NHCs, respectively.

## Experimental Section

Representative setup: A mixture of stearic acid (120 mg, 0.420 mmol) and [Na(dioxane)_*x*_]PCO (45 mg, 0.265 mmol) was heated with an oil bath in vacuo in a Schlenk tube connected to an NMR tube with 0.5 mL of an NMR solvent ([D_8_]toluene, CD_2_Cl_2_, [D_8_]THF) frozen in a liquid nitrogen cooling trap. At 60 °C, gas evolution commenced. The temperature was increased stepwise (5 K every 15 min) until 120 °C were reached. In the NMR tube, a yellow condensate formed. The nitrogen cooling bath was removed and quickly replaced by a dry ice/2‐propanol cooling bath (−78 °C). Upon thawing, the solvent dissolved most of the yellow condensate, forming a yellow solution which was subjected to NMR experiments.

*In memory of Gerd Becker*

## Conflict of interest

The authors declare no conflict of interest.

## Supporting information

As a service to our authors and readers, this journal provides supporting information supplied by the authors. Such materials are peer reviewed and may be re‐organized for online delivery, but are not copy‐edited or typeset. Technical support issues arising from supporting information (other than missing files) should be addressed to the authors.

Supplementary
