# Supplementary material for: HPCO—A Phosphorus‐Containing Analogue of Isocyanic Acid
Source: Angew Chem Int Ed Engl. 2017 Mar 2;56(14):3911–5. doi: 10.1002/anie.201700368 (PMC5396272; doi:10.1002/anie.201700368)
Supplement: Supplementary file 1 — Supplementary [file ANIE-56-3911-s001.pdf]

Angewandte Chemie
Eine Zeitschrift der Gesellschaft Deutscher Chemiker

Supporting Information

HPCO—A Phosphorus-Containing Analogue of Isocyanic Acid

*Alexander Hinz, René Labbow, Chris Rennick, Axel Schulz, and Jose M. Goicoechea**

anie_201700368_sm_miscellaneous_information.pdf

Supporting Information

1. Experimental section 2

1.1 HPCO 3

NMR spectra of HPCO 4

NMR spectra of DPCO 10

Gas-phase IR spectra 18

1.2 [D_1]-stearic acid 22

2. Computational data 24

3.1 HPCO and DPCO 24

3.1.1 HPCO – displacement vectors for the vibrations 25

3.1.2 DPCO – displacement vectors for the vibrations 26

3.1.3 HPCO – vibrational analysis 27

3.1.4 DPCO – vibrational analysis 37

3.2 Isomers of HPCO 46

3.3 Isomers of $(HPCO)_2$ 47

3.4 Isomers of $(HPCO)_3$ 52

3.5 Isomers of $(HPCO)_4$ 61

4. References 91

1. Experimental section

General synthetic methods. All reactions and product manipulations were carried out under an inert atmosphere of argon or dinitrogen using standard Schlenk-line or glovebox techniques (MBraun UNIlab glovebox maintained at < 0.1 ppm H_2O and < 0.1 ppm O_2).

[Na(dioxane)]PCO was prepared according to a literature procedure, but dried at 60 °C for 8 hours to reduce the dioxane content.[1] Hexane (hex; Sigma-Aldrich, HPLC grade) was purified using an MBraun SPS-800 solvent system. Tetrahydrofuran (THF; Sigma-Aldrich, HPLC grade) was distilled over sodium metal/benzophenone. D_2O (Acros, 99.8%) and C_6D_6 (Sigma-Aldrich, 99.5%) were used as received. Stearic acid (Sigma-Aldrich, 95%) and Me_3SiCl (Sigma-Aldrich, 99.5%) were used as received. NEt_3 (Sigma-Aldrich, 99.5%) was distilled from CaH_2 and degassed prior to use. [D_8]-toluene (Sigma-Aldrich, 99.5%), CD_2Cl_2 (Sigma-Aldrich, 99.5%), [D_8]-THF (Sigma-Aldrich, 99.5%) were dried over CaH_2 and stored under argon in gas-tight ampoules over activated 3 Å molecular sieves.

Characterization techniques: ^{1}H, ^{1}H{^{31}P}, ^{2}H, ^{13}C, ^{13}C{^{1}H}, ^{13}C-DEPT135, ^{31}P, and ^{31}P{^{1}H} NMR spectra were acquired at 223 K at 400.2 (^{1}H), 100.6 (^{13}C) and 162.0 (^{31}P) MHz, respectively, on a Bruker Avance 300 HD Ascend NMR spectrometer if not stated otherwise. ^{1}H and ^{13}C NMR spectra were referenced to the most downfield solvent resonance.[2] ^{31}P spectra were externally referenced to an 85% solution of H_3PO_4 in H_2O (δ = 0 ppm). Gas-phase IR spectra were recorded at ambient conditions in cells with 10 cm path length and NaCl windows.

1.1 HPCO

Representative setup: A mixture of stearic acid or [D_1]-stearic acid (120 mg, 0.420 mmol) and [Na(dioxane)]PCO (45 mg, 0.265 mmol) was heated with an oil bath in vacuo in a Schlenk tube connected to an NMR tube with 0.5 ml of an NMR solvent ([D_8]-toluene, CD_2Cl_2, [D_8]-THF) frozen in a liquid nitrogen cooling trap. At 60 °C, gas evolution commenced in. The temperature was increased stepwise (5 K every 15 min) until 120 °C were reached. In the NMR tube, a yellow condensate formed. The nitrogen cooling bath was removed and quickly replaced by a dry ice/isopropanol cooling bath (–78 °C). Upon thawing, the solvent dissolved most of the yellow condensate, forming a yellow solution which was subjected to NMR experiments.

^{1}HP^{12}CO

^{1}H NMR ([D_8]-toluene, 223 K): 0.25 ($^{1}J_{HP}$ = 188 Hz). **^{31}P NMR** ([D_8]-toluene, 223 K): –316.7 ($^{1}J_{PH}$ = 188 Hz). **^{13}C NMR** ([D_8]-toluene, 223 K): 201.4 ($^{1}J_{CP}$ = 102, $^{2}J_{CH}$ = 10.5 Hz). **^{1}H NMR** (CD_2Cl_2, 223 K): 1.09 ($^{1}J_{HP}$ = 189 Hz). **^{31}P NMR** (CD_2Cl_2, 223 K): –312.8 ($^{1}J_{PH}$ = 189 Hz). **^{13}C NMR** (CD_2Cl_2, 223 K): 201.5 ($^{1}J_{CP}$ = 98 Hz). **^{1}H NMR** ([D_8]-THF, 193 K): 1.32 ($^{1}J_{HP}$ = 192 Hz). **^{31}P NMR** ([D_8]-THF, 193 K): –316.7 ($^{1}J_{PH}$ = 192 Hz). **^{13}C NMR** ([D_8]-THF, 193 K): 202.2 ($^{1}J_{CP}$ = 101 Hz). **IR** (gas, cm^{-1}): 2707, 2306, 2011 (vs), 1394, 1241, 1066, 873, 697, 473, 389.

^{1}HP^{13}CO

^{31}P NMR ([D_8]-toluene, 223 K): –316.7 ($^{1}J_{PH}$ = 188, $^{1}J_{PC}$ = 102 Hz). **^{31}P NMR** (CD_2Cl_2, 223 K): –312.8 ($^{1}J_{PH}$ = 189, $^{1}J_{PC}$ = 98 Hz). **IR** (gas, cm^{-1}): 1963.

^{2}HP^{12}CO

^{2}H NMR ([D_8]-toluene, 223 K): 0.29 ($^{1}J_{DP}$ = 29.6 Hz). **^{31}P NMR** ([D_8]-toluene, 223 K): –317.7 ($^{1}J_{PD}$ = 29.5 Hz). **^{13}C NMR** ([D_8]-toluene, 223 K): 202.05 ($^{1}J_{CP}$ = 101, $^{2}J_{CD}$ = 1.55 Hz). **^{2}H NMR** (CD_2Cl_2, 223 K): 1.15 ($^{1}J_{DP}$ = 29.8 Hz). **^{31}P NMR** (CD_2Cl_2, 223 K): –314.3 ($^{1}J_{PD}$ = 29.9 Hz). **^{13}C NMR** (CD_2Cl_2, 223 K): 201.5 ($^{1}J_{CP}$ = 101, $^{2}J_{CD}$ = 1.55 Hz). **^{31}P NMR** ([D_8]-THF, 193 K): –317.8 ($^{1}J_{PD}$ = 29.9 Hz). **IR** (gas, cm^{-1}): 3488/3454, 2596/2553/2522, 2012 (vs), 1620, 1423, 1193, 1065, 852, 724, 341.

^{2}HP^{13}CO

^{31}P NMR ([D_8]-toluene, 223 K): –317.9 ($^{1}J_{PD}$ = 29.5, $^{1}J_{PC}$ = 101 Hz). **^{31}P NMR** (CD_2Cl_2, 223 K): –314.6 ($^{1}J_{PD}$ = 29.5, $^{1}J_{PC}$ = 100 Hz). **IR** (gas, cm^{-1}): 1964.

NMR spectra of HPCO

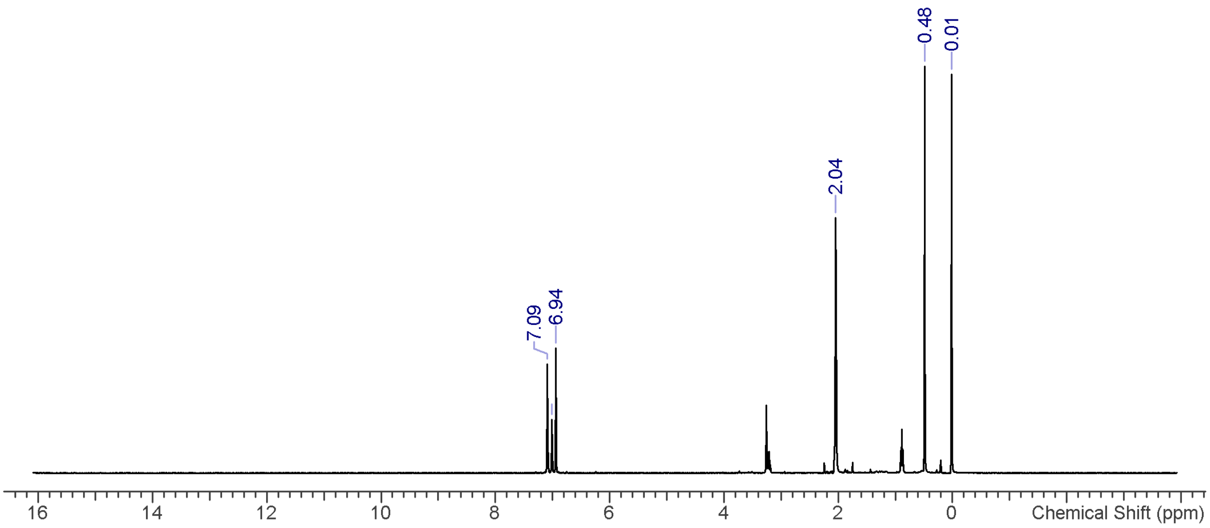

Figure S1. ^{1}H NMR spectrum of HPCO in [D_8]-toluene.

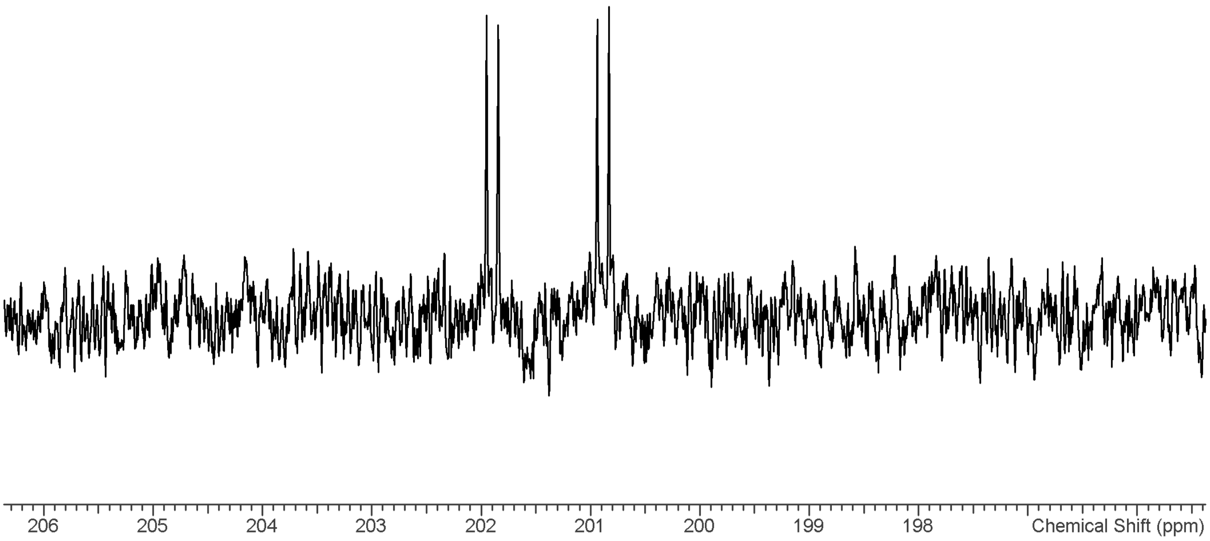

Figure S2. ^{13}C NMR spectrum of HPCO in [D_8]-toluene.

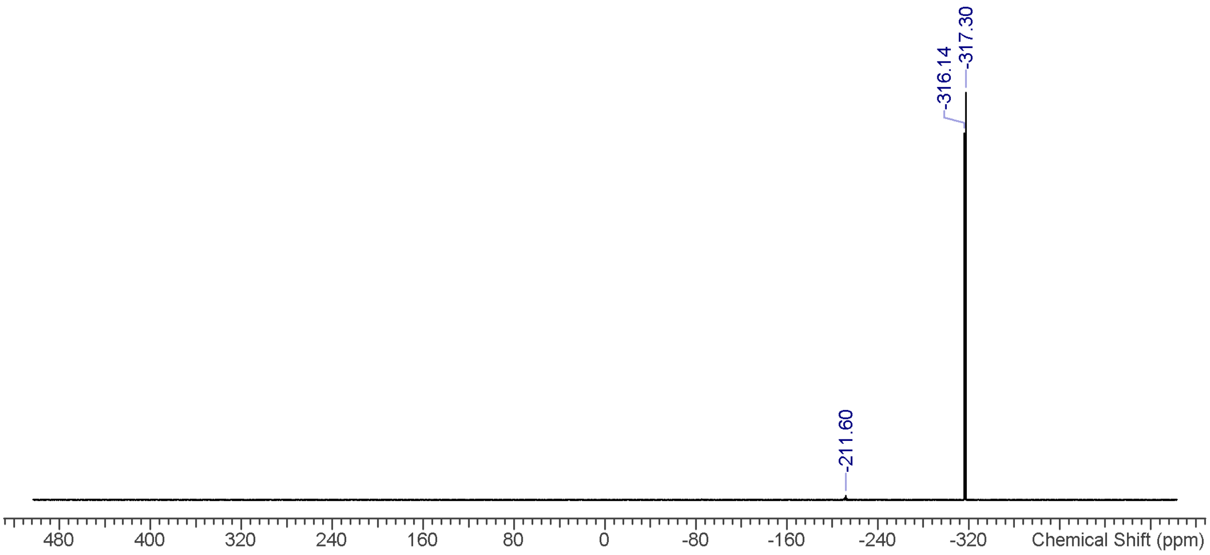

Figure S3. ^{31}P NMR spectrum of HPCO in [D_8]-toluene.

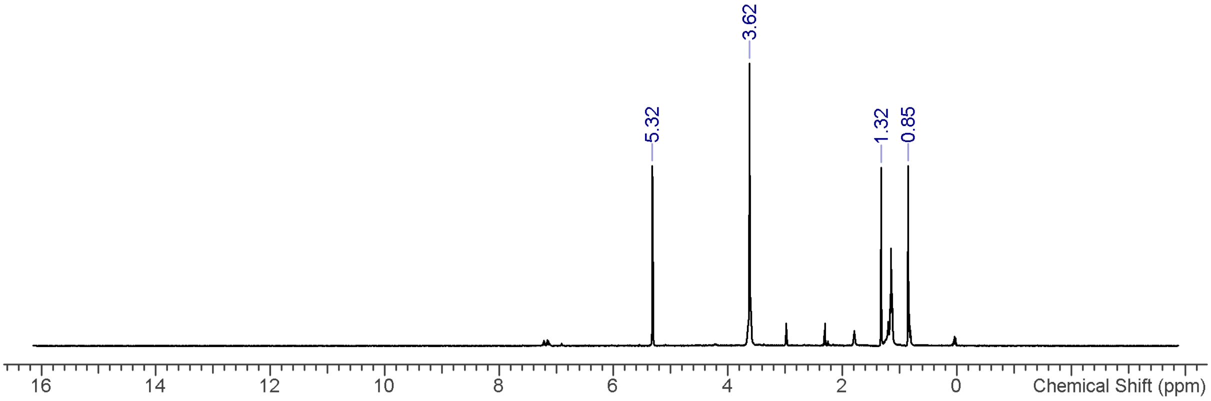

Figure S4. ^{1}H NMR spectrum of HPCO in CD_2Cl_2.

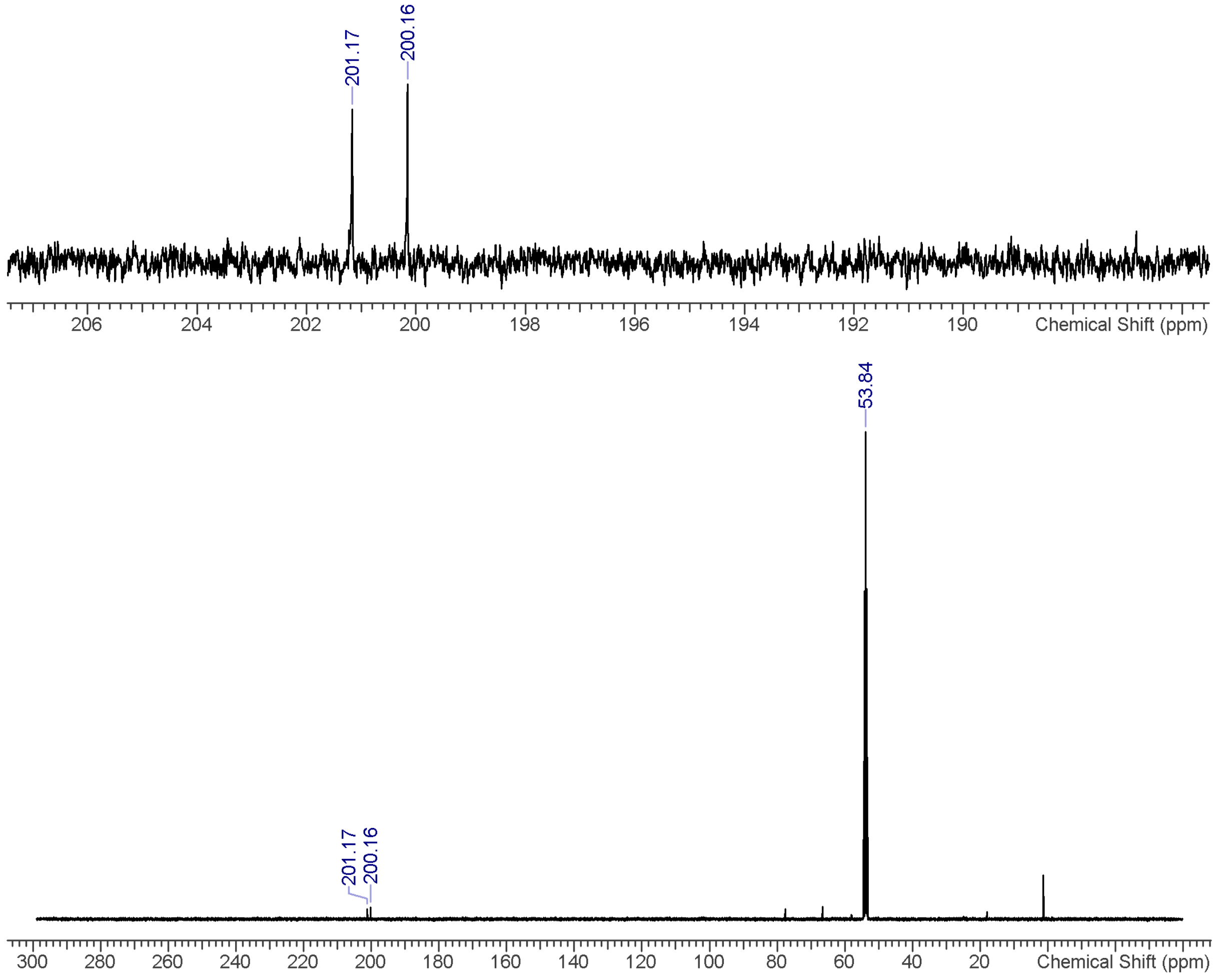

Figure S5. ^{13}C NMR spectrum of HPCO in CD_2Cl_2.

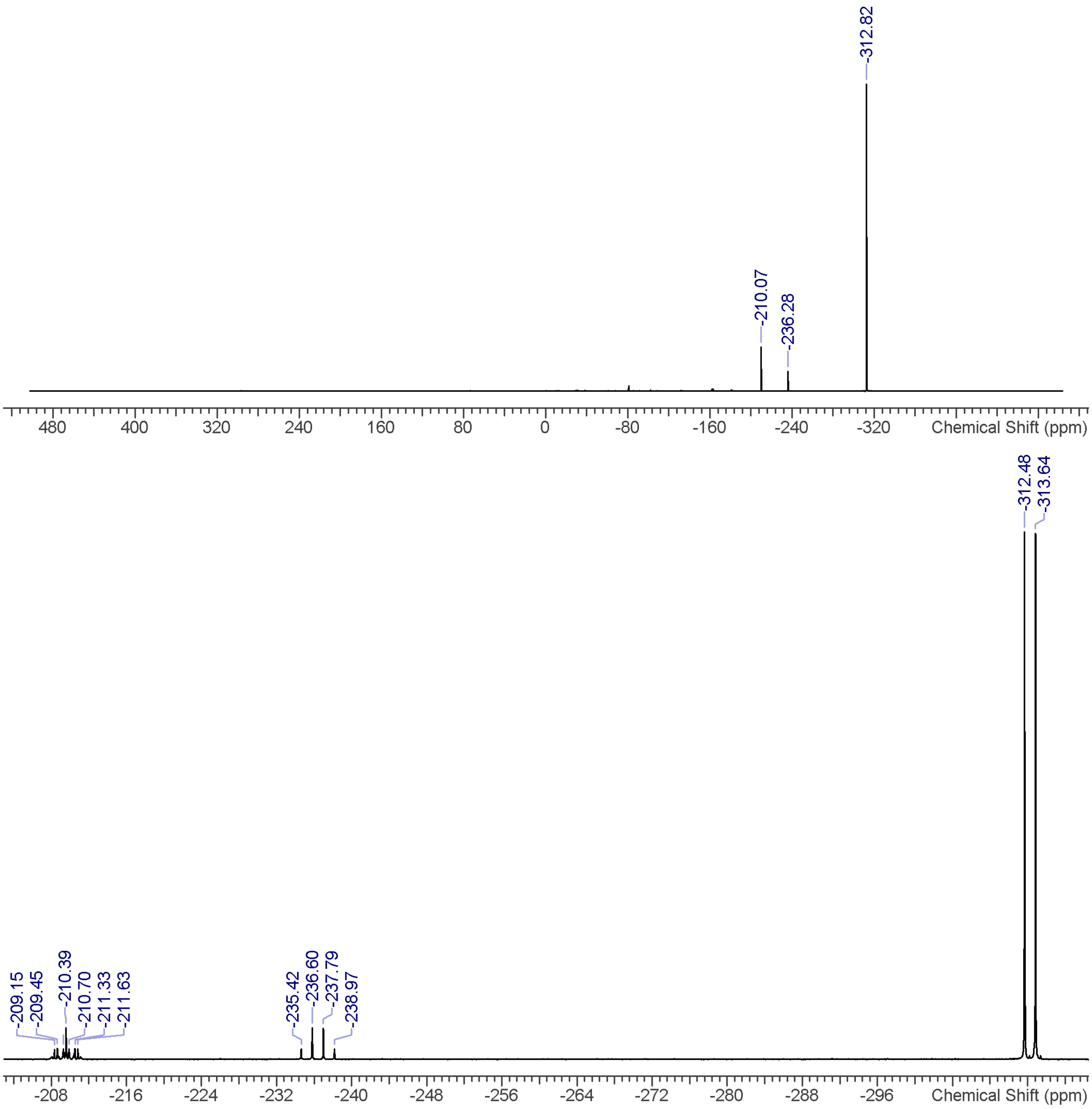

Figure S6. ^{1}H NMR spectrum of HPCO in CD_2Cl_2.

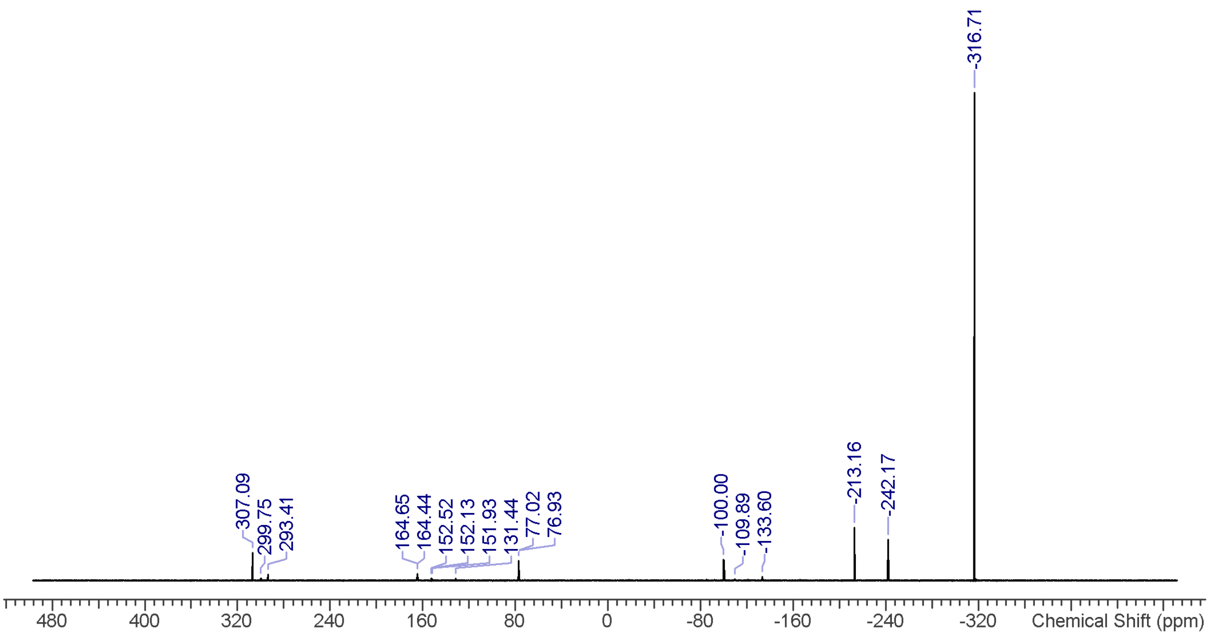

Figure S7. ^{31}P NMR spectrum of HPCO in [D_8]-THF of the purest sample of HPCO in THF that could be obtained.

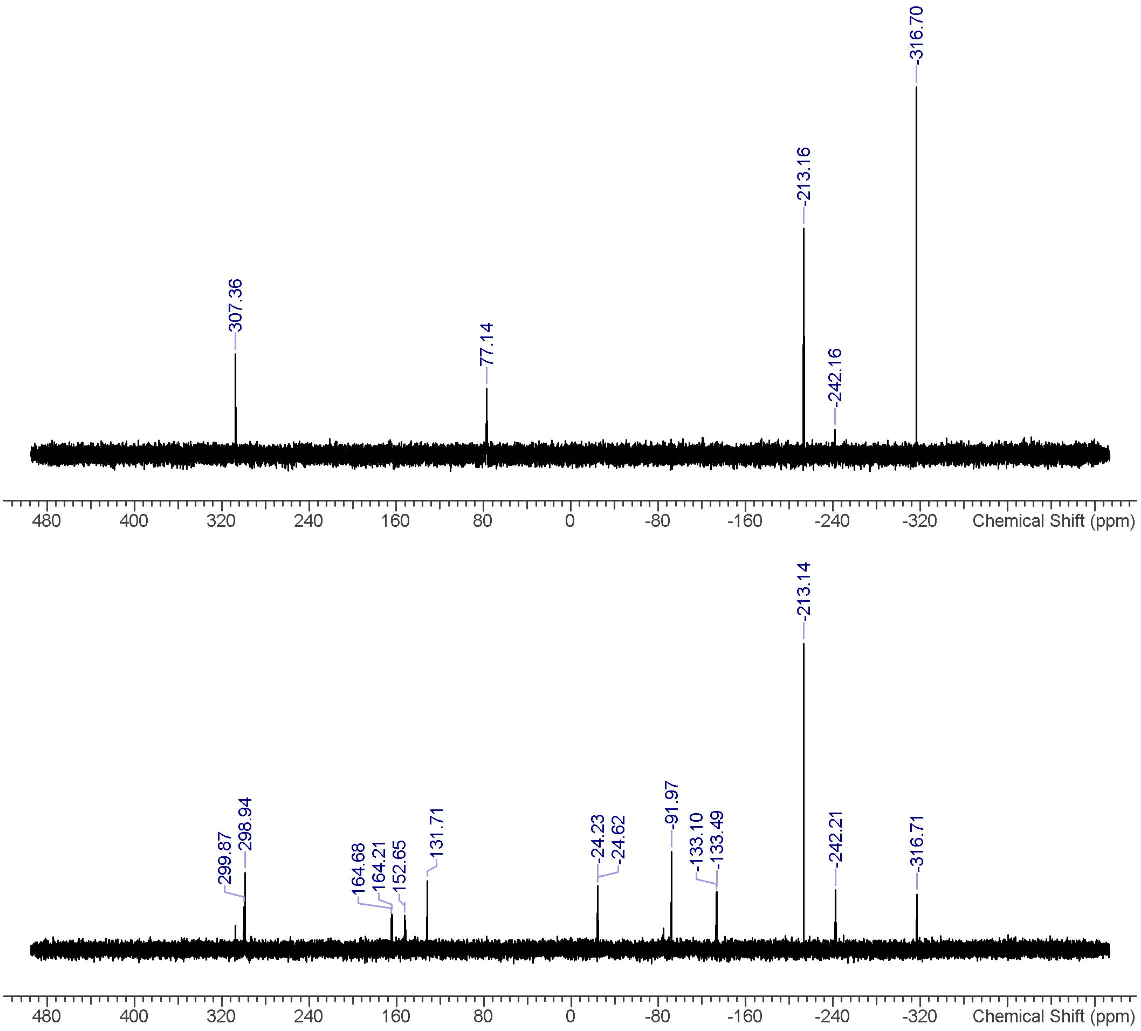

Figure S8. ^{31}P NMR spectrum of a dilute solution of HPCO in [D_8]-THF (500 MHz spectrometer). Top: only HPCO and $(HPCO)_2$ present in solution. Bottom: tetramer and other decomposition products present in solution.

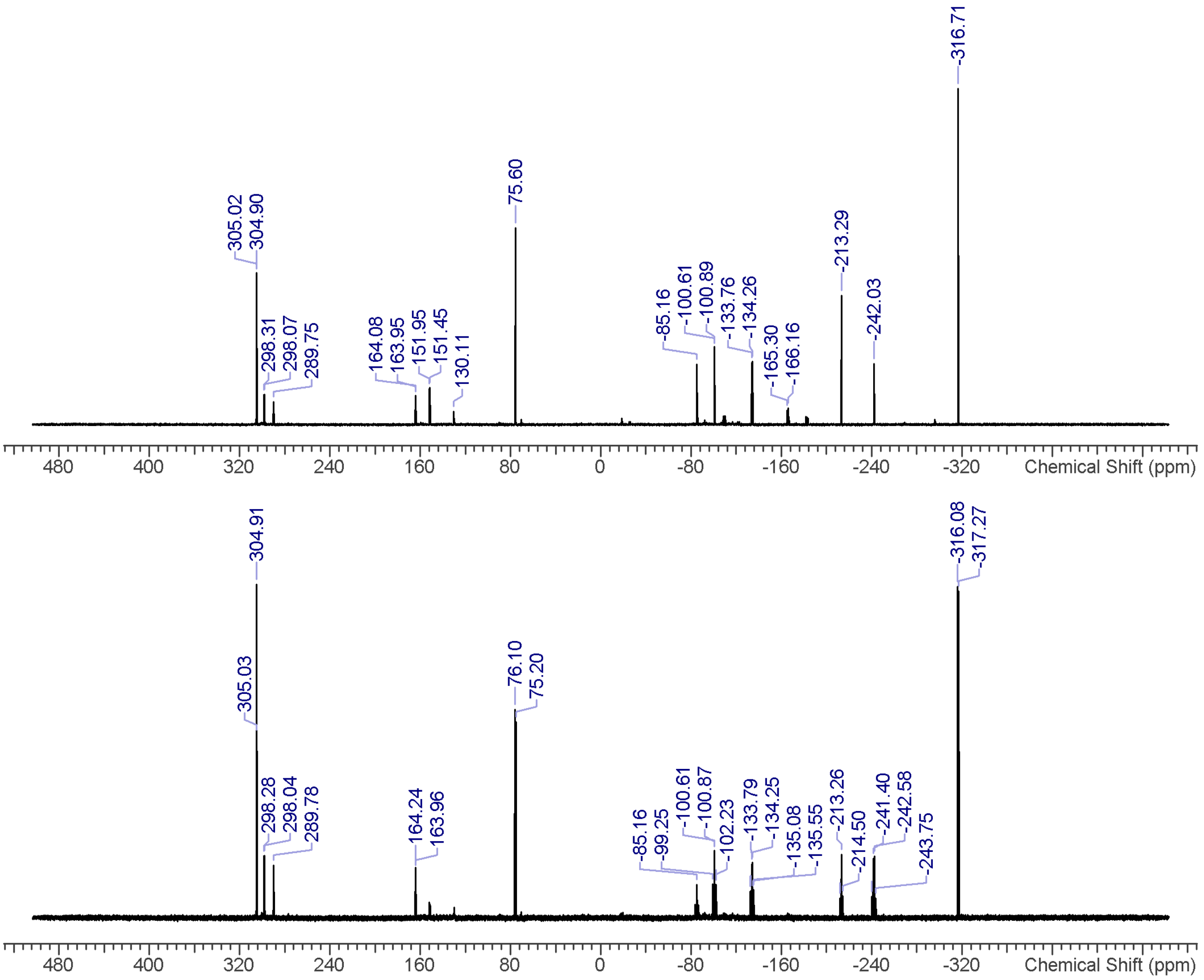

Figure S9. ^{31}P NMR spectrum of a concentrated solution of HPCO in [D_8]-THF.

NMR spectra of DPCO

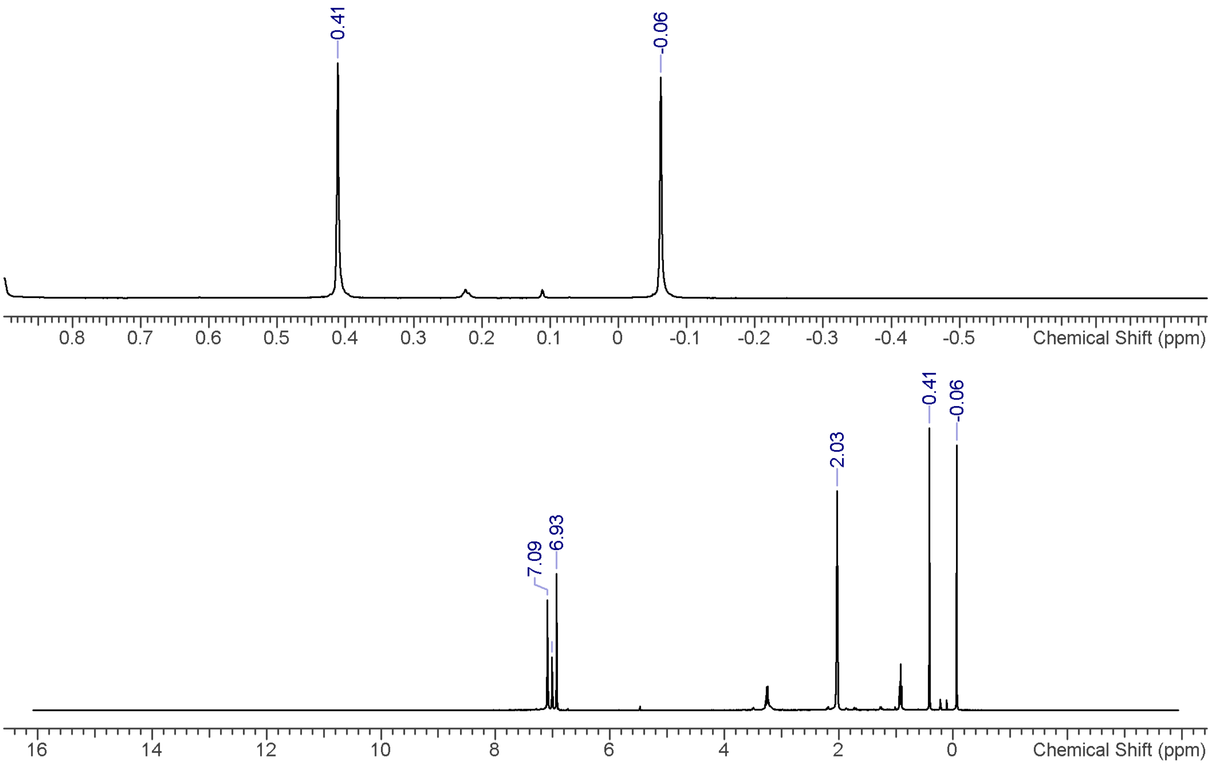

Figure S10. ^1H NMR spectrum of a mixture of HPCO and DPCO in [D_8]-toluene at 223 K.

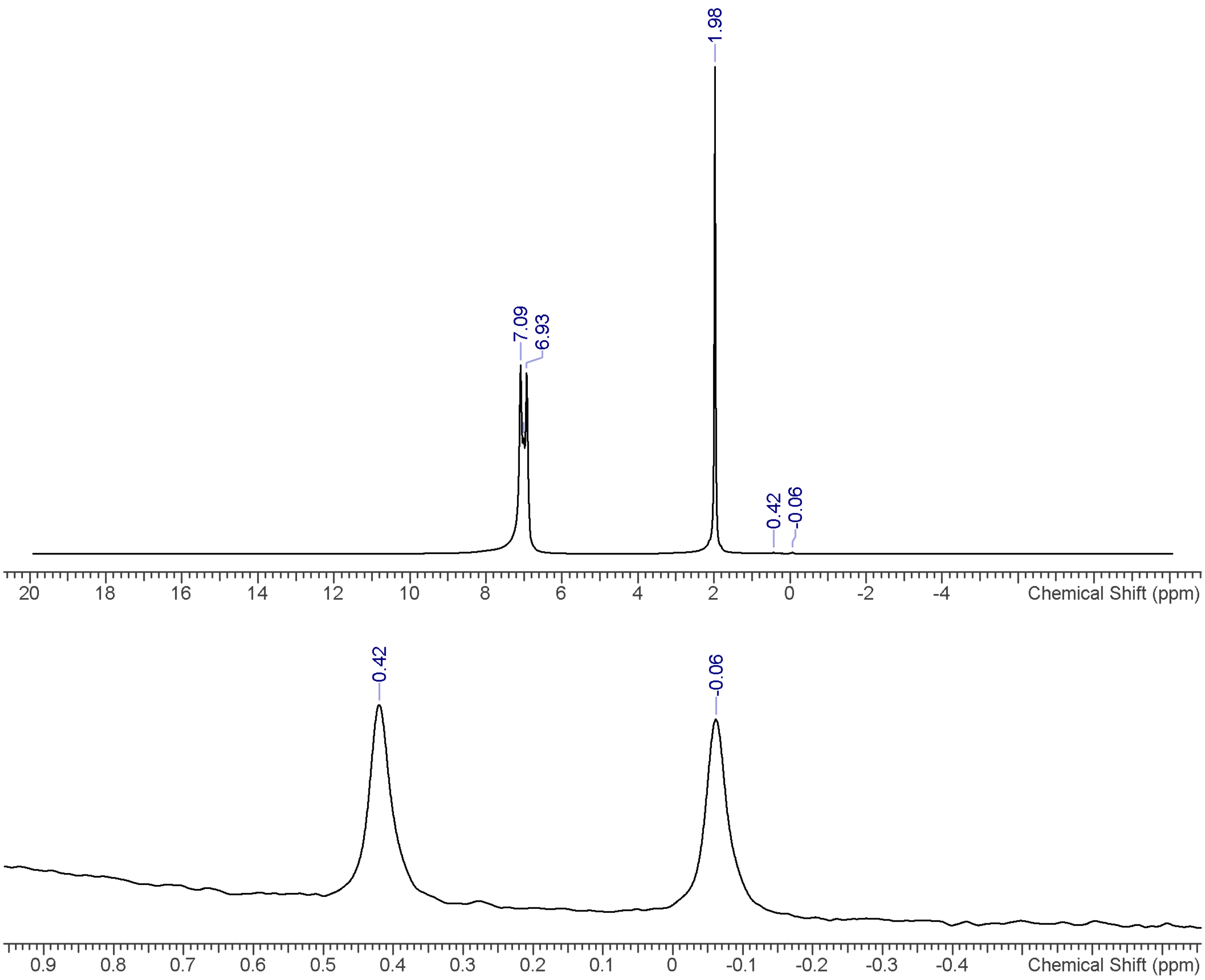

Figure S11. ^{2}H NMR spectrum of a mixture of HPCO and DPCO in [D_8]-toluene at 223 K.

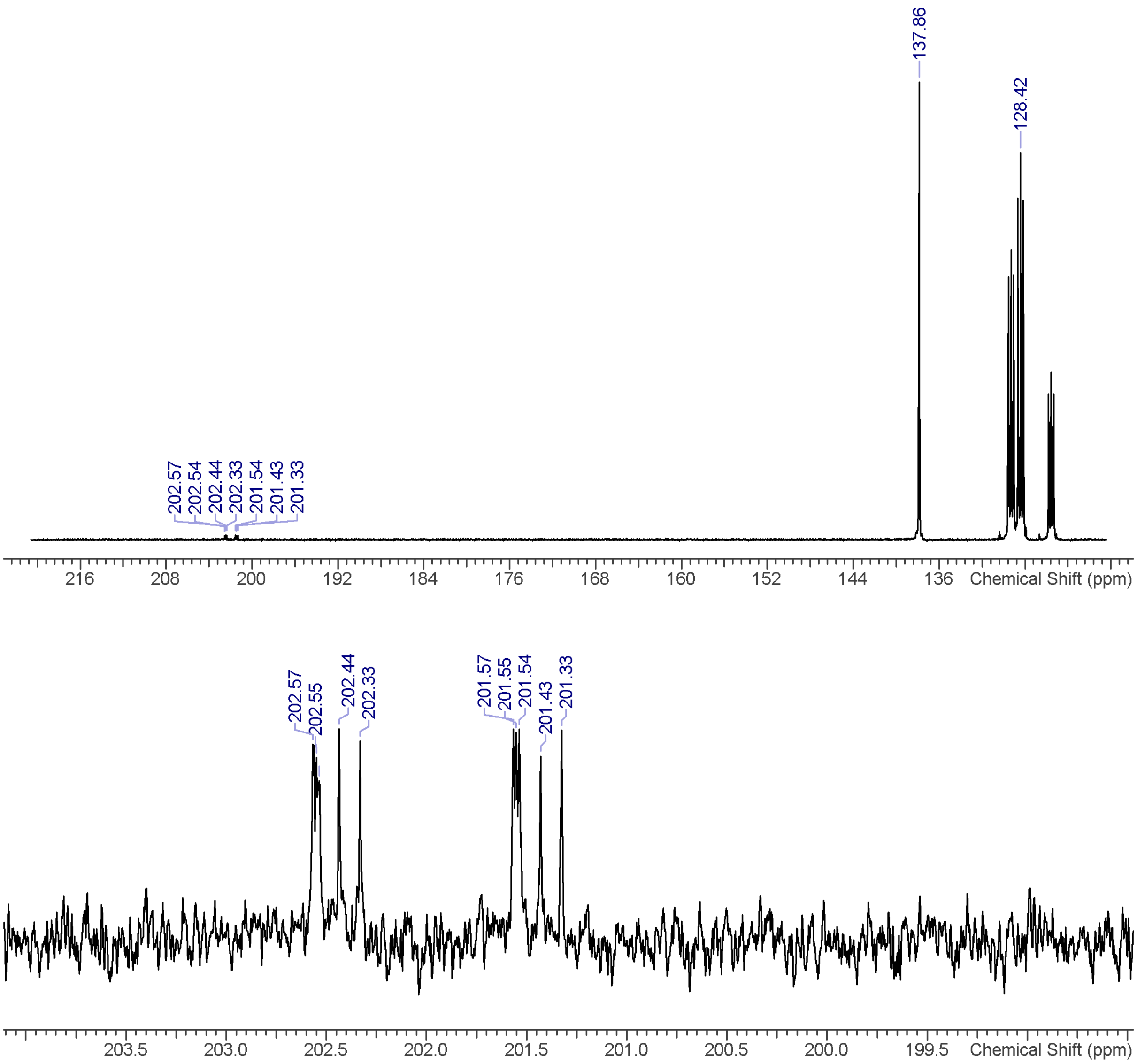

Figure S12. ^{13}C NMR spectrum of a mixture of HPCO and DPCO in [D_8]-toluene.

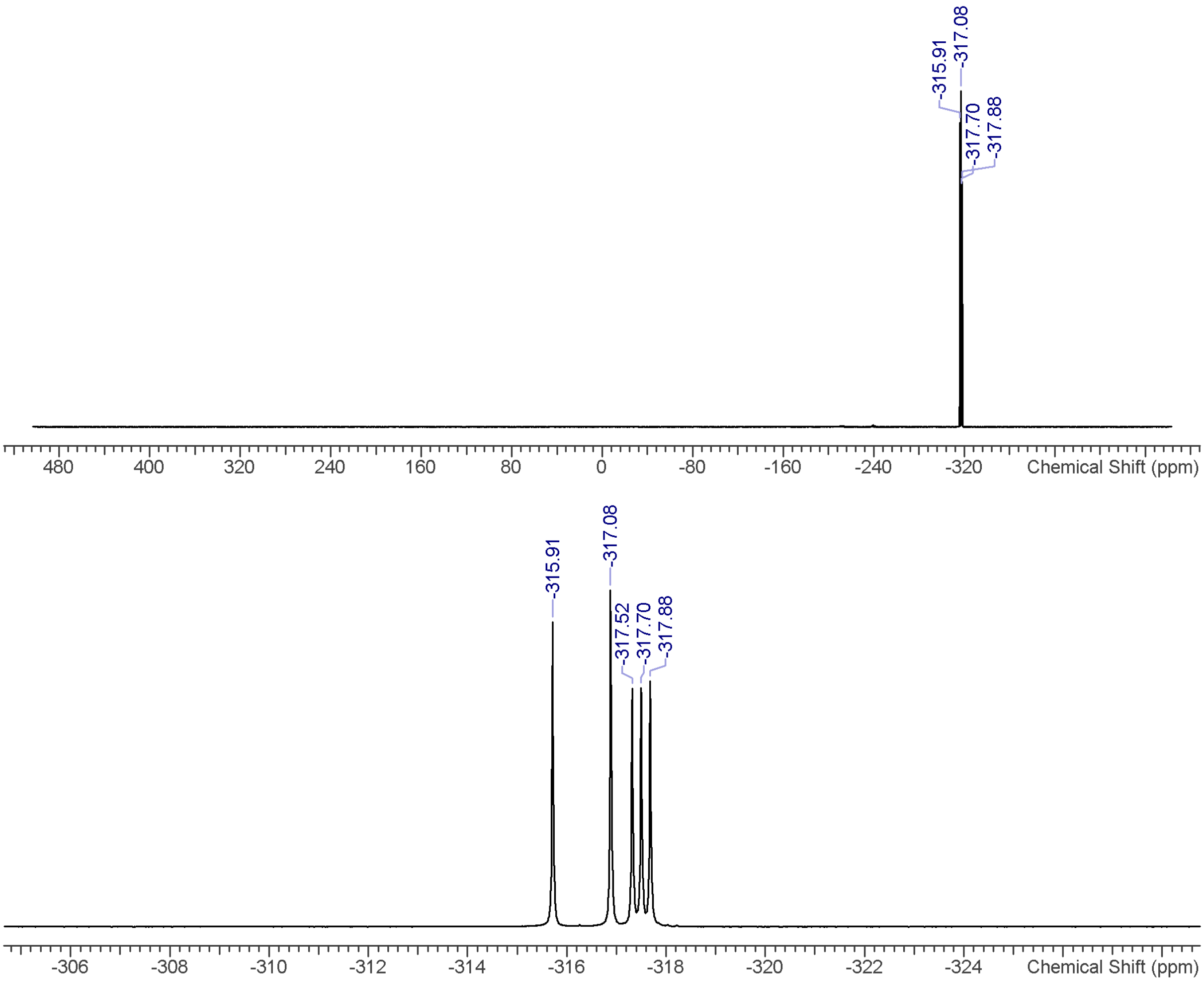

Figure S13. ^{31}P NMR spectrum of a mixture of HPCO and DPCO in [D_8]-toluene.

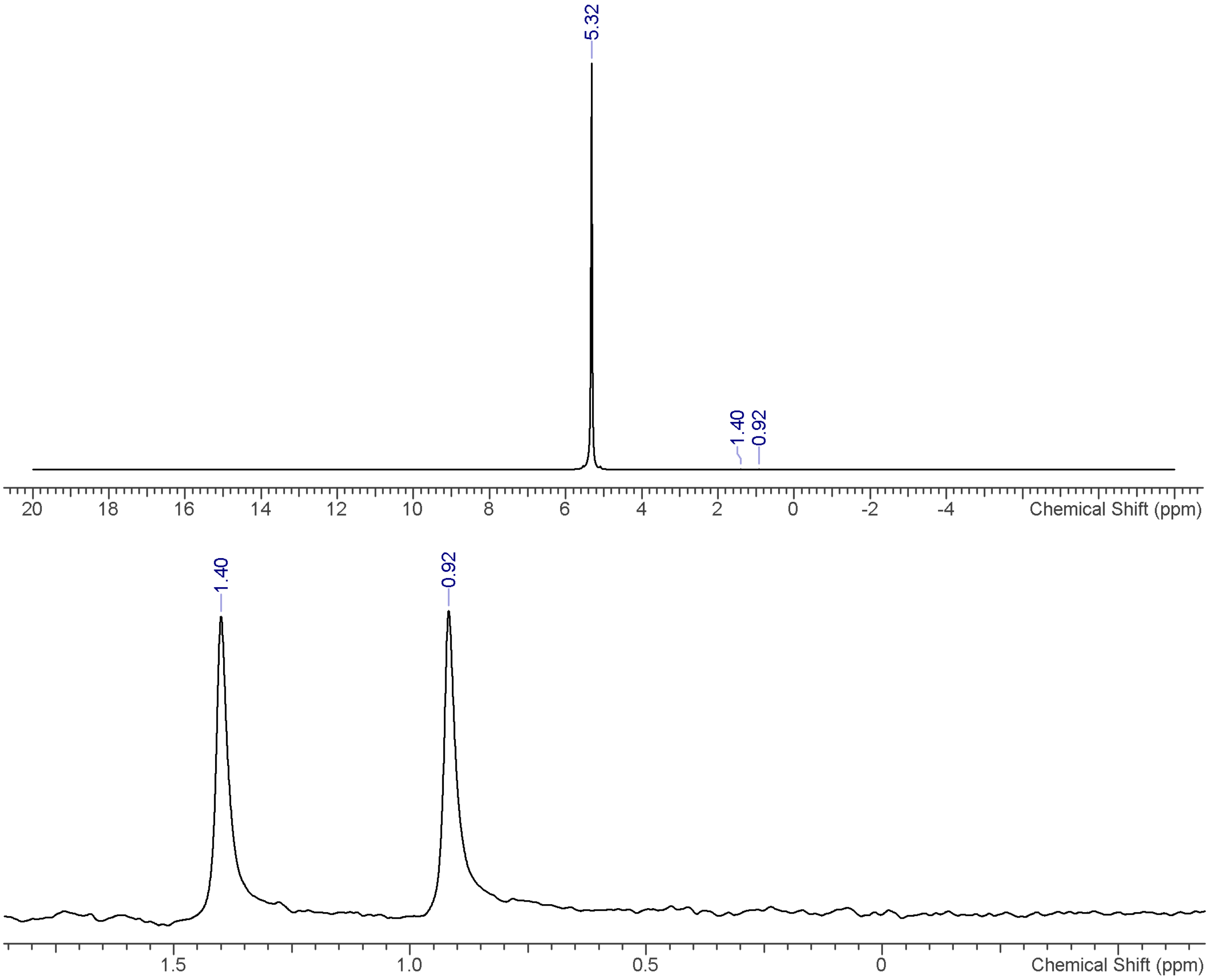

Figure S14. ^{2}H NMR spectrum of a mixture of HPCO and DPCO in CD_2Cl_2.

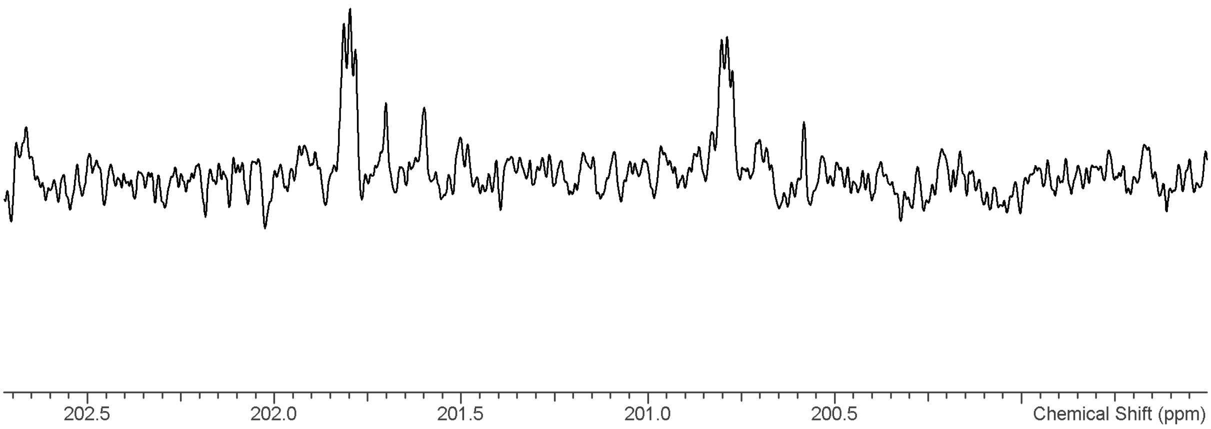

Figure S15. ^{13}C NMR spectrum of a mixture of HPCO and DPCO in CD_2Cl_2.

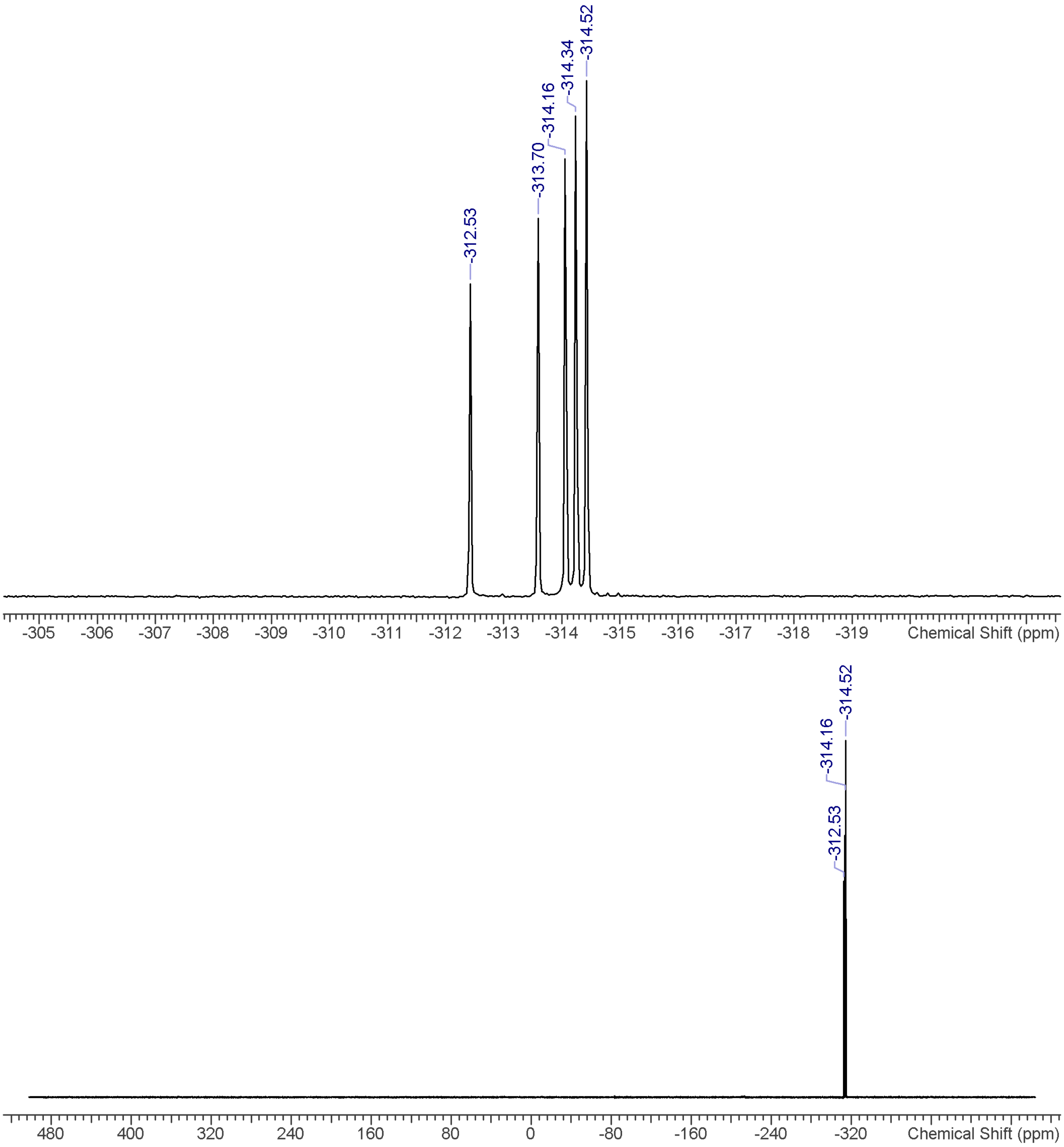

Figure S16. ^{31}P NMR spectrum of a mixture of HPCO and DPCO in CD_2Cl_2.

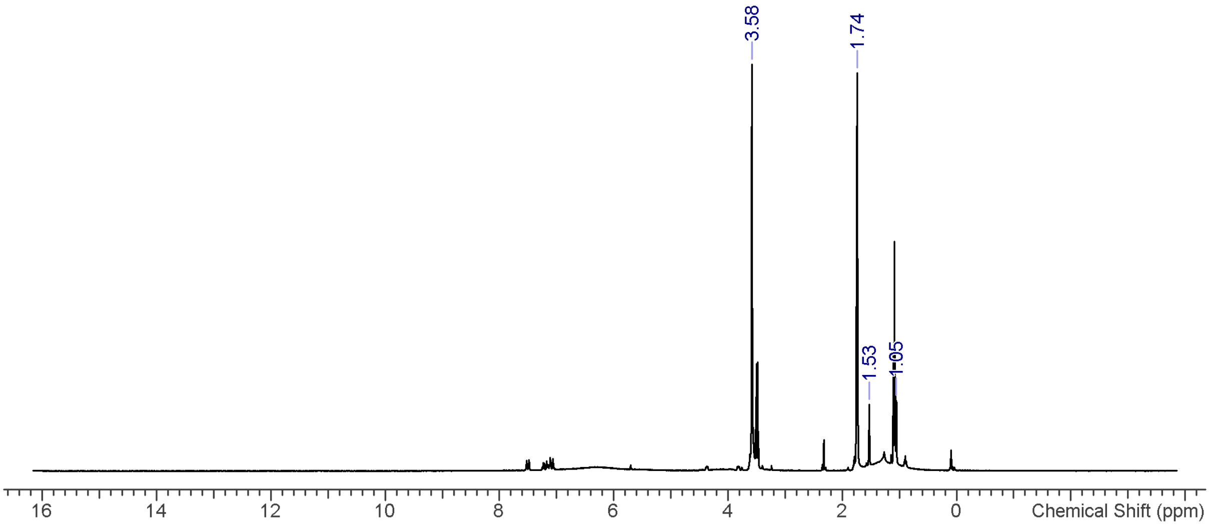

Figure S17. ^{1}H NMR spectrum of a mixture of HPCO and DPCO in [D_8]-THF at 193 K.

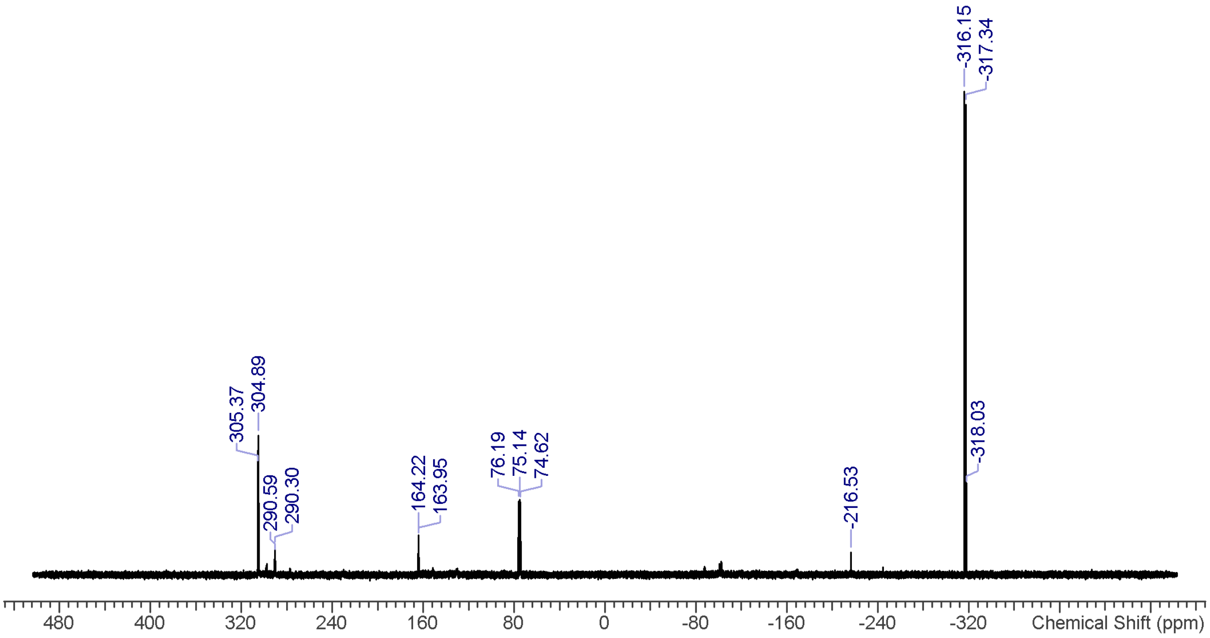

Figure S18. ^{31}P NMR spectrum of a mixture of HPCO and DPCO in [D_8]-THF at 193 K.

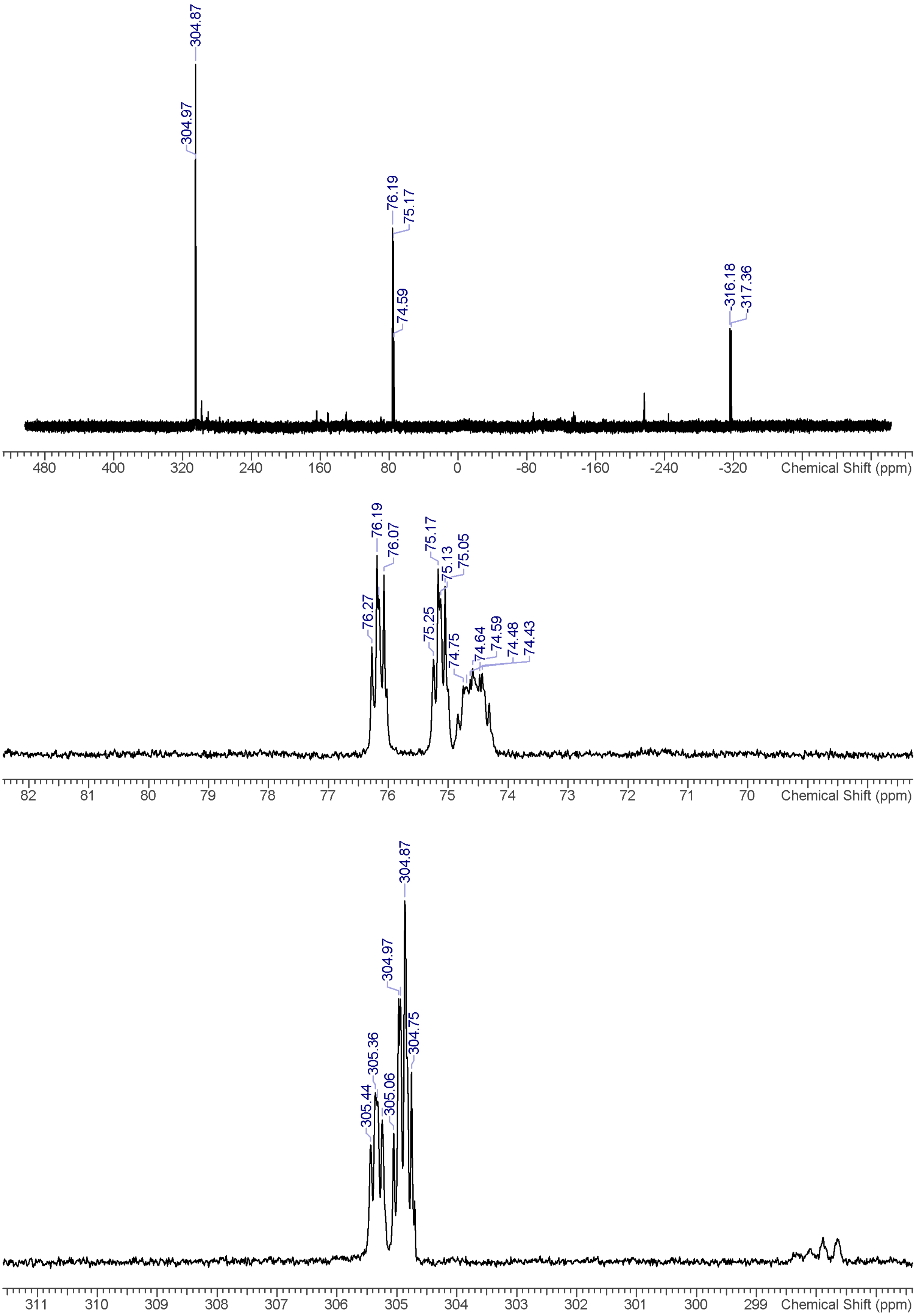

Figure S19. ^{1}H NMR spectrum of a mixture of HPCO and DPCO in [D$_8$]-THF after 2 hours at 193 K.

Gas-phase IR spectra

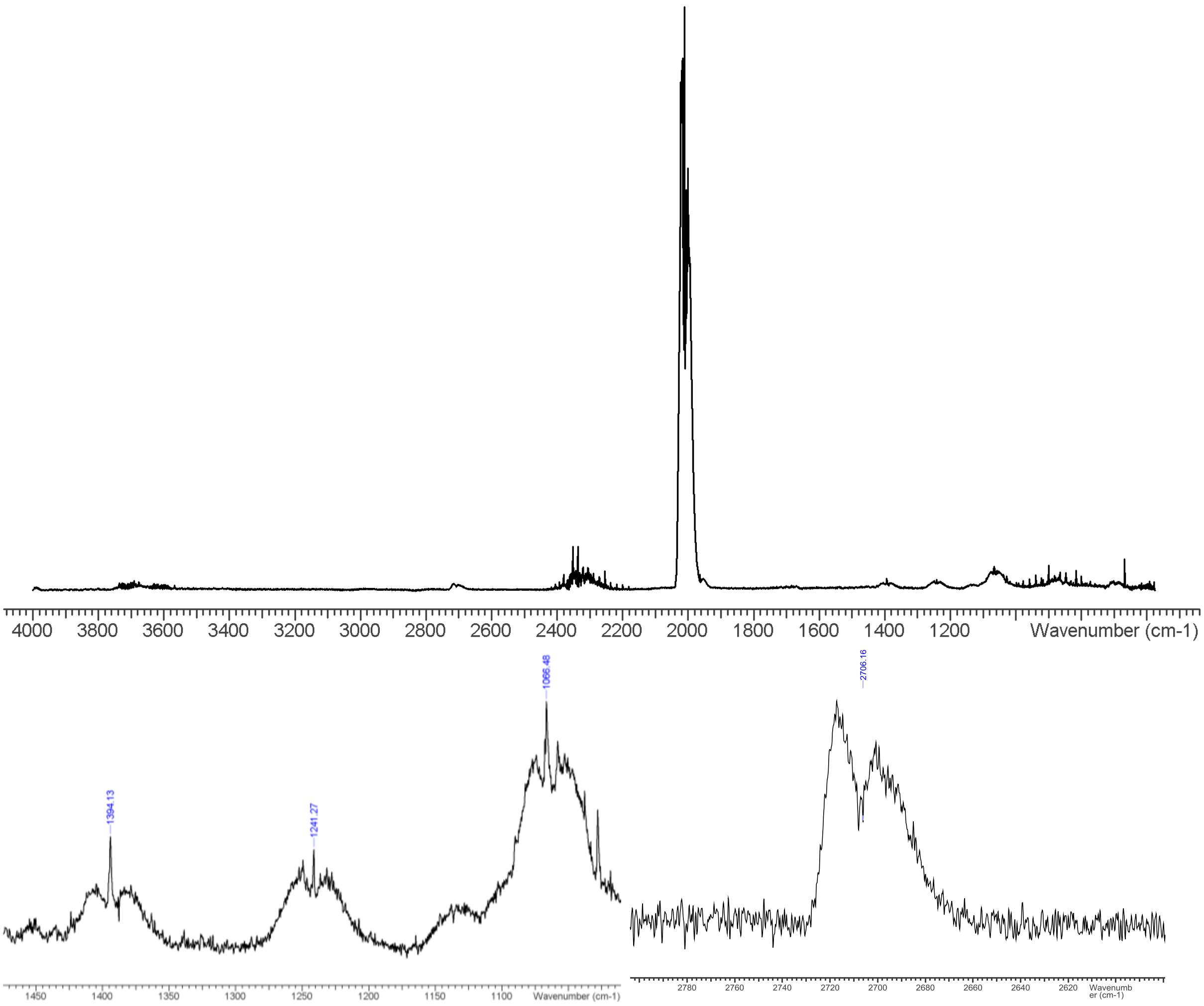

Figure S20. Processed gas-phase IR spectrum of HPCO (top) and magnified details (bottom).

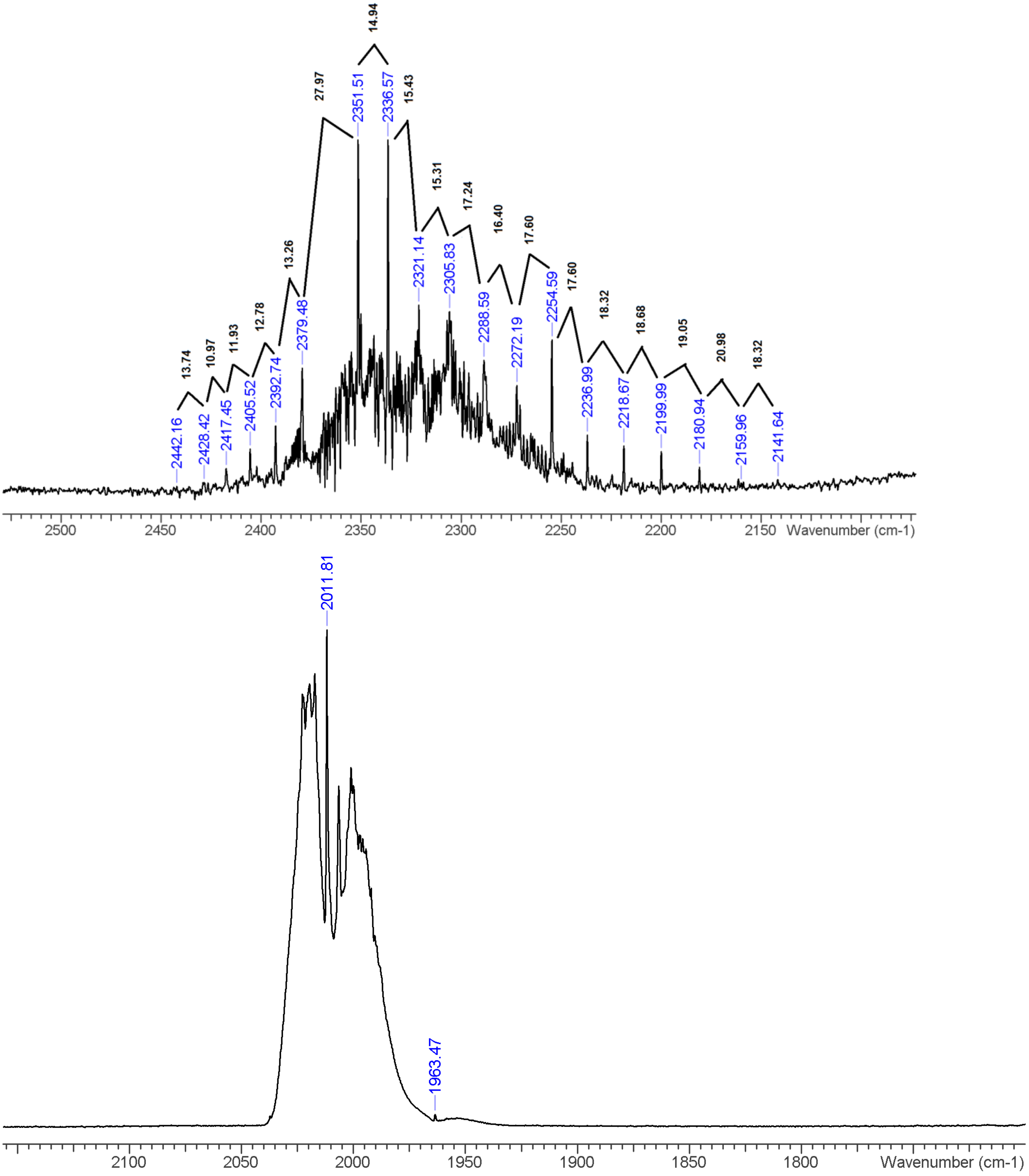

Figure S21. Processed gas-phase IR spectrum of HPCO, ν_{PH} (top) and ν_{PH} (bottom) range.

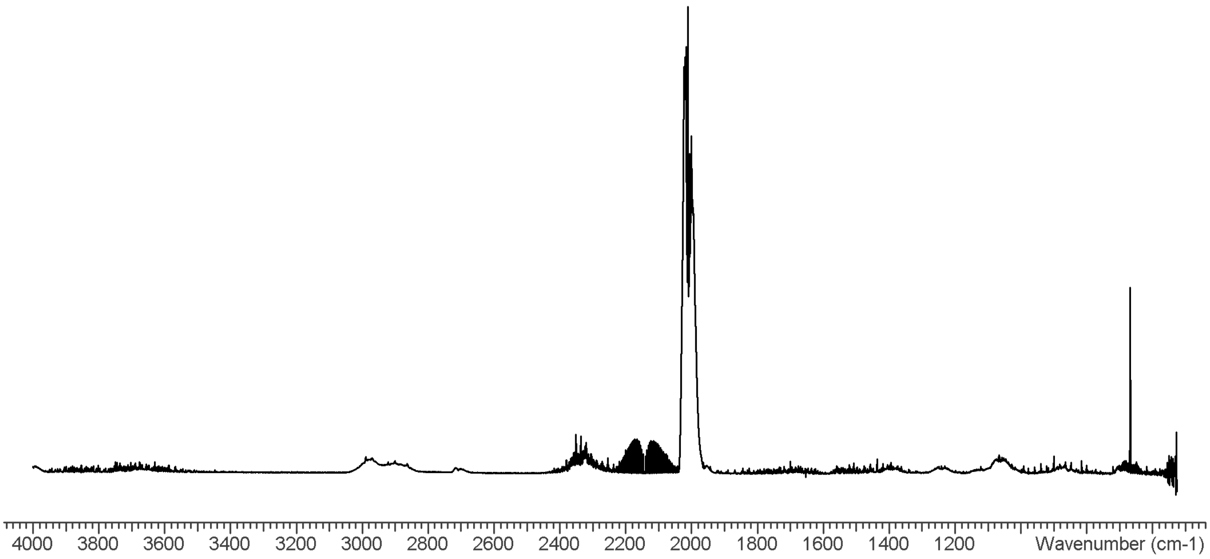

Figure S22. Raw gas-phase IR spectrum of HPCO.

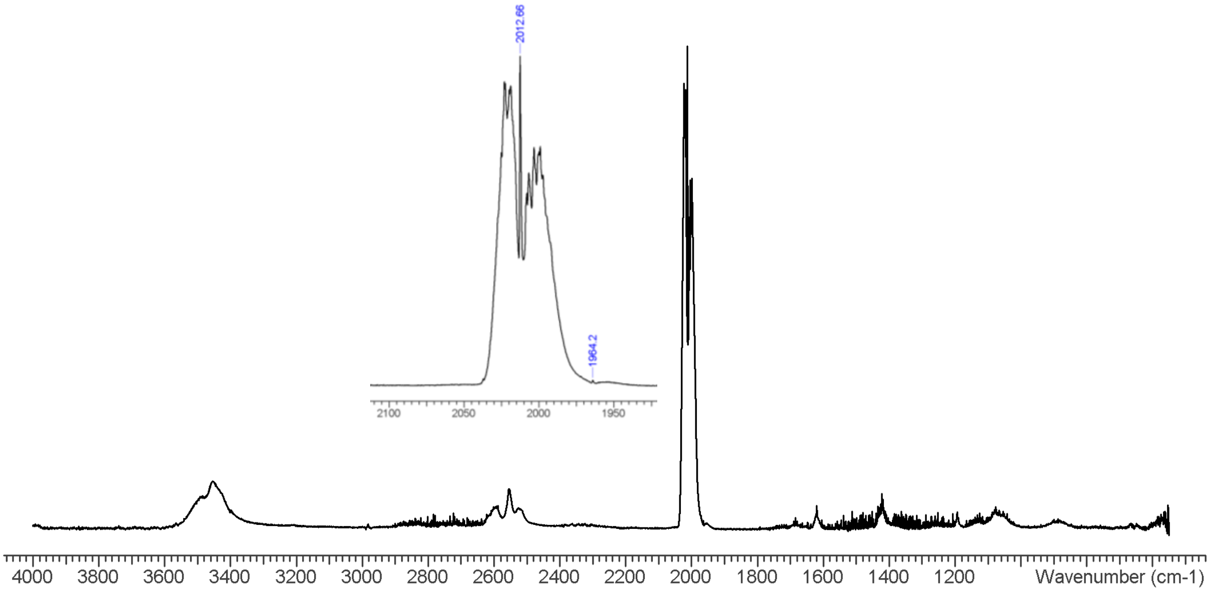

Figure S23. Processed gas-phase IR spectrum of DPCO.

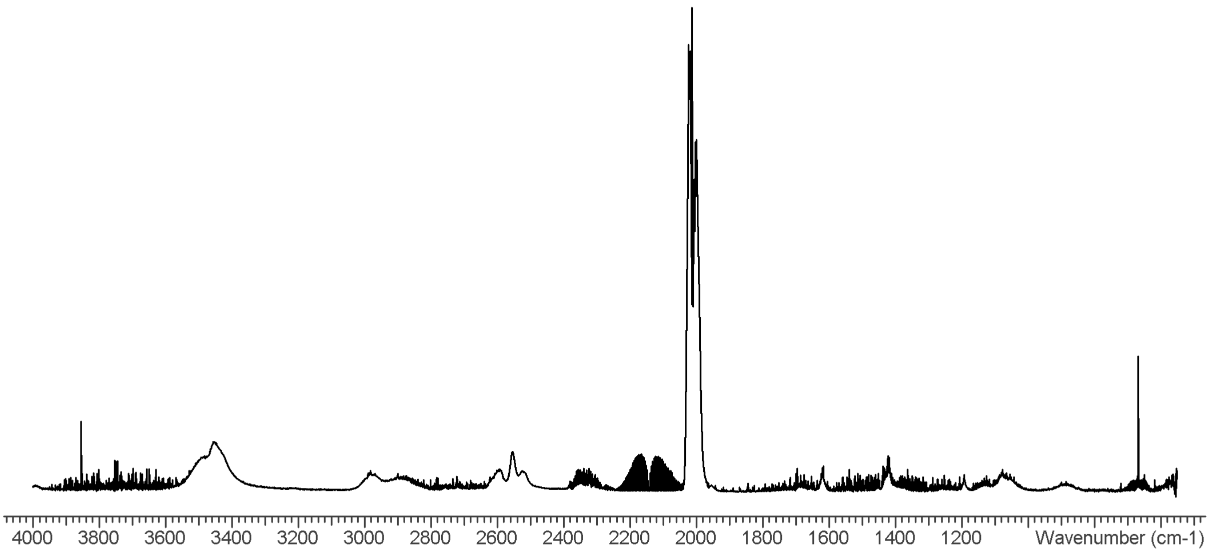

Figure S24. Raw gas-phase IR spectrum of DPCO.

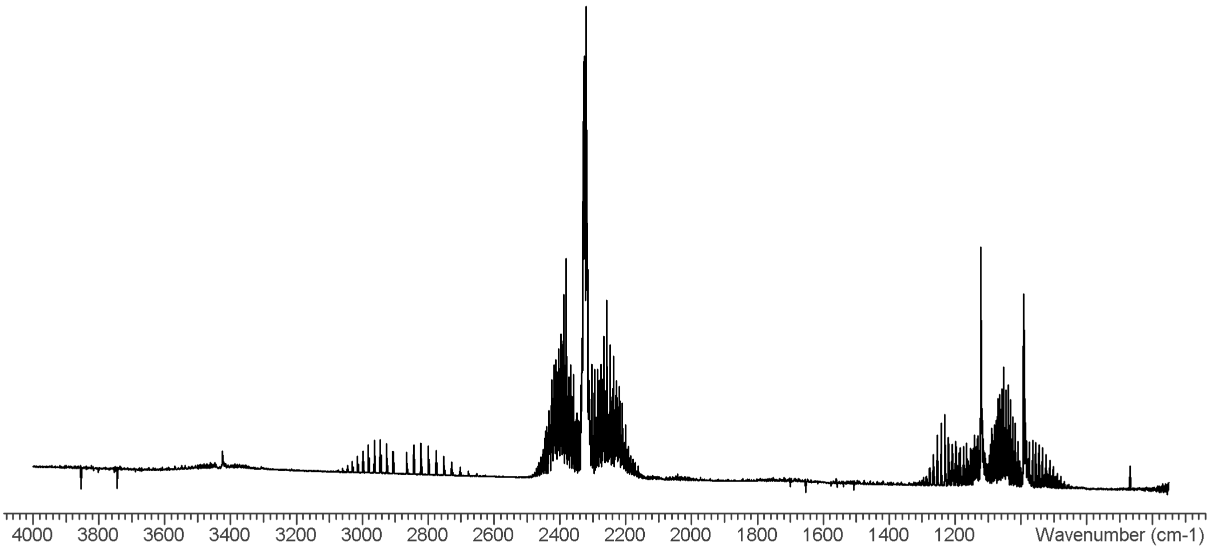

Figure S25. Raw gas-phase IR spectrum of PH_3.

1.2 [D_1]-stearic acid

Stearic acid (2100 mg, 7.34 mmol) was dissolved in THF (40 ml). To the colourless solution, Me_3SiCl (900 mg, 8.28 mmol) were added via syringe at ambient temperature. after 5 minutes of stirring, NEt_3 (1200 mg, 11.88 mmol) was added via syringe, immediately forming a colourless precipitate. After stirring overnight, all volatiles were removed in vacuo, leaving behind a colourless precipitate. To the residue 50 ml *n*-hexane were added and the mixture was filtered. The colourless solution was evaporated in vacuo, affording a colourless solid (CH_3-$(CH_2)_{16}$-C(O)$OSiMe_3$, mp. 40-45 °C) in quantitative yield.

CH_3-$(CH_2)_{16}$-C(O)$OSiMe_3$ (2610 mg, 7.32 mmol) was dissolved in THF and 1 ml of D_2O was added via syringe at ambient temperature. The mixture was stirred for three hours, then all volatiles were removed in vacuo, affording colourless [D_1]-stearic acid (CH_3-$(CH_2)_{16}$-C(O)OD) in quantitative yield.

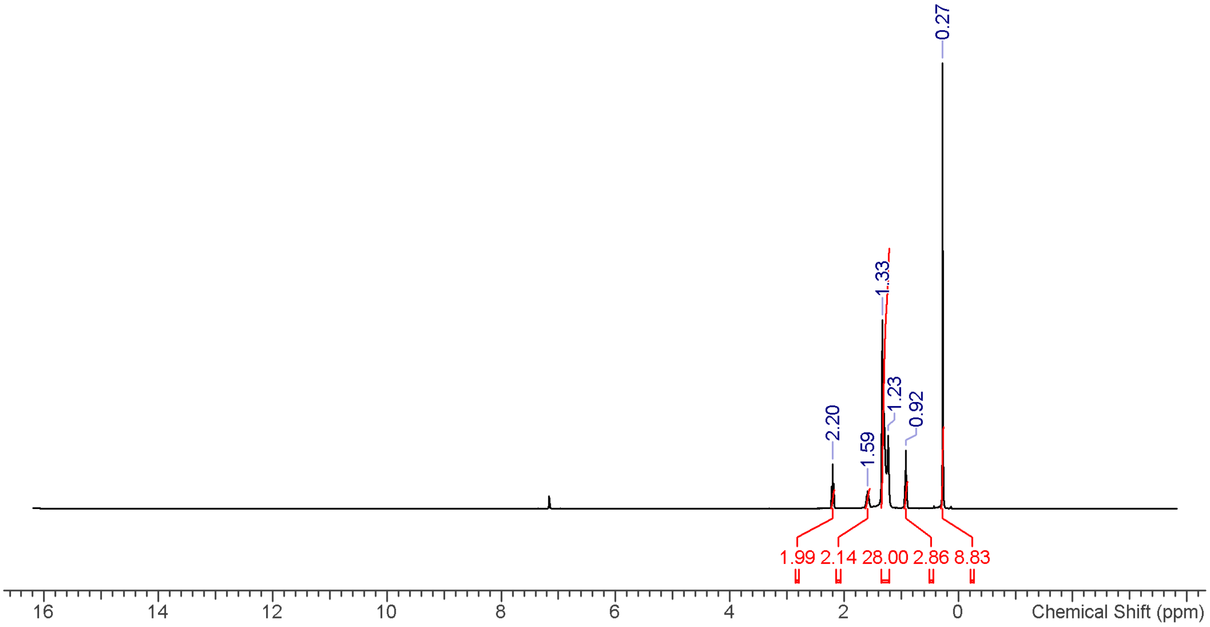

Figure S26. ^{1}H NMR spectrum of CH_3-$(CH_2)_{16}$-C(O)$OSiMe_3$ in C_6D_6 at 298 K.

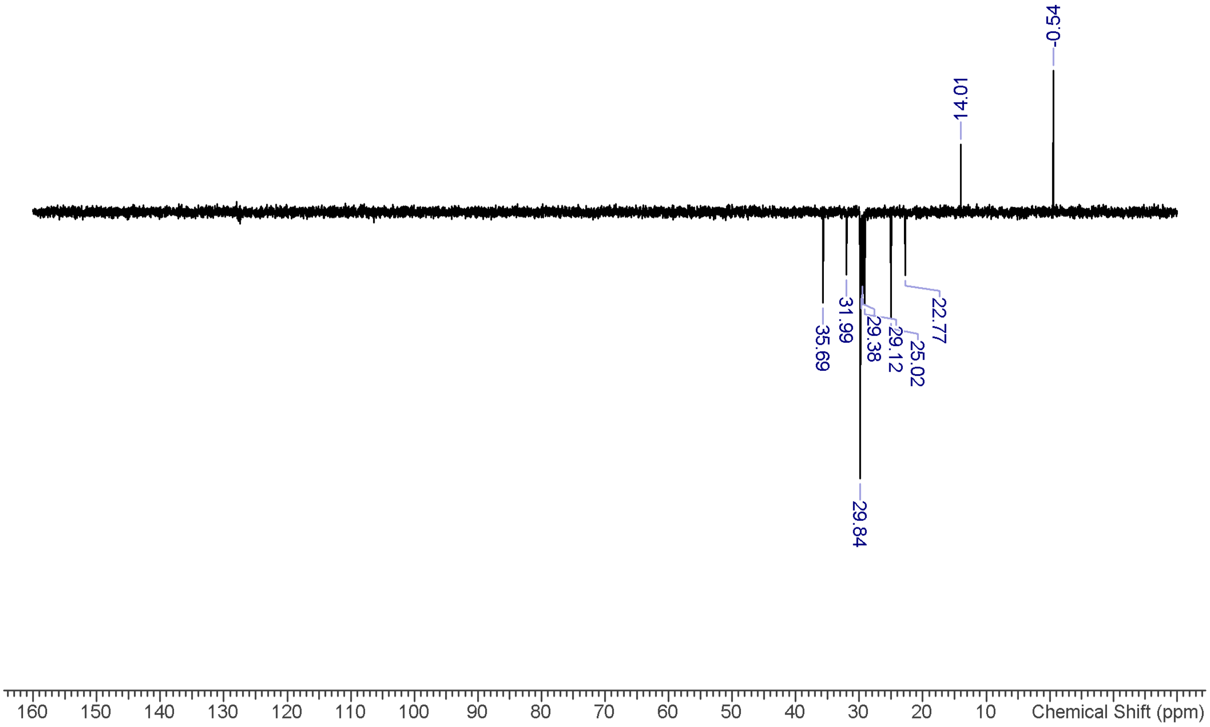

Figure S27. ^{13}C-DEPT135 NMR spectrum of CH_3-$(CH_2)_{16}$-C(O)OSiMe$_3$ in C_6D_6 at 298 K.

2. Computational data

Computational details. All computations wer performed using Gaussian09[3] utilizing the PBE1PBE level of theory and 6-311G(d,p) basis sets. No solvent corrections were applied. Natural Bond Orbital and Natural Resonance Theory were applied to study the electronic states.[4–6]

3.1 HPCO and DPCO

			Natural Population			
		Natural				
Atom	No	Charge	Core	Valence	Rydberg	Total
C	1	0.34387	1.99962	3.60027	0.05625	5.65613
P	2	0.02542	9.99822	4.94077	0.03560	14.97458
H	3	0.03328	0.00000	0.96122	0.00550	0.96672
O	4	-0.40257	1.99971	6.38248	0.02038	8.40257

```
     (Occupancy)   Bond orbital/ Coefficients
 -------------------------------------------------------------------------------
   1. (1.99461) BD ( 1) C   1 - P   2
           ( 65.63%)   0.8101* C   1 s( 61.58%)p 0.62( 38.36%)d 0.00(  0.06%)
           ( 34.37%)   0.5863* P   2 s( 16.31%)p 5.08( 82.88%)d 0.05(  0.81%)
   2. (1.99436) BD ( 2) C   1 - P   2
           ( 34.49%)   0.5873* C   1 s(  0.00%)p 1.00( 99.81%)d 0.00(  0.19%)
           ( 65.51%)   0.8094* P   2 s(  0.00%)p 1.00( 99.58%)d 0.00(  0.42%)
   3. (1.99987) BD ( 1) C   1 - O   4
           ( 32.68%)   0.5717* C   1 s( 38.58%)p 1.59( 61.27%)d 0.00(  0.15%)
           ( 67.32%)   0.8205* O   4 s( 45.88%)p 1.18( 54.00%)d 0.00(  0.12%)
   4. (1.99698) BD ( 2) C   1 - O   4
           ( 29.91%)   0.5469* C   1 s(  0.28%)p99.99( 99.19%)d 1.87(  0.53%)
           ( 70.09%)   0.8372* O   4 s(  0.83%)p99.99( 99.01%)d 0.19(  0.16%)
   5. (1.89638) BD ( 1) P   2 - H   3
           ( 49.27%)   0.7019* P   2 s( 12.17%)p 7.16( 87.16%)d 0.06(  0.67%)
           ( 50.73%)   0.7122* H   3 s( 99.74%)p 0.00(  0.26%)
  13. (1.91245) LP ( 1) P   2           s( 72.30%)p 0.38( 27.64%)d 0.00(  0.06%)

 GETTING A SIMPLE ANSWER FROM A PROFESSOR IS LIKE
 GETTING A THIMBLE OF WATER FROM A FIRE HYDRANT.
    -- PROF. LEN SHAPIRO, NDSU
```

3.1.1 HPCO – displacement vectors for the vibrations

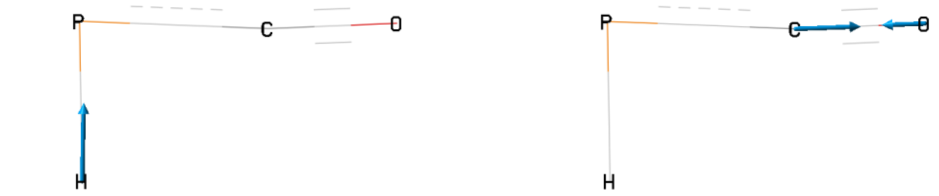

Figure S28. Displacement vectors for ν1 (left) and ν2 (right) of HPCO.

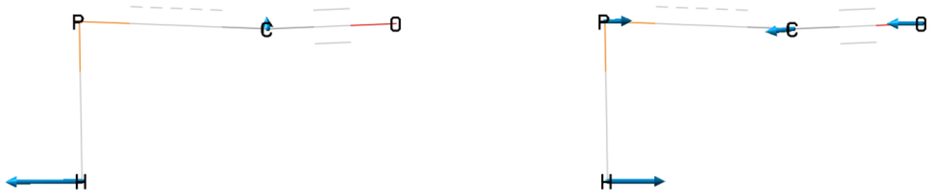

Figure S29. Displacement vectors for ν3 (left) and ν4 (right) of HPCO.

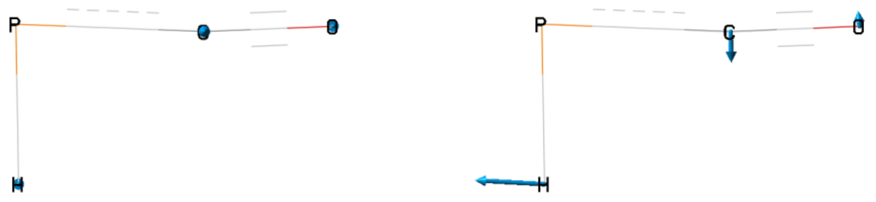

Figure S30. Displacement vectors for ν6 (left) and ν5 (right) of HPCO.

3.1.2 DPCO – displacement vectors for the vibrations

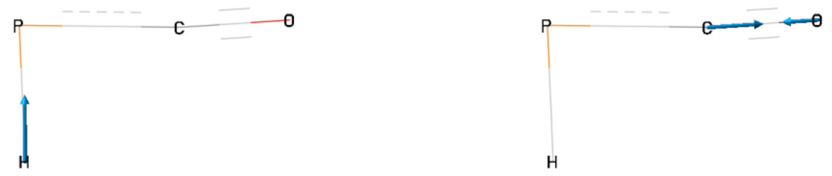

Figure S31. Displacement vectors for ν1 (left) and ν2 (right) of DPCO.

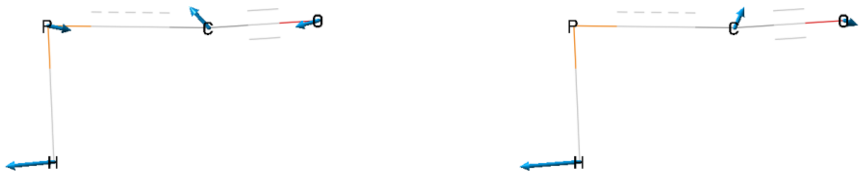

Figure S32. Displacement vectors for ν3 (left) and ν4 (right) of DPCO.

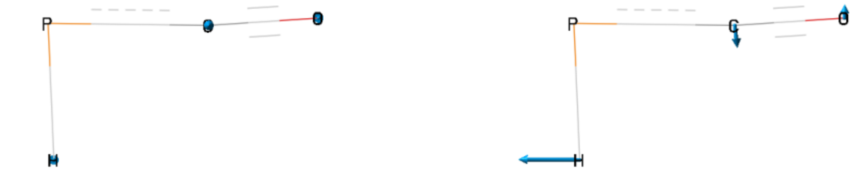

Figure S33. Displacement vectors for ν5 (left) and ν6 (right) of DPCO.

3.1.3 HPCO – vibrational analysis

===
Derivatives of the Inertia Moments w.r.t
Normal modes (in amu^1/2.Ang)
===

	Ixx	Ixy	Iyy	Ixz	Iyz	Izz
Q(1)	0.09565	0.04900	-2.81527	0.00000	0.00000	-2.71962
Q(2)	-4.46827	0.25060	-0.07077	0.00000	0.00000	-4.53904
Q(3)	2.42924	2.38620	0.15503	0.00000	0.00000	2.58427
Q(4)	-18.32119	0.42555	0.01517	0.00000	0.00000	-18.30602
Q(5)	0.67572	1.37596	-0.19553	0.00000	0.00000	0.48019
Q(6)	0.00000	0.00000	0.00000	0.00000	0.00000	0.00000

===

Vibro-rotational Alpha Matrix
==

Vibro-Rot alpha Matrix (in cm^-1)
----

	a(z)	b(x)	c(y)
Q(1)	0.24157	0.00001	0.00011
Q(2)	0.00399	0.00099	0.00094
Q(3)	-0.26752	-0.00020	0.00000
Q(4)	-0.00191	0.00073	0.00079
Q(5)	2.43152	-0.00059	-0.00029
Q(6)	-2.32447	-0.00019	-0.00037

Vibro-Rot alpha Matrix (in MHz)
----

	a	b	c
Q(1)	7242.04901	0.16560	3.15202
Q(2)	119.66048	29.53050	28.24947
Q(3)	-8020.16981	-6.08416	-0.13669
Q(4)	-57.12735	21.81956	23.66464
Q(5)	72895.05345	-17.66941	-8.63623
Q(6)	-69685.82397	-5.67142	-11.03286

==
Quartic Centrifugal Distortion Constants
==

NOTE: Values in Cartesian coords. refer to the structure in Input orientation

Quartic Centrifugal Distortion Constants Tau Prime
----

	cm^-1	MHz
TauP aaaa	-0.2003721854D-02	-0.6007006998D+02
TauP bbaa	-0.2343076287D-04	-0.7024365993D+00
TauP bbbb	-0.1839227044D-06	-0.5513863964D-02
TauP ccaa	-0.4376089400D-06	-0.1311918598D-01
TauP ccbb	-0.1758204046D-06	-0.5270963125D-02
TauP cccc	-0.1685109370D-06	-0.5051830800D-02

Asymmetric Top Reduction
Asymmetric Top Parameter Kappa : -0.9990251599
Asymmetric Top Parameter Sigma : 4102.2369680796

Constants in the Asymmetrically reduced Hamiltonian
----

	cm^-1	MHz
DELTA J :	0.4405420518D-07	0.1320711846D-02
DELTA K :	0.4950075239D-03	0.1483995223D+02
DELTA JK :	0.5878885437D-05	0.1762445516D+00
delta J :	0.9632354654D-09	0.2887707278D-04
delta K :	0.3076455124D-05	0.9222980435D-01

Effective Rotational Constants
(Include Terms due to Quartic Centrifugal Distortion Constants)

	Be in cm^-1	B(A) in cm^-1	B(A) in MHz
a	8.436553794	8.436553793	252921.51988
b	0.186258790	0.186259197	5583.91025
c	0.182235470	0.182235064	5463.26977

Nielsen Centrifugal Distortion Constants
----

	cm^-1	MHz
DJ	0.4402942917D-07	0.1319969079D-02
DJK	0.5879034093D-05	0.1762490082D+00
DK	0.4950074000D-03	0.1483994852D+02
dJ	0.9632354654D-09	0.2887707278D-04

R5 -0.1436590503D-05 -0.4306789979D-01
R6 -0.1238800440D-10 -0.3713830288D-06

Constants in the Symmetrically Reduced Hamiltonian

---

	cm^-1	MHz
D J :	0.4367923231D-07	0.1309470442D-02
D JK :	0.5881135275D-05	0.1763120000D+00
D K :	0.4950056490D-03	0.1483989602D+02
d 1 :	-0.9632354654D-09	-0.2887707278D-04
d 2 :	-0.1874864341D-09	-0.5620701892D-05

Effective Rotational Constants
(Include Terms due to Quartic Centrifugal Distortion Constants)

	Be in cm^-1	B(S) in cm^-1	B(S) in MHz
a	8.436553794	8.436553795	252921.51993
b	0.186258790	0.186253043	5583.72577
c	0.182235470	0.182241216	5463.45421

Wilson Centrifugal Distortion Constants

---

	cm^-1	MHz
DJ	0.4598067611D-07	0.1378465991D-02
DJK	0.1162342008D-04	0.3484613677D+00
DK	0.4892610628D-03	0.1466767766D+02

Nielsen Centrifugal Distortion Constants

---

	cm^-1	MHz
DJ	0.4402942917D-07	0.1319969079D-02
DJK	0.5879034093D-05	0.1762490082D+00
DK	0.4950074000D-03	0.1483994852D+02
dJ	0.9632354654D-09	0.2887707278D-04
R5	-0.1436590503D-05	-0.4306789979D-01
R6	-0.1238800440D-10	-0.3713830288D-06

Rotational Constants (in cm^-1)

---

equilibrium (e), ground vibr.state (00), and 00 + centr. dist.(0)

Ae=	8.4365538	A00=	8.3949643	A0=	8.3949643
Be=	0.1862588	B00=	0.1858904	B0=	0.1858908
Ce=	0.1822355	C00=	0.1816474	C0=	0.1816470

Rotational Constants (in MHz)

---

equilibrium (e), ground vibr.state (00), and 00 + centr. dist.(0)

Ae=	252921.520	A00=	251674.699	A0=	251674.699
Be=	5583.898	B00=	5572.853	B0=	5572.865
Ce=	5463.282	C00=	5445.652	C0=	5445.640

Test of Quartic Planarity Relationships

Asymmetric Reduction I r
4C*DELJ - (B-C)*DELJK - 2*(2A+B+C)*SDELJ+ 2*(B-C)*SDELK = 0
VALUE = 0.9595295106D-22
Symmetric Reduction I r
4C*DJ - (B-C)*DJK + 2*(2A+B+C)*D1 - 4*(4A+B-3C)*D2 = 0
Value = 0.1058791184D-21

==
Sextic Centrifugal Distortion Constants
==

Sextic Distortion Constants

```
                  in cm-1                  in Hz
Phi aaa       -0.1376284594D-05       -0.4125997414D+05
Phi aab       -0.7024130662D-07       -0.2105781396D+04
Phi aac       -0.6137600127D-08       -0.1840006228D+03
Phi abb        0.4354676859D-11        0.1305499279D+00
Phi abc       -0.3169929188D-11       -0.9503208631D-01
Phi acc       -0.6440618474D-11       -0.1930848843D+00
Phi bbb       -0.9018811379D-14       -0.2703771631D-03
Phi bbc       -0.1746360949D-13       -0.5235458416D-03
Phi bcc       -0.1992192022D-13       -0.5972441432D-03
Phi ccc       -0.1750173342D-13       -0.5246887682D-03

Asymmetric Top Reduction
------------------------
Asymmetric Top Parameter Kappa :       -0.9990251599
Asymmetric Top Parameter Sigma :     4102.2369680796

Constants in the A reduced Hamiltonian
--------------------------------------
                  in cm^-1                 in Hz
Phi J  :       -0.1326027240D-13       -0.3975329657D-03
Phi K  :       -0.1299898204D-05       -0.3896996777D+05
Phi JK :        0.1895582745D-11        0.5682814104D-01
Phi KJ :       -0.7638827272D-07       -0.2290062804D+04
phi j  :        0.2120730511D-14        0.6357790127D-04
phi k  :       -0.2111541051D-07       -0.6330240818D+03
phi jk :        0.2886598776D-11        0.8653805425D-01

rho    :       -0.1750984297D-09
mu     :       -0.3449127582D-11
nu     :       -0.1492020260D-15
lambda :        0.7430267049D-15

Constants in the S reduced Hamiltonian
--------------------------------------
                  in cm^-1                 in Hz
H J  :         -0.1370388817D-13       -0.4108322319D-03
H K  :         -0.1299883888D-05       -0.3896953858D+05
H JK :          0.8033784983D-11        0.2408468147D+00
H KJ :         -0.7640872674D-07       -0.2290676000D+04
h  1 :          0.2214209091D-14        0.6638031858D-04
h  2 :          0.2218078846D-15        0.6649633093D-05
h  3 :         -0.9347857935D-16       -0.2802417307D-05

    ==================================================
      Average Coordinates and Mean Square Amplitudes
    ==================================================

NOTE: Temperature is given in K between parentheses
      For Cartesian and internal coords., units are given between
      parentheses next to each label

Mean Square Amplitudes of Normal Coordinates (in amu.bohr^2)
------------------------------------------------------------
Mode    <Q^2> (0)    <Q^2> (298.15)    <Q^2> (298.15) class.
   1    0.025108       0.025109          0.171345
   2    0.028336       0.028338          0.218226
   3    0.068851       0.070907          1.288429
   4    0.082397       0.087394          1.845253
   5    0.155528       0.212349          6.574400
   6    0.127477       0.156563          4.416762

Average Normal Coordinates (in amu^1/2.bohr)
--------------------------------------------
Mode    <Q> (0)       <Q> (298.15)
   1   -0.021521      -0.018959
   2    0.000743       0.005735
   3    0.009742       0.009262
```

4	-0.029765	-0.026154
5	0.012782	0.011615
6	0.000000	0.000000

Mean Square Amplitudes of Cartesian Coordinates

Label	<(S-Se)^2>^1/2 (0)	<(S-Se)^2>^1/2 (298.15)
X(1) (Angs)	0.048078	0.055155
Y(1) (Angs)	0.027270	0.027633
Z(1) (Angs)	0.047216	0.052326
X(2) (Angs)	0.007495	0.008163
Y(2) (Angs)	0.018928	0.019583
Z(2) (Angs)	0.006732	0.007461
X(3) (Angs)	0.082457	0.082523
Y(3) (Angs)	0.155550	0.168259
Z(3) (Angs)	0.021531	0.023862
X(4) (Angs)	0.023403	0.027219
Y(4) (Angs)	0.026667	0.027279
Z(4) (Angs)	0.021030	0.023306

Average Cartesian Coordinates

Label	Se	<S> = Sz (0)	<S> = Sz (298.15)
X(1) (Angs)	0.000000	0.000984	0.000879
Y(1) (Angs)	0.589147	0.591220	0.591590
Z(1) (Angs)	0.000000	0.000000	0.000000
X(2) (Angs)	0.065583	0.065875	0.065847
Y(2) (Angs)	-1.091267	-1.093430	-1.093215
Z(2) (Angs)	0.000000	0.000000	0.000000
X(3) (Angs)	-1.360798	-1.372092	-1.370689
Y(3) (Angs)	-1.068099	-1.064706	-1.064737
Z(3) (Angs)	0.000000	0.000000	0.000000
X(4) (Angs)	0.047131	0.046539	0.046584
Y(4) (Angs)	1.737777	1.740198	1.739505
Z(4) (Angs)	0.000000	0.000000	0.000000

Internal coordinates for the Equilibrium structure (Se)

Interatomic distances:

		1	2	3	4
1	C	0.000000			
2	P	1.681693	0.000000		
3	H	2.144350	1.426569	0.000000	
4	O	1.149597	2.829104	3.139300	0.000000

Interatomic angles:

C1-P2-H3= 86.8345 P2-C1-O4=175.4153 H3-C1-O4=142.9596
H3-P2-O4= 88.6958

Dihedral angles:

O4-C1-P2-H3= 180.

Internal coordinates for the vibrationally average structure at 0K (Sz)

Interatomic distances:

		1	2	3	4
1	C	0.000000			
2	P	1.685900	0.000000		
3	H	2.151146	1.438254	0.000000	
4	O	1.149881	2.833694	3.143246	0.000000

Interatomic angles:

C1-P2-H3= 86.6497 P2-C1-O4=175.5236 H3-C1-O4=142.6053
H3-P2-O4= 88.4647

Dihedral angles:

O4-C1-P2-H3= 180.

Internal coordinates for the vibr. average structure at 298.15K (Sa)

Interatomic distances:

		1	2	3	4
1	C	0.000000			
2	P	1.686058	0.000000		
3	H	2.150493	1.436818	0.000000	

```
  4  O    1.148825   2.832786   3.142044   0.000000
                    Interatomic angles:
    C1-P2-H3= 86.656        P2-C1-O4=175.5117       H3-C1-O4=142.6526
    H3-P2-O4= 88.4747
                      Dihedral angles:
  O4-C1-P2-H3= 180.

  ==================================================
   Vibrational Average of Electric-Field Properties
  ==================================================

Electric dipole
---------------
----------------------------------------------------------------------
 Property at reference geometry, Unit: Debye
----------------------------------------------------------------------
   X=    -0.6671    Y=    -0.3867    Z=     0.0000
----------------------------------------------------------------------
 Temperature:     0K, Unit: Debye
----------------------------------------------------------------------
   X=    -0.6510    Y=    -0.4085    Z=     0.0000
----------------------------------------------------------------------
 Temperature:   298K, Unit: Debye
----------------------------------------------------------------------
   X=    -0.6504    Y=    -0.4075    Z=     0.0000

  ==================================================
      Nuclear Magnetic Resonance Spectroscopy
  ==================================================

Nuclear quadrupole tensors
--------------------------
----------------------------------------------------------------------
 Property at reference geometry
----------------------------------------------------------------------
          Nuclear Quadrupole in Principal Axis System
     Atom           a.u.   MegaHertz   Gauss  10(-4) cm-1        Axes
          Baa    -0.4455   -59.777   -21.330   -19.939  0.9993 -0.0367  0.0000
  1 C(13)  Bbb    -0.1044   -14.005    -4.997    -4.672  0.0000  0.0000  1.0000
          Bcc     0.5498    73.782    26.327    24.611  0.0367  0.9993  0.0000

          Baa    -1.2466  -269.488   -96.160   -89.891  0.4565  0.8897  0.0000
  2 P(31)  Bbb     0.1676    36.228    12.927    12.084  0.8897 -0.4565  0.0000
          Bcc     1.0790   233.259    83.233    77.807  0.0000  0.0000  1.0000

          Baa    -0.1838   -98.052   -34.987   -32.707  0.9999  0.0139  0.0000
  3 H(1)   Bbb     0.0784    41.811    14.919    13.946 -0.0139  0.9999  0.0000
          Bcc     0.1054    56.242    20.068    18.760  0.0000  0.0000  1.0000

          Baa    -0.5889    42.613    15.205    14.214  0.9999  0.0110  0.0000
  4 O(17)  Bbb    -0.0412     2.980     1.063     0.994 -0.0110  0.9999  0.0000
          Bcc     0.6301   -45.592   -16.268   -15.208  0.0000  0.0000  1.0000

----------------------------------------------------------------------
 Temperature:     0K
----------------------------------------------------------------------
          Nuclear Quadrupole in Principal Axis System
     Atom           a.u.   MegaHertz   Gauss  10(-4) cm-1        Axes
          Baa    -0.4408   -59.146   -21.105   -19.729  0.9994 -0.0336 -0.0001
  1 C(13)  Bbb    -0.1020   -13.682    -4.882    -4.564  0.0001  0.0000  1.0000
          Bcc     0.5427    72.828    25.987    24.293  0.0336  0.9994  0.0000

          Baa    -1.2129  -262.217   -93.566   -87.466  0.4641  0.8858  0.0000
  2 P(31)  Bbb     0.1167    25.224     9.001     8.414  0.8858 -0.4641  0.0000
          Bcc     1.0963   236.993    84.565    79.052  0.0000  0.0000  1.0000

          Baa    -0.1771   -94.516   -33.726   -31.527  0.9999  0.0113  0.0000
  3 H(1)   Bbb     0.0741    39.527    14.104    13.185 -0.0113  0.9999  0.0000
```

```
          Bcc     0.1031    54.989    19.621    18.342  0.0000  0.0000  1.0000

          Baa    -0.5810    42.039    15.000    14.023  0.9999  0.0134 -0.0001
   4 O(17)  Bbb    -0.0455     3.293     1.175     1.098 -0.0134  0.9999  0.0000
          Bcc     0.6265   -45.331   -16.175   -15.121  0.0001  0.0000  1.0000

 ------------------------------------------------------------------------
 Temperature:   298K
 ------------------------------------------------------------------------
          Nuclear Quadrupole in Principal Axis System
     Atom            a.u.   MegaHertz   Gauss  10(-4) cm-1       Axes
          Baa    -0.4399   -59.027   -21.062   -19.689  0.9994 -0.0336 -0.0001
   1 C(13)  Bbb    -0.1018   -13.665    -4.876    -4.558  0.0001  0.0000  1.0000
          Bcc     0.5417    72.692    25.938    24.247  0.0336  0.9994  0.0000

          Baa    -1.2081  -261.177   -93.194   -87.119  0.4632  0.8862  0.0000
   2 P(31)  Bbb     0.1135    24.541     8.757     8.186  0.8862 -0.4632  0.0000
          Bcc     1.0946   236.636    84.438    78.933  0.0000  0.0000  1.0000

          Baa    -0.1765   -94.181   -33.606   -31.415  0.9999  0.0117  0.0000
   3 H(1)   Bbb     0.0735    39.222    13.995    13.083 -0.0117  0.9999  0.0000
          Bcc     0.1030    54.959    19.611    18.332  0.0000  0.0000  1.0000

          Baa    -0.5823    42.135    15.035    14.055  0.9999  0.0121 -0.0001
   4 O(17)  Bbb    -0.0414     2.999     1.070     1.000 -0.0121  0.9999  0.0000
          Bcc     0.6237   -45.134   -16.105   -15.055  0.0001  0.0000  1.0000


    ==================================================
              Resonance Analysis
    ==================================================

Thresholds
----------
Maximum Difference PT2 vs. Variational (cm-1) :       1.000
Minimum value for Darling-Dennison term (cm-1):      10.000

Fermi resonances
----------------
 No Fermi resonance found

Darling-Dennison resonances
---------------------------
 No Darling-Dennison resonance found

    ==================================================
              Anharmonic X Matrix
    ==================================================

PT2 model: Deperturbed VPT2 (DVPT2)
Ref.: V. Barone, J. Chem. Phys. 122, 1, 014108 (2005)

Coriolis contributions to X Matrix (in cm^-1)
---------------------------------------------
             1             2             3             4             5
    1  0.000000D+00
    2  0.124897D-04  0.000000D+00
    3  0.397471D+00  0.114554D+00  0.000000D+00
    4  0.115801D-01  0.110884D-02  0.213853D-01  0.000000D+00
    5  0.301412D+00  0.745306D+00  0.468391D-03  0.749382D-02  0.000000D+00
    6  0.346380D+00  0.863986D+00  0.489361D+01  0.558797D-01  0.128551D+02
             6
    6  0.000000D+00

3rd Deriv. contributions to X Matrix (in cm^-1)
-----------------------------------------------
             1             2             3             4             5
    1 -0.905858D+02
```

```
    2 -0.123109D+01 -0.232407D+02
    3  0.470393D+02  0.164388D+01 -0.585000D+01
    4 -0.319201D+00 -0.474270D+01 -0.387102D+00 -0.600270D+01
    5  0.548201D+02  0.304157D+02 -0.302015D+02 -0.122967D+02 -0.122119D+02
    6  0.683705D+00  0.295755D+02 -0.301145D+00 -0.147659D+01 -0.111515D+02
              6
    6 -0.816466D+01

4th Deriv. contributions to X Matrix (in cm^-1)
-----------------------------------------------
              1             2             3             4             5
    1  0.717822D+02
    2  0.813928D-01  0.120578D+02
    3 -0.606904D+02 -0.496199D+01 -0.308535D+00
    4 -0.120983D+01  0.374971D+01 -0.262897D+01  0.194679D+01
    5 -0.507975D+02 -0.350812D+02  0.192249D+02 -0.295369D+01  0.154440D+02
    6 -0.122383D+01 -0.360091D+02 -0.226817D+01 -0.368478D+01  0.122043D+02
              6
    6  0.894709D+01

Total Anharmonic X Matrix (in cm^-1)
------------------------------------
              1             2             3             4             5
    1 -0.188036D+02
    2 -0.114968D+01 -0.111830D+02
    3 -0.132536D+02 -0.320356D+01 -0.615854D+01
    4 -0.151745D+01 -0.991885D+00 -0.299468D+01 -0.405591D+01
    5  0.432407D+01 -0.392021D+01 -0.109761D+02 -0.152429D+02  0.323207D+01
    6 -0.193744D+00 -0.556957D+01  0.232429D+01 -0.510550D+01  0.139080D+02
              6
    6  0.782427D+00

    ==================================================
            Anharmonic Zero Point Energy
    ==================================================

Anharmonic Zero Point Energy
----------------------------
Harmonic        : cm-1 =  3493.18738 ; Kcal/mol =   9.988 ; KJ/mol =  41.788
Anharm.Pot.     : cm-1 =   -17.70850 ; Kcal/mol =  -0.051 ; KJ/mol =  -0.212
Watson+Coriolis: cm-1 =    -1.54097 ; Kcal/mol =  -0.004 ; KJ/mol =  -0.018
X0F (Im. Freq.): cm-1 =     0.00000 ; Kcal/mol =   0.000 ; KJ/mol =   0.000
Total Anharm    : cm-1 =  3473.93791 ; Kcal/mol =   9.932 ; KJ/mol =  41.558

    ==================================================
         Vibrational Energies at Anharmonic Level
    ==================================================

Vibrational Energies and Rotational Constants (in cm^-1)
--------------------------------------------------------
Mode(Quanta)      E(harm)     E(anharm)     Aa(z)       Ba(x)       Ca(y)
Equilibrium Geometry                       8.436554    0.186259    0.182235
Ground State       3493.187    3473.938    8.394964    0.185891    0.181647
Fundamental Bands (DE w.r.t. Ground State)
  1(1) active      2397.602    2354.100    8.153396    0.185885    0.181542
  2(1) active      2124.515    2094.732    8.390973    0.184906    0.180705
  3(1) active       874.345     847.976    8.662488    0.186094    0.181652
  4(1) active       730.609     709.571    8.396870    0.185163    0.180858
  5(1) active       387.066     387.576    5.963447    0.186480    0.181935
  6(1) active       472.238     476.485   10.719433    0.186080    0.182015
Overtones (DE w.r.t. Ground State)
  1(2)             4795.204    4670.592    7.911827    0.185880    0.181437
  2(2)             4249.031    4167.098    8.386981    0.183921    0.179762
  3(2)             1748.689    1683.634    8.930012    0.186297    0.181656
  4(2)             1461.218    1411.030    8.398775    0.184435    0.180068
  5(2)              774.132     781.617    3.531930    0.187070    0.182223
  6(2)              944.476     954.534   13.043902    0.186269    0.182383
Combination Bands (DE w.r.t. Ground State)
```

2(1)	1(1)	4522.117	4447.682	8.149404	0.184900	0.180600
3(1)	1(1)	3271.947	3188.822	8.420920	0.186088	0.181546
3(1)	2(1)	2998.860	2939.504	8.658497	0.185109	0.180709
4(1)	1(1)	3128.211	3062.153	8.155301	0.185157	0.180752
4(1)	2(1)	2855.124	2803.311	8.392878	0.184178	0.179915
4(1)	3(1)	1604.953	1554.552	8.664394	0.185366	0.180862
5(1)	1(1)	2784.668	2746.000	5.721878	0.186475	0.181830
5(1)	2(1)	2511.581	2478.388	5.959456	0.185495	0.180993
5(1)	3(1)	1261.410	1224.576	6.230971	0.186683	0.181940
5(1)	4(1)	1117.675	1081.904	5.965353	0.185752	0.181146
6(1)	1(1)	2869.840	2830.390	10.477864	0.186074	0.181910
6(1)	2(1)	2596.753	2565.647	10.715442	0.185095	0.181073
6(1)	3(1)	1346.583	1326.785	10.986957	0.186283	0.182020
6(1)	4(1)	1202.847	1180.950	10.721339	0.185352	0.181226
6(1)	5(1)	859.304	877.969	8.287916	0.186669	0.182303

==

Thermodynamic properties and Partition Functions

==

ZPE(harm) = 0.41788D+02 kJ/mol   ZPE(anh)= 0.41558D+02 kJ/mol

Input values of T(K) and P(atm): 298.15   1.00

	Harmonic value	SPT anharmonic value	
Qvib	0.65813D-07	0.72402D-07	
QZvib	0.13779D+01	0.13813D+01	
Energy	0.51137D+02	0.50934D+02	kJ/mol
Enthalpy	0.53616D+02	0.53413D+02	kJ/mol
Entropy	0.25784D+03	0.25795D+03	J/(mol K)
Sp.Heat(V)	0.42211D+02	0.42500D+02	J/(mol K)
Sp.Heat(P)	0.50526D+02	0.50814D+02	J/(mol K)

T = 298.15 K; P =   1.00 atm

	Harmonic value	SPT anharmonic value	
Qvib	0.65813D-07	0.72402D-07	
QZvib	0.13779D+01	0.13813D+01	
Energy	0.51137D+02	0.50934D+02	kJ/mol
Enthalpy	0.53616D+02	0.53413D+02	kJ/mol
Entropy	0.25784D+03	0.25795D+03	J/(mol K)
Sp.Heat(V)	0.42211D+02	0.42500D+02	J/(mol K)
Sp.Heat(P)	0.50526D+02	0.50814D+02	J/(mol K)

T = 500.00 K; P =   1.00 atm

	Harmonic value	SPT anharmonic value	
Qvib	0.10738D-03	0.11476D-03	
QZvib	0.24910D+01	0.25188D+01	
Energy	0.60697D+02	0.60557D+02	kJ/mol
Enthalpy	0.64854D+02	0.64714D+02	kJ/mol
Entropy	0.28641D+03	0.28669D+03	J/(mol K)
Sp.Heat(V)	0.51543D+02	0.51861D+02	J/(mol K)
Sp.Heat(P)	0.59857D+02	0.60175D+02	J/(mol K)

T = 1000.00 K; P =   1.00 atm

	Harmonic value	SPT anharmonic value	
Qvib	0.72582D-01	0.76830D-01	
QZvib	0.11055D+02	0.11382D+02	
Energy	0.89797D+02	0.89808D+02	kJ/mol
Enthalpy	0.98111D+02	0.98122D+02	kJ/mol
Entropy	0.33205D+03	0.33253D+03	J/(mol K)
Sp.Heat(V)	0.63313D+02	0.63582D+02	J/(mol K)
Sp.Heat(P)	0.71628D+02	0.71896D+02	J/(mol K)

T = 1500.00 K; P =   1.00 atm

	Harmonic value	SPT anharmonic value	
Qvib	0.13868D+01	0.14717D+01	
QZvib	0.39553D+02	0.41205D+02	
Energy	0.12294D+03	0.12306D+03	kJ/mol
Enthalpy	0.13541D+03	0.13553D+03	kJ/mol

Entropy	0.36222D+03	0.36280D+03	J/(mol K)
Sp.Heat(V)	0.68560D+02	0.68737D+02	J/(mol K)
Sp.Heat(P)	0.76874D+02	0.77051D+02	J/(mol K)

T = 2000.00 K; P = 1.00 atm

	Harmonic value	SPT anharmonic value	
Qvib	0.94238D+01	0.10032D+02	
QZvib	0.11630D+03	0.12211D+03	
Energy	0.15790D+03	0.15810D+03	kJ/mol
Enthalpy	0.17453D+03	0.17472D+03	kJ/mol
Entropy	0.38471D+03	0.38533D+03	J/(mol K)
Sp.Heat(V)	0.71007D+02	0.71123D+02	J/(mol K)
Sp.Heat(P)	0.79321D+02	0.79438D+02	J/(mol K)

==

Anharmonic Transition Moments

==

Electric dipole : Fundamental Bands

Mode(Quanta)	X	Y	Z
1(1)	-0.202030E-01	-0.521957E-02	0.121075E-05
2(1)	0.506949E-02	0.141696	-0.875508E-06
3(1)	0.254319E-01	-0.147616E-01	-0.987147E-06
4(1)	0.230256E-02	-0.203568E-01	0.705510E-13
5(1)	0.330765E-01	0.128453E-01	-0.292233E-05
6(1)	0.154851E-12	-0.131370E-05	0.316247E-02

Electric dipole : Overtones

Mode(Quanta)	X	Y	Z
1(2)	-0.194582E-03	0.310704E-03	0.169556E-13
2(2)	0.150599E-03	0.934808E-02	-0.447082E-14
3(2)	-0.304696E-02	-0.431908E-02	0.176215E-13
4(2)	-0.305587E-03	0.423701E-02	-0.187956E-14
5(2)	-0.573122E-03	0.116518E-01	-0.259840E-13
6(2)	0.861630E-03	0.650419E-02	-0.115270E-13

Electric dipole : Combination Bands

Mode(Quanta)		X	Y	Z
2(1)	1(1)	-0.237073E-02	-0.670918E-04	0.675588E-15
3(1)	1(1)	0.238137E-03	-0.657890E-03	-0.504806E-14
3(1)	2(1)	0.389841E-03	-0.256330E-02	0.316042E-15
4(1)	1(1)	0.175716E-02	-0.873543E-03	-0.562337E-15
4(1)	2(1)	0.788197E-03	0.102210E-01	-0.735385E-14
4(1)	3(1)	-0.180804E-03	-0.291041E-02	0.278977E-14
5(1)	1(1)	0.103888E-02	0.141674E-02	-0.625682E-14
5(1)	2(1)	0.838431E-03	0.804261E-05	-0.338008E-15
5(1)	3(1)	-0.333575E-02	-0.119550E-01	0.242904E-13
5(1)	4(1)	-0.109541E-02	-0.131268E-02	0.294296E-14
6(1)	1(1)	0.786269E-17	-0.110649E-15	-0.539258E-03
6(1)	2(1)	-0.228913E-14	0.322140E-13	0.242393E-03
6(1)	3(1)	0.754280E-16	-0.106147E-14	-0.154994E-02
6(1)	4(1)	-0.232129E-14	0.326666E-13	-0.390013E-03
6(1)	5(1)	-0.670123E-15	0.943038E-14	0.131145E-04

ERROR: Missing derivatives of the polarizability tensor moment.

==

Anharmonic Infrared Spectroscopy

==

Units: Energies (E) in cm^-1

Integrated intensity (I) in km.mol^-1

Fundamental Bands

Mode(Quanta)	E(harm)	E(anharm)	I(harm)	I(anharm)

1(1)	2397.602	2354.100	15.64554092	16.59877824
2(1)	2124.515	2094.732	706.83958695	681.95255009
3(1)	874.345	847.976	12.43306229	11.87406086
4(1)	730.609	709.571	6.20967207	4.82271436
5(1)	387.066	387.576	8.20547358	7.90239049
6(1)	472.238	476.485	0.08407113	0.07717163

Overtones

Mode(Quanta)	E(harm)	E(anharm)	I(anharm)
1(2)	4795.204	4670.592	0.01016545
2(2)	4249.031	4167.098	5.89859610
3(2)	1748.689	1683.634	0.76174120
4(2)	1461.218	1411.030	0.41234971
5(2)	774.132	781.617	1.72259843
6(2)	944.476	954.534	0.66541041

Combination Bands

Mode(Quanta)		E(harm)	E(anharm)	I(anharm)
2(1)	1(1)	4522.117	4447.682	0.40513905
3(1)	1(1)	3271.947	3188.822	0.02527932
3(1)	2(1)	2998.860	2939.504	0.32000899
4(1)	1(1)	3128.211	3062.153	0.19095180
4(1)	2(1)	2855.124	2803.311	4.77077948
4(1)	3(1)	1604.953	1554.552	0.21406353
5(1)	1(1)	2784.668	2746.000	0.13724955
5(1)	2(1)	2511.581	2478.388	0.02821639
5(1)	3(1)	1261.410	1224.576	3.05494818
5(1)	4(1)	1117.675	1081.904	0.05121325
6(1)	1(1)	2869.840	2830.390	0.01332897
6(1)	2(1)	2596.753	2565.647	0.00244115
6(1)	3(1)	1346.583	1326.785	0.05161650
6(1)	4(1)	1202.847	1180.950	0.00290902
6(1)	5(1)	859.304	877.969	0.00000245

3.1.4 DPCO – vibrational analysis

===
Derivatives of the Inertia Moments w.r.t
Normal modes (in amu^1/2.Ang)
===

	Ixx	Ixy	Iyy	Ixz	Iyz	Izz
Q(1)	-4.46959	0.25011	0.04059	0.00000	0.00000	-4.42900
Q(2)	-0.06016	0.06569	-3.92373	0.00000	0.00000	-3.98389
Q(3)	14.37964	1.58234	0.05935	0.00000	0.00000	14.43899
Q(4)	-11.78577	2.30525	0.07161	0.00000	0.00000	-11.71416
Q(5)	-0.83425	2.65938	-0.12836	0.00000	0.00000	-0.96261
Q(6)	0.00000	0.00000	0.00000	0.00000	0.00000	0.00000

===
Vibro-rotational Alpha Matrix
===

Vibro-Rot alpha Matrix (in cm^-1)
----

	a(z)	b(x)	c(y)
Q(1)	0.00157	0.00097	0.00090
Q(2)	0.09114	-0.00003	0.00010
Q(3)	-0.05573	0.00054	0.00045
Q(4)	-0.12253	-0.00003	0.00030
Q(5)	0.27478	-0.00064	-0.00022
Q(6)	-0.15894	-0.00019	-0.00035

Vibro-Rot alpha Matrix (in MHz)

----

	a	b	c
Q(1)	47.12532	29.15029	26.87539
Q(2)	2732.16628	-0.90877	3.05857
Q(3)	-1670.79146	16.07469	13.60570
Q(4)	-3673.30256	-0.78327	9.13977
Q(5)	8237.76049	-19.31603	-6.57153
Q(6)	-4764.99421	-5.78791	-10.58410

==

Quartic Centrifugal Distortion Constants

==

NOTE: Values in Cartesian coords. refer to the structure in Input orientation

Quartic Centrifugal Distortion Constants Tau Prime

----

	cm^-1	MHz
TauP aaaa	-0.4661373702D-03	-0.1397444680D+02
TauP bbaa	-0.2322547432D-04	-0.6962822036D+00
TauP bbbb	-0.1815874715D-06	-0.5443855443D-02
TauP ccaa	-0.8390622327D-06	-0.2515445292D-01
TauP ccbb	-0.1673826811D-06	-0.5018006538D-02
TauP cccc	-0.1555209815D-06	-0.4662401732D-02

Asymmetric Top Reduction
Asymmetric Top Parameter Kappa : -0.9964695461
Asymmetric Top Parameter Sigma : 1131.9987773354

Constants in the Asymmetrically reduced Hamiltonian

----

	cm^-1	MHz
DELTA J :	0.4213855663D-07	0.1263282147D-02
DELTA K :	0.1105606399D-03	0.3314524598D+01
DELTA JK :	0.5931564140D-05	0.1778238193D+00
delta J :	0.1629155625D-08	0.4884085693D-04
delta K :	0.2962445860D-05	0.8881189262D-01

Effective Rotational Constants
(Include Terms due to Quartic Centrifugal Distortion Constants)

	Be in cm^-1	B(A) in cm^-1	B(A) in MHz
a	4.376534287	4.376534287	131205.19713
b	0.183878356	0.183878688	5512.54438
c	0.176464279	0.176463948	5290.25607

Nielsen Centrifugal Distortion Constants

----

	cm^-1	MHz
DJ	0.4206533504D-07	0.1261087019D-02
DJK	0.5932003469D-05	0.1778369901D+00
DK	0.1105602738D-03	0.3314513622D+01
dJ	0.1629155625D-08	0.4884085693D-04
R5	-0.1398336178D-05	-0.4192106399D-01
R6	-0.3661079588D-10	-0.1097564049D-05

Constants in the Symmetrically Reduced Hamiltonian

----

	cm^-1	MHz
D J :	0.4083005440D-07	0.1224054237D-02
D JK :	0.5939415153D-05	0.1780591868D+00
D K :	0.1105540973D-03	0.3314328459D+01
d 1 :	-0.1629155625D-08	-0.4884085693D-04
d 2 :	-0.6542511175D-09	-0.1961395507D-04

Effective Rotational Constants

(Include Terms due to Quartic Centrifugal Distortion Constants)

	Be in cm^-1	B(S) in cm^-1	B(S) in MHz
a	4.376534287	4.376534293	131205.19733
b	0.183878356	0.183872760	5512.36668
c	0.176464279	0.176469870	5290.43362

Wilson Centrifugal Distortion Constants
----

	cm^-1	MHz
DJ	0.4539686788D-07	0.1360963861D-02
DJK	0.1152194343D-04	0.3454191741D+00
DK	0.1049670023D-03	0.3146831562D+01

Nielsen Centrifugal Distortion Constants
----

	cm^-1	MHz
DJ	0.4206533504D-07	0.1261087019D-02
DJK	0.5932003469D-05	0.1778369901D+00
DK	0.1105602738D-03	0.3314513622D+01
dJ	0.1629155625D-08	0.4884085693D-04
R5	-0.1398336178D-05	-0.4192106399D-01
R6	-0.3661079588D-10	-0.1097564049D-05

Rotational Constants (in cm^-1)
----

equilibrium (e), ground vibr.state (00), and 00 + centr. dist.(0)

Ae=	4.3765343	A00=	4.3613911	A0=	4.3613911
Be=	0.1838784	B00=	0.1835710	B0=	0.1835713
Ce=	0.1764643	C00=	0.1758718	C0=	0.1758715

Rotational Constants (in MHz)
----

equilibrium (e), ground vibr.state (00), and 00 + centr. dist.(0)

Ae=	131205.197	A00=	130751.215	A0=	130751.215
Be=	5512.534	B00=	5503.320	B0=	5503.330
Ce=	5290.266	C00=	5272.504	C0=	5272.494

Test of Quartic Planarity Relationships

Asymmetric Reduction I r

4C*DELJ - (B-C)*DELJK - 2*(2A+B+C)*SDELJ+ 2*(B-C)*SDELK = 0

VALUE = -0.5293955920D-22

Symmetric Reduction I r

4C*DJ - (B-C)*DJK + 2*(2A+B+C)*D1 - 4*(4A+B-3C)*D2 = 0

Value = -0.5293955920D-22

==

Sextic Centrifugal Distortion Constants

==

Sextic Distortion Constants
----

	in cm-1	in Hz
Phi aaa	-0.6273608711D-08	-0.1880780576D+03
Phi aab	-0.1511046531D-07	-0.4530003536D+03
Phi aac	-0.3094483438D-08	-0.9277027961D+02
Phi abb	0.2027863845D-10	0.6079382866D+00
Phi abc	0.1397119605D-10	0.4188459204D+00
Phi acc	-0.2082873282D-11	-0.6244297010D-01
Phi bbb	-0.8035701647D-14	-0.2409042749D-03
Phi bbc	-0.2293178358D-13	-0.6874775767D-03
Phi bcc	-0.1679987751D-13	-0.5036476573D-03
Phi ccc	-0.1403052349D-13	-0.4206245123D-03

Asymmetric Top Reduction

Asymmetric Top Parameter Kappa : -0.9964695461
Asymmetric Top Parameter Sigma : 1131.9987773354

Constants in the A reduced Hamiltonian
----

	in cm^-1	in Hz
Phi J :	-0.1103311257D-13	-0.3307643936D-03
Phi K :	0.1196404184D-07	0.3586729510D+03
Phi JK :	0.2383465618D-10	0.7145450161D+00
Phi KJ :	-0.1826147417D-07	-0.5474652228D+03
phi j :	0.1498705460D-14	0.4493005936D-04
phi k :	-0.1238561333D-08	-0.3713113463D+02
phi jk :	0.1314104718D-10	0.3939586834D+00

rho : -0.6176403217D-09
mu : -0.1791811881D-11
nu : -0.2141235974D-15
lambda : 0.4274539604D-14

Constants in the S reduced Hamiltonian
----

	in cm^-1	in Hz
H J :	-0.1613669259D-13	-0.4837658737D-03
H K :	0.1196103420D-07	0.3585827844D+03
H JK :	0.2257629223D-10	0.6768202140D+00
H KJ :	-0.1825720307D-07	-0.5473371785D+03
h 1 :	0.1427537901D-14	0.4279650963D-04
h 2 :	0.2551790014D-14	0.7650074006D-04
h 3 :	0.7116755858D-16	0.2133549732D-05

==
Average Coordinates and Mean Square Amplitudes
==

NOTE: Temperature is given in K between parentheses
For Cartesian and internal coords., units are given between
parentheses next to each label

Mean Square Amplitudes of Normal Coordinates (in amu.bohr^2)

Mode	<Q^2> (0)	<Q^2> (298.15)	<Q^2> (298.15) class.
1	0.028333	0.028335	0.218191
2	0.034982	0.034999	0.332599
3	0.079078	0.083197	1.699610
4	0.088817	0.095829	2.144020
5	0.174435	0.255798	8.269954
6	0.127896	0.157320	4.445781

Average Normal Coordinates (in amu^1/2.bohr)

Mode	<Q> (0)	<Q> (298.15)
1	0.002011	0.007234
2	-0.021884	-0.017129
3	0.026563	0.023376
4	-0.018183	-0.015622
5	0.008896	0.007599
6	0.000000	0.000000

Mean Square Amplitudes of Cartesian Coordinates

Label	<(S-Se)^2>^1/2 (0)	<(S-Se)^2>^1/2 (298.15)
X(1) (Angs)	0.047429	0.054619
Y(1) (Angs)	0.027523	0.027981
Z(1) (Angs)	0.047449	0.052625
X(2) (Angs)	0.008256	0.008606
Y(2) (Angs)	0.019254	0.020064
Z(2) (Angs)	0.006765	0.007503
X(3) (Angs)	0.067890	0.067950

```
Y(3)  (Angs)       0.128577              0.148095
Z(3)  (Angs)       0.010827              0.012008
X(4)  (Angs)       0.024740              0.029553
Y(4)  (Angs)       0.026864              0.027504
Z(4)  (Angs)       0.021134              0.023439
```

Average Cartesian Coordinates

```
-----------------------------
   Label           Se        <S> = Sz (0)   <S> = Sz (298.15)
X(1)  (Angs)      0.000000       0.000772       0.000648
Y(1)  (Angs)      0.589147       0.591327       0.591718
Z(1)  (Angs)      0.000000       0.000000       0.000000
X(2)  (Angs)      0.065583       0.066046       0.065953
Y(2)  (Angs)     -1.091267      -1.093446      -1.093202
Z(2)  (Angs)      0.000000       0.000000       0.000000
X(3)  (Angs)     -1.360798      -1.368797      -1.367085
Y(3)  (Angs)     -1.068099      -1.066842      -1.066961
Z(3)  (Angs)      0.000000       0.000000       0.000000
X(4)  (Angs)      0.047131       0.046661       0.046721
Y(4)  (Angs)      1.737777       1.740202       1.739452
Z(4)  (Angs)      0.000000       0.000000       0.000000
```

Internal coordinates for the Equilibrium structure (Se)

```
                 Interatomic distances:
             1         2         3         4
   1  C    0.000000
   2  P    1.681693   0.000000
   3  H    2.144350   1.426569   0.000000
   4  O    1.149597   2.829104   3.139300   0.000000
                 Interatomic angles:
     C1-P2-H3= 86.8345       P2-C1-O4=175.4153       H3-C1-O4=142.9596
     H3-P2-O4= 88.6958
                   Dihedral angles:
   O4-C1-P2-H3= 180.
```

Internal coordinates for the vibrationally average structure at 0K (Sz)

```
                 Interatomic distances:
             1         2         3         4
   1  C    0.000000
   2  P    1.686037   0.000000
   3  H    2.150638   1.435090   0.000000
   4  O    1.149791   2.833715   3.143727   0.000000
                 Interatomic angles:
     C1-P2-H3= 86.7191       P2-C1-O4=175.494       H3-C1-O4=142.7322
     H3-P2-O4= 88.5458
                   Dihedral angles:
   O4-C1-P2-H3= 180.
```

Internal coordinates for the vibr. average structure at 298.15K (Sa)

```
                 Interatomic distances:
             1         2         3         4
   1  C    0.000000
   2  P    1.686185   0.000000
   3  H    2.149863   1.433278   0.000000
   4  O    1.148659   2.832719   3.142420   0.000000
                 Interatomic angles:
     C1-P2-H3= 86.7314       P2-C1-O4=175.4817       H3-C1-O4=142.79
     H3-P2-O4= 88.562
                   Dihedral angles:
   O4-C1-P2-H3= 180.
```

```
    ====================================================
     Vibrational Average of Electric-Field Properties
    ====================================================
```

Electric dipole

```
---------------
----------------------------------------------------------------------
```

```
 Property at reference geometry, Unit: Debye
 ------------------------------------------------------------------------
   X=    -0.6671    Y=    -0.3867    Z=     0.0000
 ------------------------------------------------------------------------
 Temperature:     0K, Unit: Debye
 ------------------------------------------------------------------------
   X=    -0.6548    Y=    -0.4050    Z=     0.0000
 ------------------------------------------------------------------------
 Temperature:   298K, Unit: Debye
 ------------------------------------------------------------------------
   X=    -0.6546    Y=    -0.4050    Z=     0.0000

    ====================================================
        Nuclear Magnetic Resonance Spectroscopy
    ====================================================

Nuclear quadrupole tensors
--------------------------
------------------------------------------------------------------------
 Property at reference geometry
------------------------------------------------------------------------
          Nuclear Quadrupole in Principal Axis System
     Atom            a.u.   MegaHertz   Gauss  10(-4) cm-1        Axes
          Baa    -0.4455   -59.777   -21.330   -19.939  0.9993 -0.0367  0.0000
   1 C(13)  Bbb    -0.1044   -14.005    -4.997    -4.672  0.0000  0.0000  1.0000
          Bcc     0.5498    73.782    26.327    24.611  0.0367  0.9993  0.0000

          Baa    -1.2466  -269.488   -96.160   -89.891  0.4565  0.8897  0.0000
   2 P(31)  Bbb     0.1676    36.228    12.927    12.084  0.8897 -0.4565  0.0000
          Bcc     1.0790   233.259    83.233    77.807  0.0000  0.0000  1.0000

          Baa    -0.1838   -15.052    -5.371    -5.021  0.9999  0.0139  0.0000
   3 H(2)   Bbb     0.0784     6.418     2.290     2.141 -0.0139  0.9999  0.0000
          Bcc     0.1054     8.633     3.081     2.880  0.0000  0.0000  1.0000

          Baa    -0.5889    42.613    15.205    14.214  0.9999  0.0110  0.0000
   4 O(17)  Bbb    -0.0412     2.980     1.063     0.994 -0.0110  0.9999  0.0000
          Bcc     0.6301   -45.592   -16.268   -15.208  0.0000  0.0000  1.0000

------------------------------------------------------------------------
 Temperature:     0K
------------------------------------------------------------------------
          Nuclear Quadrupole in Principal Axis System
     Atom            a.u.   MegaHertz   Gauss  10(-4) cm-1        Axes
          Baa    -0.4414   -59.232   -21.135   -19.758  0.9994 -0.0345 -0.0001
   1 C(13)  Bbb    -0.1018   -13.667    -4.877    -4.559  0.0001  0.0000  1.0000
          Bcc     0.5432    72.899    26.012    24.316  0.0345  0.9994  0.0000

          Baa    -1.2237  -264.534   -94.392   -88.239  0.4605  0.8877  0.0000
   2 P(31)  Bbb     0.1341    28.996    10.347     9.672  0.8877 -0.4605  0.0000
          Bcc     1.0895   235.538    84.046    78.567  0.0000  0.0000  1.0000

          Baa    -0.1790   -14.662    -5.232    -4.891  0.9999  0.0123  0.0000
   3 H(2)   Bbb     0.0753     6.167     2.201     2.057 -0.0123  0.9999  0.0000
          Bcc     0.1037     8.495     3.031     2.834  0.0000  0.0000  1.0000

          Baa    -0.5843    42.278    15.086    14.102  0.9999  0.0123 -0.0001
   4 O(17)  Bbb    -0.0439     3.176     1.133     1.059 -0.0123  0.9999  0.0000
          Bcc     0.6282   -45.454   -16.219   -15.162  0.0001  0.0000  1.0000

------------------------------------------------------------------------
 Temperature:   298K
------------------------------------------------------------------------
          Nuclear Quadrupole in Principal Axis System
     Atom            a.u.   MegaHertz   Gauss  10(-4) cm-1        Axes
          Baa    -0.4403   -59.080   -21.081   -19.707  0.9994 -0.0344 -0.0001
   1 C(13)  Bbb    -0.1017   -13.650    -4.871    -4.553  0.0001  0.0000  1.0000
          Bcc     0.5420    72.730    25.952    24.260  0.0344  0.9994  0.0000
```

```
        Baa    -1.2180  -263.313   -93.957   -87.832  0.4594  0.8882  0.0000
  2 P(31)  Bbb     0.1296    28.010     9.995     9.343  0.8882 -0.4594  0.0001
        Bcc     1.0884   235.303    83.962    78.489  0.0000  0.0000  1.0000

        Baa    -0.1782   -14.591    -5.206    -4.867  0.9999  0.0129  0.0000
  3 H(2)   Bbb     0.0745     6.104     2.178     2.036 -0.0129  0.9999  0.0000
        Bcc     0.1036     8.488     3.029     2.831  0.0000  0.0000  1.0000

        Baa    -0.5850    42.327    15.103    14.119  0.9999  0.0105 -0.0001
  4 O(17)  Bbb    -0.0402     2.906     1.037     0.969 -0.0105  0.9999  0.0000
        Bcc     0.6251   -45.232   -16.140   -15.088  0.0001  0.0000  1.0000


  ==================================================
            Resonance Analysis
  ==================================================

Thresholds
----------
Maximum Difference PT2 vs. Variational (cm-1) :      1.000
Minimum value for Darling-Dennison term (cm-1):     10.000

Fermi resonances
----------------
 I   J + K   Freq.Diff.  Reduced Cubic Const.  PT2-Variat.Diff.
 4   5   5     -12.431        -35.272              -3.148

  1 Active Fermi resonances over    1

Darling-Dennison resonances
---------------------------
 No Darling-Dennison resonance found

  ==================================================
            Anharmonic X Matrix
  ==================================================

PT2 model: Deperturbed VPT2 (DVPT2)
Ref.: V. Barone, J. Chem. Phys. 122, 1, 014108 (2005)

Coriolis contributions to X Matrix (in cm^-1)
---------------------------------------------
            1             2             3             4             5
   1  0.000000D+00
   2  0.000000D+00  0.000000D+00
   3  0.741427D-01  0.109373D+00  0.000000D+00
   4  0.159505D+00  0.148318D+00  0.324756D-01  0.000000D+00
   5  0.617226D+00  0.405093D+00  0.129903D-03  0.520670D-03  0.000000D+00
   6  0.844795D+00  0.270615D-01  0.161729D+01  0.291742D+01  0.479159D+01
            6
   6  0.000000D+00

3rd Deriv. contributions to X Matrix (in cm^-1)
-----------------------------------------------
            1             2             3             4             5
   1 -0.232485D+02
   2 -0.764224D+00 -0.469258D+02
   3 -0.834599D+00  0.796081D+01 -0.444914D+01
   4  0.313163D+01  0.364128D+01 -0.557813D+01 -0.723107D+00
   5  0.254078D+02  0.409471D+02 -0.380826D+01 -0.103212D+02 -0.143773D+02
   6  0.299406D+02 -0.589695D-01 -0.217509D+01 -0.181376D+01 -0.927582D+01
            6
   6 -0.828542D+01

4th Deriv. contributions to X Matrix (in cm^-1)
-----------------------------------------------
            1             2             3             4             5
```

```
    1  0.120649D+02
    2 -0.495610D-01  0.373357D+02
    3 -0.507890D+00 -0.873211D+01  0.647246D+00
    4 -0.646607D+01 -0.101559D+02  0.239038D+01 -0.105010D+01
    5 -0.291802D+02 -0.390929D+02  0.391662D+01  0.451613D+01  0.130077D+02
    6 -0.363710D+02 -0.923961D-01 -0.255410D+01 -0.180181D+00  0.986822D+01
              6
    6  0.914529D+01
```

Total Anharmonic X Matrix (in cm^-1)

```
------------------------------------
              1             2             3             4             5
    1 -0.111836D+02
    2 -0.813785D+00 -0.959016D+01
    3 -0.126835D+01 -0.661921D+00 -0.380190D+01
    4 -0.317493D+01 -0.636634D+01 -0.315528D+01 -0.177321D+01
    5 -0.315518D+01  0.225933D+01  0.108496D+00 -0.580453D+01 -0.136956D+01
    6 -0.558563D+01 -0.124304D+00 -0.311190D+01  0.923476D+00  0.538399D+01
              6
    6  0.859871D+00
```

```
    ==================================================
          Deperturbed terms for anharmonicity
    ==================================================
```

Deperturbed terms in FMat (Fermi Resonances)

```
--------------------------------------------

       4                          5   5
   0.66546D+03   -0.88181D+01    0.68080D+03
```

Vibrational Energies (cm^-1)

```
----------------------------
 Mode(Quanta)   E(depert.)  E(after diag.)
  4(1)          665.460      661.443
  5(2)          680.801      684.818
```

```
    ==================================================
             Anharmonic Zero Point Energy
    ==================================================
```

Anharmonic Zero Point Energy

```
----------------------------
Harmonic        : cm-1 =  3050.22227 ; Kcal/mol =   8.721 ; KJ/mol =  36.489
Anharm.Pot.     : cm-1 =   -13.22606 ; Kcal/mol =  -0.038 ; KJ/mol =  -0.158
Watson+Coriolis: cm-1 =    -0.70465 ; Kcal/mol =  -0.002 ; KJ/mol =  -0.008
X0F (Im. Freq.): cm-1 =     0.00000 ; Kcal/mol =   0.000 ; KJ/mol =   0.000
Total Anharm    : cm-1 =  3036.29156 ; Kcal/mol =   8.681 ; KJ/mol =  36.322
```

```
    ==================================================
         Vibrational Energies at Anharmonic Level
    ==================================================
```

Vibrational Energies and Rotational Constants (in cm^-1)

```
---------------------------------------------------------
Mode(Quanta)      E(harm)     E(anharm)     Aa(z)       Ba(x)       Ca(y)
Equilibrium Geometry                       4.376534    0.183879    0.176464
Ground State       3050.222    3036.292     4.361391    0.183571    0.175871
Fundamental Bands (DE w.r.t. Ground State)
  1(1) active      2124.686    2095.320     4.359819    0.182599    0.174975
  2(1) active      1720.887    1698.853     4.270256    0.183602    0.175769
  3(1) active       761.269     749.621     4.417123    0.183035    0.175418
  4(1) active       677.795     661.443     4.483919    0.183597    0.175567
  5(1) active       345.113     341.770     4.086609    0.184216    0.176091
  6(1) active       470.694     471.157     4.520334    0.183764    0.176225
Overtones (DE w.r.t. Ground State)
  1(2)             4249.372    4168.273     4.358247    0.181627    0.174079
  2(2)             3441.773    3378.525     4.179121    0.183632    0.175667
```

3(2)		1522.539	1491.638	4.472854	0.182499	0.174964
4(2)		1355.590	1327.373	4.606447	0.183624	0.175262
5(2)		690.226	684.818	3.811827	0.184860	0.176310
6(2)		941.389	944.033	4.679277	0.183957	0.176578

Combination Bands (DE w.r.t. Ground State)

2(1)	1(1)	3845.573	3793.359	4.268684	0.182629	0.174873
3(1)	1(1)	2885.955	2843.673	4.415551	0.182063	0.174521
3(1)	2(1)	2482.156	2447.812	4.325987	0.183065	0.175316
4(1)	1(1)	2802.481	2757.605	4.482347	0.182625	0.174670
4(1)	2(1)	2398.682	2357.946	4.392784	0.183628	0.175465
4(1)	3(1)	1439.064	1411.926	4.539651	0.183061	0.175113
5(1)	1(1)	2469.799	2433.935	4.085037	0.183243	0.175194
5(1)	2(1)	2066.000	2042.882	3.995474	0.184246	0.175989
5(1)	3(1)	1106.382	1091.499	4.142341	0.183679	0.175637
5(1)	4(1)	1022.908	1001.425	4.209137	0.184242	0.175786
6(1)	1(1)	2595.380	2560.891	4.518762	0.182792	0.175328
6(1)	2(1)	2191.581	2169.885	4.429199	0.183795	0.176122
6(1)	3(1)	1231.964	1217.666	4.576066	0.183228	0.175771
6(1)	4(1)	1148.489	1137.540	4.642862	0.183791	0.175920
6(1)	5(1)	815.807	818.311	4.245552	0.184409	0.176444

Fundamental Bands

Mode(Quanta)	E(harm)	E(anharm)	I(harm)	I(anharm)
1(1)	2124.686	2095.320	707.59437296	683.21702311
2(1)	1720.887	1698.853	7.72098939	8.08585516
3(1)	761.269	749.621	2.06939703	1.87600731
4(1)	677.795	661.443	9.51484366	9.25582879
5(1)	345.113	341.770	7.05123387	6.75066349
6(1)	470.694	471.157	0.21359226	0.19114589

Overtones

Mode(Quanta)	E(harm)	E(anharm)	I(anharm)
1(2)	4249.372	4168.273	5.89113341
2(2)	3441.773	3378.525	0.00753993
3(2)	1522.539	1491.638	0.39553377
4(2)	1355.590	1327.373	0.08960907
5(2)	690.226	684.818	0.03201394
6(2)	941.389	944.033	0.58339411

Combination Bands

Mode(Quanta)		E(harm)	E(anharm)	I(anharm)
2(1)	1(1)	3845.573	3793.359	0.22451524
3(1)	1(1)	2885.955	2843.673	3.68530025
3(1)	2(1)	2482.156	2447.812	0.07897302
4(1)	1(1)	2802.481	2757.605	1.41992537
4(1)	2(1)	2398.682	2357.946	0.21123331
4(1)	3(1)	1439.064	1411.926	0.12877370
5(1)	1(1)	2469.799	2433.935	0.01784993
5(1)	2(1)	2066.000	2042.882	0.16755293
5(1)	3(1)	1106.382	1091.499	0.88171421
5(1)	4(1)	1022.908	1001.425	1.30351877
6(1)	1(1)	2595.380	2560.891	0.00125199
6(1)	2(1)	2191.581	2169.885	0.00140216
6(1)	3(1)	1231.964	1217.666	0.01310488
6(1)	4(1)	1148.489	1137.540	0.03173962
6(1)	5(1)	815.807	818.311	0.00209583

3.2 Isomers of HPCO

Table S1. Computed isomers of HPCO.

monomer	a.u.<br>E	kJ<br>ΔE	ppm<br>^{31}P	ppm<br>^{1}H
HPCO	-455.0317333	0.0	-310	1.8
PCOH	-454.9982745	87.8	-245	4.5
PC(H)O	-454.9067496	328.1	+858	12.4

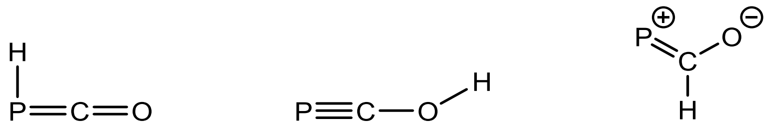

1 2 3

Figure S34. Computed isomers of HPCO.

```
1_01
0 1
 C          -0.99173548    0.10051244   -0.00004274
 P          -2.62770635   -0.15755215    0.00059353
 H          -3.24369053    0.86098386    0.63749845
 O          -0.22142652    1.30530456   -0.00020426

1_02
0 1
 C          -0.99173548    0.10051244   -0.00004274
 P          -2.62770635   -0.15755215    0.00059353
 O          -0.22142652    1.30530456   -0.00020426
 H           0.71350511    1.08735309   -0.00017504

1_03
0 1
 C           0.00000000    0.89334000    0.00000000
 O           1.16218400    0.32462700    0.00000000
 P          -0.60769600   -0.66192100    0.00000000
 H          -0.18204200    1.97175900    0.00000000
```

3.3 Isomers of $(HPCO)_2$

Table S2. Computed isomers of $(HPCO)_2$. Isomers with a suitable coupling pattern (number of multiplets due to ${}^{1}J_{P\text{–}H}$ coupling) are shown in bold.

	energy [a.u.]	rel. E [kJ mol^{-1}]		
dimer	-910.089692288		δ(^{31}P)	δ (^{31}P)
1	-910.079870700	25.8	172	172
2	-910.079272490	27.4	155	155
**3**	**-910.075835700**	**36.4**	**101**	**367**
4	-910.045414150	116.3	177	177
5	-910.045379637	116.3	71	129
6	-910.071516941	47.7	23	112
**7**	**-910.046679684**	**112.9**	**84**	**174**
8	-910.062443388	71.5	87	283
**9**	**-910.046917064**	**112.3**	**158**	**415**
10	-910.028743625	160.0	-47	-47
11	-910.039732055	131.2	148	148
12	-910.085548158	10.9	-35	-35
13	-910.089692288	0.0	-20	-20
**14**	**-910.071515472**	**47.7**	**-11**	**83**
**15**	**-910.021492230**	**179.1**	**12**	**783**
16	-909.938939441	395.8	51	66
17	-909.901606526	493.8	-43	-43
18	-909.899599633	499.1	-60	-1
19	-909.983347118	279.2	148	260
20	-909.989131436	264.0	148	148

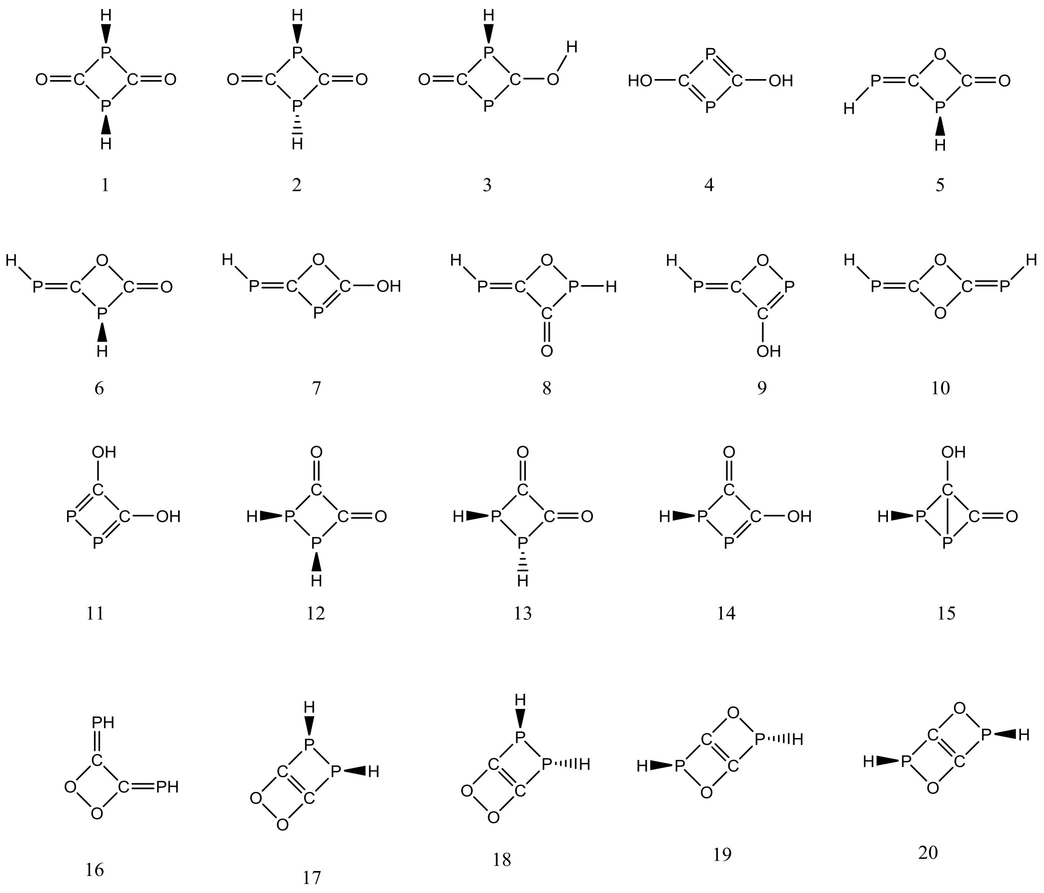

Figure S35. Computed isomers of $(HPCO)_2$.

2_01

0 1

C	1.07666700	0.03208700	-0.00052000
C	-1.07636800	-0.03185600	-0.00002500
P	-0.13679400	1.43767300	-0.00022200
P	0.13669500	-1.43740200	-0.00022900
O	-2.36968500	-0.23586900	0.00047800
O	2.36972300	0.23530400	0.00058500
H	-2.84600300	0.60538500	0.00056800
H	2.84538100	-0.60630600	0.00098200

2_02

0 1

C	-1.25692900	-0.05486000	-0.00082200
C	1.03260700	-0.09026400	0.01664200
P	-0.02520300	-1.44540800	-0.04311600
P	-0.01795000	1.41995800	-0.14454500
H	-0.02030400	1.80937300	1.23148300
O	2.35226700	-0.13593700	0.08445100
O	-2.44165400	-0.02728100	0.09542200
H	2.72863100	0.74885600	0.04953700

2_03

0 1

C	-1.20619300	0.00011400	0.03212700
C	1.20625700	-0.00012200	0.03219800
P	-0.00018000	-1.45012300	-0.23497000
H	-0.00037700	-1.88373700	1.11867600
P	0.00012400	1.45012500	-0.23497100
H	0.00031900	1.88381200	1.11865100
O	2.36751200	-0.00016800	0.27659400
O	-2.36744800	0.00016000	0.27663600

2_04

0 1

C	1.07754400	0.03509600	0.00010200
C	-1.07753000	-0.03507000	0.00007200
P	-0.13513500	1.44323200	0.00025100
P	0.13509200	-1.44317200	0.00025100
O	-2.37249000	-0.23387500	-0.00037700
O	2.37256300	0.23376300	-0.00038600
H	2.84841200	-0.61088000	-0.00124200
H	-2.84843300	0.61071200	-0.00123700

2_05

0 1

C	1.30579100	0.22718000	-0.00003100
C	-0.67529600	-0.01556100	-0.00011700
P	0.57943200	-1.35143400	-0.00001700
O	2.47349700	0.81721700	0.00017400
H	3.16850700	0.15099000	-0.00011000
P	-2.34325600	0.14963500	0.00008900
H	-2.30230100	1.57723200	-0.00012300
O	0.25252600	1.06141400	-0.00016900

2_06

0 1

C	-1.45502200	0.35503200	-0.01108100
C	0.63921100	0.05315700	-0.00252900
O	-2.54243100	0.80317800	0.02368400
P	2.31181300	0.14847200	0.00857900
H	2.34193200	1.57525700	0.04925600
O	-0.27757200	1.09110800	-0.01720900
P	-0.59088800	-1.32493600	-0.09706400
H	-0.70092000	-1.53172700	1.30787300

2_07

0 1

O	-0.26848400	1.06468800	-0.00055300
C	0.67597500	-0.00642200	-0.00055100
P	2.31803900	0.33440700	0.00029300
H	2.67293200	-1.04589200	0.00003000
O	-2.48351300	0.79675300	0.00050100
H	-3.16973400	0.12162700	0.00005700

C -1.30851300 0.22205300 -0.00010500
P -0.56417200 -1.35181000 -0.00000900
2_08
0 1
O 0.34100500 -1.29531400 0.08112100
C -0.51227600 -0.23677400 0.03619600
P -2.19787400 -0.28952100 -0.03418200
H -2.25946100 1.13511100 -0.04133700
P 1.84726800 -0.44387100 0.04507700
C 0.47231800 0.89712000 0.03038000
O 0.37943300 2.07888300 0.02557900
H 1.99479200 -0.36485000 -1.37514600
2_09
0 1
O 0.24759400 -1.43211700 0.00036100
C -0.47426500 -0.26465200 0.00014900
P -2.16098300 -0.18064400 -0.00016300
H -2.14366400 1.24520900 0.00018700
P 1.77103700 -0.55322700 -0.00019600
C 0.61459800 0.69907100 0.00006000
O 0.47848600 2.02129400 0.00023300
H 1.34222200 2.44293000 -0.00080200
2_10
0 1
O -0.00905400 0.99966500 0.00006800
O 0.00907700 -0.99974100 0.00002100
C -0.93958600 -0.00601200 0.00002900
C 0.93959800 0.00591900 0.00003700
P 2.61933000 0.09228700 -0.00004100
H 2.69735600 -1.32933400 0.00004400
P -2.61935900 -0.09221400 -0.00002200
H -2.69717400 1.32940800 -0.00021700
2_11
0 1
C -0.45505600 -0.73709500 -0.00039000
C -0.45367600 0.73792700 0.00038800
O -1.56954700 1.48874100 -0.00823700
O -1.57232500 -1.48588600 0.00808800
P 1.17198500 1.21357000 0.03178500
P 1.16968000 -1.21565800 -0.03177200
H -2.26802200 1.01663300 -0.47598200
H -2.26958700 -1.01313800 0.47699000
2_12
0 1
C -0.75563800 0.79418100 -0.04264200
C -0.77043900 -0.78094400 -0.04607100
H 1.12997100 -1.38958400 1.32691100
O -1.70501100 -1.50398100 0.01168400
O -1.67541400 1.53420300 0.03547100
P 1.11972600 -1.13367100 -0.07339000
P 1.14007400 1.11196900 -0.09160300
H 1.17289000 1.39392400 1.30301300
2_13
0 1
C 0.76779000 0.79053800 0.00255800
C 0.76747600 -0.79093700 -0.00220100
H -1.26622500 -1.45503300 -1.20933700
O 1.69181800 -1.52461400 -0.07245400
O 1.69240700 1.52392600 0.07229500
P -1.12543700 -1.10031000 0.16071500
P -1.12480000 1.10081600 -0.16077600
H -1.26562300 1.45532600 1.20937100
2_14
0 1
C 0.82590600 -0.43625300 -0.02825200
C 0.27548500 0.93218900 -0.00748600
H -1.87791600 0.79588000 1.21696200
O 0.88951900 1.95881200 0.09386900

O 2.12919200 -0.67365900 -0.06343600
P -1.57345700 0.52596500 -0.15158000
P -0.52353000 -1.47524100 0.07258500
H 2.57469200 0.18641000 -0.06108600
2_15
0 1
C 1.47390500 0.84946600 -0.00323200
C 1.00629200 -0.39621400 0.01991800
H -0.71647500 -2.08955900 -0.25619400
O 1.87460800 -1.46888500 -0.03293800
H 1.99950600 -1.79441200 0.86379500
O 1.83899000 1.94122300 -0.02835800
P -0.76879800 -0.68151200 -0.06965700
P -2.28940100 0.50722900 0.05516800
2_16
0 1
C -0.71706300 0.19145600 0.00008100
C 0.73045200 0.20567900 -0.00009500
O -0.73782700 1.56749100 0.00017000
O 0.71874900 1.58778800 -0.00019900
P 2.09577200 -0.78803600 0.00008000
P -1.97756700 -0.92955300 -0.00004600
H 1.27353400 -1.95196600 -0.00036500
H -2.97432300 0.09075300 0.00016900
2_17
0 1
C -0.57346200 -0.66867700 0.38003700
C -0.57255900 0.66846200 0.37901300
H 1.66388900 1.48056500 1.11874200
P 1.11861300 1.14244400 -0.15381100
O -1.87406500 0.73012800 -0.13588800
O -1.87522500 -0.72891900 -0.13480200
P 1.11752700 -1.14282900 -0.15448700
H 1.66445400 -1.48316200 1.11694600
2_18
0 1
C -0.61740900 -0.66476700 0.42715700
C -0.53418600 0.66331600 0.33011200
H 1.10720900 1.29666700 -1.34198800
P 1.23803500 1.10837500 0.06654300
O -1.81335100 0.80309600 -0.18903300
O -1.92679200 -0.65233500 -0.09875000
P 1.03561900 -1.16492600 -0.19410000
H 1.61869300 -1.64578600 1.01398100
2_19
0 1
C 0.05400300 0.65356200 0.44560000
C -0.08169300 -0.68660600 0.43686100
H -2.60887200 -0.64005700 0.76524400
H 1.41873200 0.48291300 -1.50730100
P -1.71860900 -0.36211400 -0.31885300
P 1.81216200 0.38264800 -0.13635600
O 1.16347000 -1.21013200 0.12603500
O -1.16934800 1.21605600 0.15839400
2_20
0 1
C 0.07245400 0.67313000 0.43132700
C -0.07301100 -0.67410500 0.43175900
H -2.58051100 -0.62828700 0.81999600
H 2.57857600 0.62885400 0.82126500
P -1.73106500 -0.36242000 -0.29902000
P 1.73141300 0.36279100 -0.29882700
O 1.16344400 -1.21749900 0.13405800
O -1.16343700 1.21746400 0.13443400

3.4 Isomers of $(HPCO)_3$

Table S3. Computed isomers of $(HPCO)_3$. Isomers with a suitable coupling pattern (number of multiplets due to $^{1}J_{P–H}$ coupling) are shown in bold.

trimer	energy [a.u.]	rel. E [kJ mol^{-1}]	δ(^{31}P) ppm	δ(^{31}P) ppm	δ(^{31}P) ppm
1	-1365.14456616	23.8	140	158	178
2	-1365.12504082	75.0	4	184	184
3	-1365.12944181	63.5	-32	182	182
4	-1365.13496085	49.0	8	23	218
5	-1365.13496080	49.0	8	24	218
6	-1365.13710403	43.4	17	49	203
7	-1365.10657401	123.5	-81	13	69
8	-1365.13911130	38.1	-8	1	155
**9**	**-1365.12601701**	**72.5**	**-92**	**71**	**176**
**10**	**-1365.12462676**	**76.1**	**-92**	**82**	**182**
**11**	**-1365.13883026**	**38.8**	**-85**	**114**	**124**
**12**	**-1365.13609998**	**46.0**	**-85**	**118**	**131**
**13**	**-1365.15361528**	**0.0**	**-80**	**82**	**173**
14	-1365.13961226	36.8	-110	115	298
15	-1365.13983020	36.2	-111	148	318
**16**	**-1365.13131840**	**58.5**	**-92**	**184**	**468**
17	-1365.12253762	81.6	101	259	328
18	-1365.12467566	76.0	95	259	325
19	-1365.12498118	75.2	46	243	339
20	-1365.13077310	60.0	149	184	397
**21**	**-1365.07164120**	**215.2**	**-75**	**223**	**246**
**22**	**-1365.06173429**	**241.2**	**-75**	**165**	**291**
**23**	**-1365.12918536**	**64.1**	**-110**	**181**	**297**
24	-1365.12841380	66.2	168	168	397
25	-1365.13221488	56.2	157	166	367
**26**	**-1365.11422561**	**103.4**	**-85**	**-49**	**9**
27	-1365.11701760	96.1	-144	41	233
28	-1365.12141031	84.6	-118	47	106
**29**	**-1365.10320186**	**132.4**	**-135**	**-88**	**324**
**30**	**-1365.11054578**	**113.1**	**-94**	**-43**	**180**
**31**	**-1365.07857380**	**197.0**	**-113**	**403**	**417**
32	-1365.14515367	22.2	73	73	73
33	-1365.14092327	33.3	43	43	50
34	-1365.13814103	40.6	5	69	69

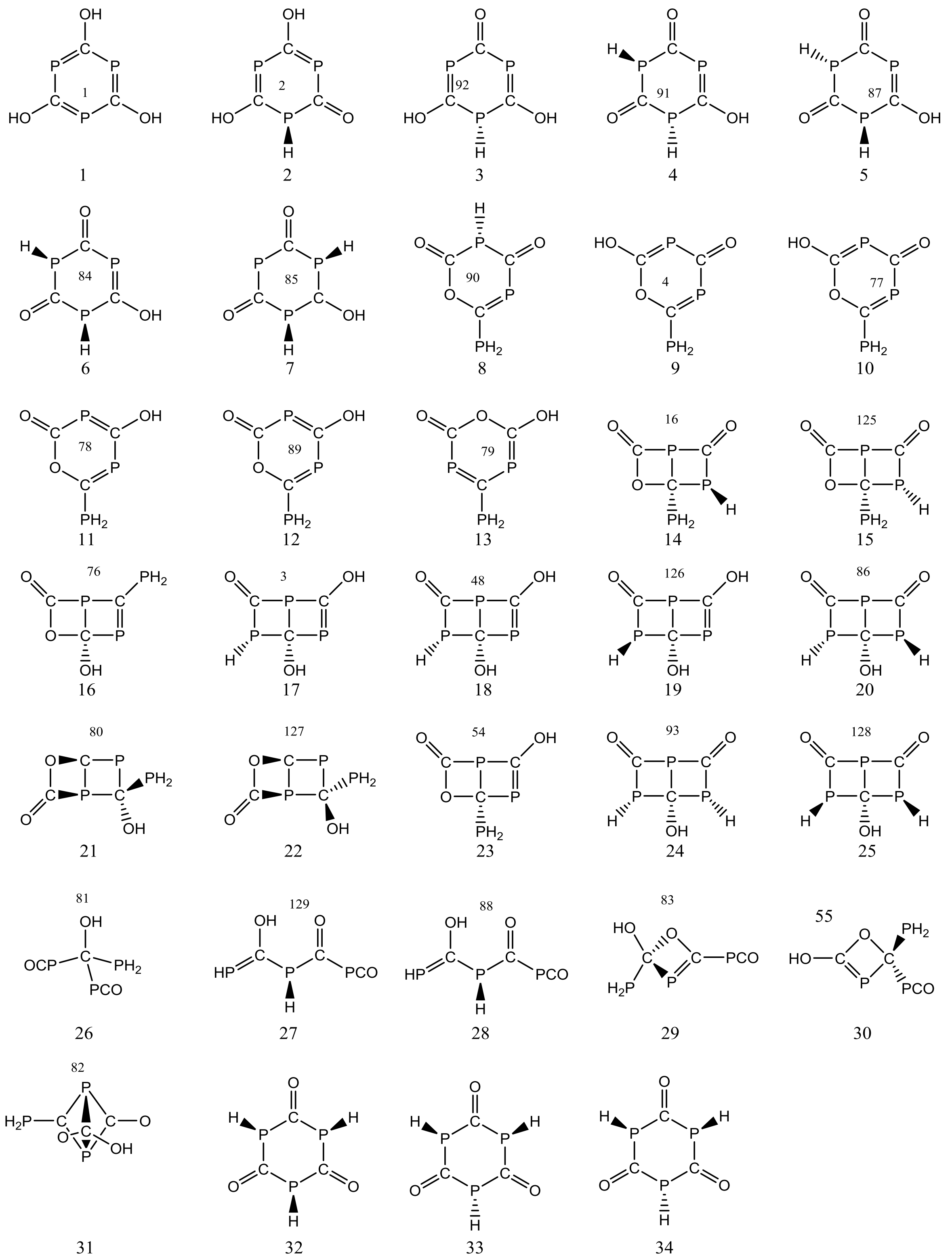

Figure S36. Computed isomers of $(HPCO)_3$.

3_01
0 1

C	1.49811000	-0.52984100	0.00007000
C	-0.29473900	1.58434600	0.00023300
C	-1.19778000	-1.06197900	-0.00017900
O	-0.49416300	2.92485700	0.00037000
O	2.76045800	-1.02516900	0.00022900
O	-2.19293900	-1.98259100	-0.00037000
P	0.35739900	-1.86413200	0.00016000
P	1.41492700	1.22186800	-0.00076000
P	-1.74084600	0.60466200	0.00021800
H	3.39830100	-0.30118600	0.00283600
H	-1.43905500	3.11762700	0.00042500
H	-3.05182900	-1.54433300	-0.00011000

3_02
0 1

C	1.34608500	-0.78737200	0.06923300
C	-1.44282900	-0.72132600	-0.02770900
C	0.16854200	1.70112000	-0.16753900
O	-2.65813900	-1.25516700	-0.24406600
O	2.41795700	-1.59324700	0.23065300
O	0.09073200	2.81886300	-0.60454800
P	1.79906500	0.87681400	0.03796900
P	-0.13464300	-1.79333500	-0.27499500
P	-1.37799200	0.96196000	0.62871300
H	-2.32509500	1.51344400	-0.27907200
H	2.14185500	-2.51879600	0.23144200
H	-3.34839500	-0.59434700	-0.12790100

3_03
0 1

C	0.00068600	1.71429100	0.12946100
C	-1.43427600	-0.69286400	-0.06591600
C	1.43369900	-0.69384400	-0.06607800
O	-2.44975600	-1.56585000	-0.22369300
O	0.00067700	2.86872300	0.48359500
O	2.44886500	-1.56717200	-0.22372000
P	1.67753500	0.97970500	-0.24275900
P	-1.67678500	0.98065500	-0.24317700
P	-0.00054500	-1.63035800	0.55408900
H	3.26286300	-1.08655200	-0.42229400
H	-0.00091200	-2.62984900	-0.45415500
H	-3.26398000	-1.08474100	-0.42008700

3_04
0 1

C	-0.45066100	1.60268100	0.21477500
C	-1.26188300	-1.18985500	-0.04711100
C	1.53348300	-0.31392900	-0.09313900
O	-2.07602700	-2.07289400	-0.06399400
O	-0.79539100	2.58350800	0.81738800
O	2.72171400	-0.89060200	-0.34855200
P	1.35822200	1.38043100	-0.21006500
P	-1.73379800	0.51918200	-0.62384500
P	0.43827000	-1.59395000	0.62828100
H	3.36264000	-0.22026400	-0.62071300
H	-2.75697700	0.68697000	0.34256000
H	0.72593600	-2.60513500	-0.32329800

3_05
0 1

C	0.45034300	1.60285100	0.21470900
C	1.26204700	-1.18974700	-0.04703200
C	-1.53346800	-0.31410300	-0.09316200
O	2.07634100	-2.07265400	-0.06374700
O	0.79486500	2.58396400	0.81704000
O	-2.72163400	-0.89094700	-0.34839300
P	-1.35836000	1.38021700	-0.20998400
P	1.73381500	0.51938100	-0.62367500
P	-0.43803500	-1.59417500	0.62796500
H	-0.72530800	-2.60510500	-0.32401300

H 2.75684900 0.68748600 0.34283700
H -3.36295100 -0.22063000 -0.61971100
3_06
0 1
C 1.16154600 -1.27397100 0.03605400
C 0.60243000 1.55933800 0.20333500
C -1.53620000 -0.35334900 -0.02239200
O 0.93203700 2.48817600 0.88696800
O 1.97927700 -2.11174700 0.32106600
O -2.87468900 -0.43465400 0.09928800
P -0.61831500 -1.79271800 0.10894000
P 1.80717500 0.38135600 -0.57783200
P -1.15995600 1.39545600 -0.41021400
H -1.78074000 1.94985600 0.73812400
H -3.13320600 -1.34783000 0.28484400
H 2.82073700 0.51026100 0.40305700
3_07
0 1
C -1.73641600 0.12623100 -0.01121800
C 0.81284400 -1.37721700 0.10858100
C 0.75203800 1.60456500 0.05599800
O 1.25549200 -2.16382900 1.07532400
O -2.91246100 -0.08552200 0.10191400
O 1.61087100 2.40462200 0.25155600
P -1.00740200 1.76032900 0.21396500
P -0.84919000 -1.59020700 -0.47959600
P 1.70910500 -0.10921200 -0.70399000
H 2.77179800 -0.04424900 0.24426200
H 2.17254700 -1.94943600 1.29157300
H -1.33405200 -2.28360900 0.65795800
3_08
0 1
C -1.13952600 -0.07239300 -0.02369000
C 0.68174800 1.55682000 -0.13962900
C 1.39566000 -1.18220300 -0.10245400
P -2.95855900 0.06234500 0.21658900
H -3.24209800 0.60899400 -1.06569600
H -3.25890500 -1.27853000 -0.13766700
P -0.41494200 -1.61452800 -0.07756900
P 1.89635700 0.43813300 0.68633300
O 2.21603900 -1.96870300 -0.49011400
O 0.95271500 2.62653300 -0.57503700
O -0.66031600 1.19191900 -0.10988500
H 2.96336000 0.76896000 -0.18200100
3_09
0 1
C 1.15045600 -0.12924300 0.01977900
C -1.42115200 -1.21925900 -0.02142500
C -0.65830000 1.44296000 0.00098900
P -2.09565400 0.48937200 0.01665500
P 0.38744600 -1.64049200 0.07973400
P 2.97848700 0.02666600 -0.13358600
H 3.20437400 0.53093500 1.17949900
H 3.24712500 -1.32394000 0.20903000
O 0.63349000 1.12855600 -0.02933600
O -0.71857400 2.77165600 0.00772000
H -1.64588600 3.03611600 0.01670200
O -2.20064500 -2.14309200 -0.08380000
3_10
0 1
C 1.14650700 -0.16092600 0.01956800
C -0.65490500 1.45023600 0.00302500
C -1.45367900 -1.17607200 -0.01740100
P 2.97671200 -0.03076200 -0.12721300
H 3.22074700 0.37904000 1.21611400
H 3.21944300 -1.40525100 0.12562900
P 0.34637100 -1.64955200 0.06960400
P -2.11524000 0.53687900 0.01229600

O	-2.24699800	-2.09025900	-0.07169000
O	-0.75823400	2.77441600	0.00994300
O	0.64126800	1.10817100	-0.02192300
H	0.12634900	3.15969000	-0.02382600

3_11
0 1

C	-1.16129800	-0.10712100	0.01904000
C	0.55940700	1.66360900	0.01812300
C	1.39934400	-0.97606100	-0.00575200
P	-2.99419000	-0.14487900	-0.15029000
H	-3.14443300	-1.48813000	0.28648000
H	-3.27005100	0.43299900	1.12065200
P	-0.29573700	-1.57896400	0.08574000
P	2.04810700	0.61623600	-0.05374000
O	2.24198800	-2.02291400	-0.02146400
H	3.15731300	-1.71561200	-0.05898600
O	0.65322400	2.85004800	0.07515800
O	-0.75774500	1.16065300	-0.02397600

3_12
0 1

C	1.17144100	-0.09624400	0.01623100
C	-0.57322400	1.65701000	0.02491600
C	-1.41214200	-0.96732400	-0.00625700
P	3.00259500	-0.14603300	-0.16044300
H	3.28525400	0.44844000	1.10105600
H	3.14743300	-1.48403500	0.29493400
P	0.28671600	-1.55419300	0.11040900
P	-2.07052600	0.61972400	-0.07550900
O	-2.34146300	-1.94137400	-0.03187600
O	-0.65804200	2.84147900	0.10879800
O	0.76114700	1.16146500	-0.04164400
H	-1.92405700	-2.81008600	-0.00441000

3_13
0 1

C	-1.36094500	-1.10368300	0.01379200
C	-1.29543700	1.39223300	-0.02256200
C	1.08961100	-0.01586000	0.01892900
P	0.30803500	-1.60484000	-0.04881500
P	0.51533500	1.59438200	0.07432200
O	-2.06786700	2.29080800	-0.08700600
O	-1.91865200	0.08359000	0.02153300
O	-2.26917300	-2.06474100	0.04700600
H	-3.14632700	-1.65706600	0.04591300
P	2.92282600	-0.18115200	-0.11839500
H	3.24888000	1.15063500	0.26067500
H	3.15066400	-0.73282000	1.17351000

3_14
0 1

C	-0.62340300	1.56994400	-0.26807800
C	0.77564800	-0.07369000	-0.07693700
C	-1.50418900	-0.94172200	0.21814100
P	-0.53541400	0.40017600	1.22045700
P	0.00831800	-1.70954000	-0.60086400
P	2.61971700	-0.06413400	0.14583600
H	2.67827200	1.06634700	1.00887700
H	2.62421100	-1.00922300	1.20861200
O	0.41168400	1.02895200	-0.96234200
O	-2.66053100	-1.18935600	0.18372700
O	-1.28804300	2.48925200	-0.59372600
H	-0.28501700	-1.41262000	-1.95894800

3_15
0 1

C	-0.57656900	1.61560000	-0.25831900
C	0.76799200	-0.07974300	-0.07068200
C	-1.52484800	-0.92807900	0.20874700
P	-0.54690600	0.40248300	1.19834400
P	-0.07854800	-1.57765400	-0.85938400
P	2.60392700	-0.15584500	0.20036500

H	2.67420000	0.94482900	1.10042700
H	2.53499400	-1.12614300	1.23938900
O	0.47181700	1.06707400	-0.93192600
O	-2.65944200	-1.25293600	0.27734600
O	-1.20639600	2.55912900	-0.58213500
H	0.26642500	-2.48625500	0.18554500

3_16
0 1

C	1.05163700	1.43411900	-0.23300100
C	1.23805700	-0.70200300	0.08664000
C	-1.12129900	-0.30865500	0.08651000
P	0.19362200	0.44155400	1.14794900
P	-0.27426300	-1.50964000	-0.75236800
O	1.76733500	0.38711200	-0.73156000
O	1.06223200	2.54745300	-0.63106200
O	2.22382000	-1.44784600	0.64636100
H	2.65103600	-1.97575000	-0.03630500
P	-2.81809500	0.29015000	-0.04218700
H	-3.42643800	-0.91125500	-0.49814200
H	-3.18104000	0.12152000	1.32273500

3_17
0 1

C	0.68270300	1.52299400	-0.00310000
C	-1.58756400	-0.03178400	-0.00603000
C	0.58947200	-0.96273400	0.19353300
P	1.79398200	0.15996100	-0.76732900
P	-0.29006600	0.39046100	1.20059900
P	-0.93691800	-1.37716900	-0.83317500
O	1.12348700	-2.08841200	0.80656000
H	1.87936000	-1.82910700	1.34103800
O	-2.74610100	0.58319300	-0.22934900
O	0.58856000	2.68224500	-0.22950800
H	2.67407400	0.36809100	0.34719300
H	-2.89362300	1.27515700	0.42229100

3_18
0 1

C	-1.59092400	-0.01322400	-0.00217700
C	0.57734600	-0.96678000	0.18969100
P	-0.96034700	-1.36682000	-0.84074900
P	-0.28334500	0.39622800	1.20019200
O	-2.73638400	0.62097100	-0.22814300
O	1.24701600	-2.01171700	0.81606200
C	0.70624500	1.51799400	-0.00679600
P	1.80219600	0.13609400	-0.74440700
H	2.66816900	0.32341100	0.37857200
O	0.62168100	2.67662000	-0.23921300
H	0.59915600	-2.65515200	1.11615900
H	-2.85937400	1.33428400	0.40577500

3_19
0 1

C	-1.58409900	-0.07828400	-0.00851600
C	0.62649800	-0.94736600	0.16677800
P	-0.88564700	-1.36159600	-0.89404800
P	-0.30489400	0.36549100	1.21380300
O	-2.76414500	0.50219200	-0.20560500
O	1.28351100	-2.00879000	0.78469000
C	0.64785800	1.53938400	0.01673400
P	1.90493800	0.25077300	-0.52248200
O	0.45596400	2.67205400	-0.28011000
H	0.62430800	-2.64747000	1.07024600
H	-2.91354000	1.19475800	0.44535700
H	1.62909200	0.22663400	-1.91646800

3_20
0 1

C	1.34885000	1.00373900	-0.00782600
C	-1.41563400	0.93550700	0.01996800
C	0.02364100	-1.08578600	0.20494900
P	1.55168300	-0.68865700	-0.86086600

P	-0.00456900	0.52777900	1.23918500
P	-1.63674600	-0.81069500	-0.64370200
O	0.00613800	-2.32822000	0.83581800
H	0.77955500	-2.40306300	1.40161100
O	-1.94290900	1.96535700	-0.24073000
O	1.88105100	2.04376200	-0.20698200
H	2.49090300	-1.08429900	0.14234400
H	-1.22135400	-0.50699900	-1.97059100

3_21
0 1

C	1.24241700	-0.04382000	0.28900000
P	1.67668500	1.42658600	-0.79906300
H	0.39102700	1.57887700	-1.38573500
H	1.45269600	2.42723500	0.19091500
O	2.27612800	-0.16568700	1.19487700
H	2.28932200	-1.06167300	1.54400000
P	-0.56487100	0.02307100	1.19601000
C	-0.79291500	-1.09506200	-0.13823900
C	-2.11659200	0.46125000	0.07796800
O	-2.94086900	1.27303600	-0.11387500
P	0.73494100	-1.53170400	-0.78581100
O	-2.06423900	-0.81334300	-0.56707700

3_22
0 1

C	1.01464500	-0.13283900	0.30832000
P	-0.43737100	-0.72250800	-0.84004300
C	-0.87692200	0.95905600	-0.47054500
C	-2.18412400	-0.53077300	0.01699800
O	-3.05191600	-1.13918600	0.51617800
P	0.47851800	1.74579000	0.19327500
O	-2.23011200	0.86981900	-0.27498400
O	0.79103800	-0.61189000	1.60073300
H	1.39207100	-0.15959600	2.20051400
P	2.66249000	-0.54971300	-0.46872900
H	3.43344600	0.15070500	0.50588500
H	2.82625600	-1.81724200	0.16200300

3_23
0 1

C	-0.33158000	1.71885800	-0.22169500
C	0.79010900	-0.13537400	-0.10044500
C	-1.45143500	-0.82277300	0.13702600
P	-0.51803700	0.39746900	1.14186600
P	-0.26295400	-1.50894700	-0.88030000
P	2.57330100	-0.51116600	0.22035500
H	2.77091200	0.44426800	1.25851200
H	2.31597500	-1.58731800	1.11413200
O	0.70516000	1.11945700	-0.85884100
O	-2.74427600	-1.04210100	0.32813900
O	-0.83050600	2.75494000	-0.49811600
H	-3.05712100	-1.73992900	-0.26023100

3_24
0 1

C	1.36563800	1.03141500	-0.01187700
C	-1.45791600	0.91404600	-0.00674500
C	0.04451800	-1.06352300	0.21348100
P	1.62303000	-0.69101900	-0.79754200
P	-0.02318600	0.56208600	1.19512400
P	-1.52508400	-0.80274900	-0.82211500
O	-0.01674000	-2.29418000	0.85982800
H	0.76734500	-2.40391900	1.40517500
O	-2.08192800	1.90173200	-0.20585600
O	1.88448400	2.07329800	-0.23762000
H	2.50204200	-1.06329400	0.26645800
H	-2.39073800	-1.29600100	0.19641000

3_25
0 1

C	1.16206800	1.19390300	0.01377100
C	-1.51610900	0.71340600	0.02540500

C 0.19251100 -1.08783100 0.17287700
P 1.76680800 -0.45030600 -0.65130900
P -0.08452600 0.48682300 1.26985800
P -1.48281800 -1.02040700 -0.68458700
O 0.31315400 -2.33986000 0.77749600
H 1.14193600 -2.37681200 1.26328500
O -2.18003700 1.66696100 -0.21256300
O 1.43521300 2.31720600 -0.25470800
H 1.41090200 -0.28851700 -2.01822500
H -1.12225900 -0.64764900 -2.00861500
3_26
0 1
C -0.08809400 0.49041700 0.22276600
P -0.87402100 2.12624500 -0.32259100
H -2.21645600 1.66953700 -0.20542200
H -0.80574000 1.87573900 -1.71929500
O -0.48388200 0.38155600 1.56070300
H 0.00996500 -0.33343800 1.97462900
P 1.78638500 0.88188500 0.04715300
P -0.50109100 -1.04151300 -0.86030400
C 2.34145500 -0.71128200 0.03254200
C -1.97155300 -1.32467300 -0.08646900
O 2.85698000 -1.73974300 0.02676400
O -2.97906300 -1.57154500 0.40918200
3_27
0 1
C -1.53753700 -0.81237100 0.04778600
C 1.35278500 -0.42098200 0.31062900
C -0.72768700 1.80101400 0.04500700
O 1.16950000 0.01667100 1.57157700
O -2.46693800 -1.47623600 0.39783800
O 0.02078500 2.54226000 0.51086000
P -1.96288200 0.91679500 -0.66264100
P 0.12153200 -1.67993300 -0.19616200
P 2.61307000 0.11628900 -0.69640100
H 1.83424200 0.68141800 1.79396900
H 0.03795700 -2.33625300 1.06092500
H 2.23987000 -0.70994700 -1.78957300
3_28
0 1
C 0.74749700 1.31030400 0.04384600
C -1.43150000 -0.52036900 0.01674200
C 2.10130800 -1.01277300 0.05205200
O -0.79433300 -1.64151500 -0.36051300
O 0.65360400 2.48849000 -0.13951700
O 2.21133300 -2.01039200 0.60870200
P 2.13243700 0.40526800 -0.86137300
P -0.42218400 0.41114900 1.23500300
P -2.98594000 0.01114800 -0.42091400
H -3.28676300 -1.16408200 -1.19088600
H -1.33927400 -2.11509600 -0.99988800
H -1.30731700 1.51006800 1.35482300
3_29
0 1
C -1.55514700 -0.00006800 -0.34990100
P -2.61511900 0.02379000 1.19784400
H -1.55341500 0.38125100 2.07231000
H -3.04669600 1.36980900 1.03517300
O -2.39120100 -0.23846600 -1.39965300
H -1.89779900 -0.11901500 -2.21789900
P -0.11634400 1.24660200 -0.56221000
O -0.58476300 -1.11426700 -0.16409100
C 0.50446500 -0.32403500 -0.24211000
P 2.06200100 -1.21400400 0.05554800
C 2.94342600 0.21587700 0.25326100
O 3.62388600 1.12416900 0.41064200
3_30
0 1

C -1.28753200 -1.19699300 -0.18357000
C -0.19391700 0.55026900 -0.01663800
C 2.26307900 -0.47549400 -0.05151500
P -0.41400500 -0.85043900 1.25951000
P 1.48871600 0.84214700 -0.78724700
P -0.98422500 2.22941000 0.24038000
H -2.13235800 1.74431600 0.92870300
H -0.26971800 2.52448100 1.43322200
O -1.07920800 -0.15615200 -0.98659800
O 2.97164700 -1.28502900 0.35128500
O -2.05524200 -2.16968200 -0.61771800
H -2.34256800 -1.96535500 -1.51697900
3_31
0 1
C 0.86188100 0.15291300 0.00492600
C -0.79373800 1.11048100 -0.01181100
C -1.12698900 -1.35258200 0.00361600
P -0.43860700 -0.22114600 1.35981200
P -0.46018300 -0.20016200 -1.35234800
O -1.72039300 -2.37871100 0.00010400
O 0.56634900 1.58826600 0.00154800
O -1.77327200 2.02618800 -0.08108400
H -1.72539200 2.59068800 0.69869500
P 2.59389300 -0.43174000 -0.00981800
H 3.02995200 0.43647000 -1.04829800
H 3.04050500 0.41773700 1.04000400
3_32
0 1
3_33
0 1
C -1.48427900 -0.85944100 -0.11293000
C 1.48394300 -0.85990300 -0.11324500
C 0.00013100 1.61653100 0.25776300
P -0.00018600 -1.60243400 0.74331200
H -0.00047100 -2.84183300 0.05921800
P -1.57895500 0.97664600 -0.53443800
H -2.42977800 1.29300800 0.55461300
P 1.57979300 0.97655900 -0.53319500
H 2.42945600 1.29160400 0.55715400
O 2.45701700 -1.53509700 -0.30481700
O -0.00014500 2.52083300 1.04640000
O -2.45784200 -1.53416800 -0.30354400
3_34
0 1
C 0.86808600 -1.36590200 0.18863800
C 0.86735600 1.36632800 0.18876500
C -1.66461300 -0.00044100 0.05466800
O 1.36982300 2.03384800 1.04246500
O 1.37089500 -2.03329100 1.04227700
O -2.75579900 -0.00074800 0.55210200
P -0.88048200 -1.64539900 -0.42516600
H -1.36166500 -2.32871200 0.71836200
P 1.85695900 0.00048000 -0.70207800
H 0.99390100 0.00043900 -1.83679400
P -0.88137900 1.64505200 -0.42523200
H -1.36301700 2.32790400 0.71839400

3.5 Isomers of $(HPCO)_4$

Table S4. Computed isomers of $(HPCO)_4$. Isomers with a suitable coupling pattern (number of multiplets due to $^{1}J_{P–H}$ coupling) are shown in bold.

tetramers	energy [a.u.]	rel. E [kJ mol^{-1}]	δ(^{31}P) ppm	δ(^{31}P) ppm	δ(^{31}P) ppm	δ(^{31}P) ppm
1	-1820.17906551	70.2	22	76	113	117
2	-1820.17429888	82.7	40	42	82	119
3	-1820.17464044	81.8	63	70	70	105
4	-1820.18160701	63.5	88	88	88	88
5	-1820.16377872	110.3	5	5	39	285
6	-1820.16266262	113.3	11	48	65	262
7	-1820.16917752	96.2	22	76	135	186
8	-1820.16376120	110.4	9	72	144	190
9	-1820.14093438	170.3	-11	67	294	355
10	-1820.15130850	143.1	-18	-2	191	259
11	-1820.14861646	150.1	-83	18	176	236
12	-1820.14637624	156.0	-11	59	267	353
13	-1820.14405695	162.1	-12	104	282	325
14	-1820.14873532	149.8	-15	101	263	328
15	-1820.13623534	182.6	-64	37	217	644
16	-1820.16399405	109.8	-67	6	55	300
17	-1820.17098224	91.4	-2	30	114	245
18	-1820.17803152	72.9	8	69	138	206
19	-1820.16669557	102.7	-53	19	45	276
20	-1820.18096250	65.2	80	80	97	104
21	-1820.14772235	152.5	9	233	248	302
22	-1820.13152793	195.0	11	198	213	275
23	-1820.14494191	159.8	210	212	234	237
**24**	**-1820.12822279**	**203.7**	**-113**	**64**	**223**	**273**
**25**	**-1820.13409357**	**188.3**	**-114**	**117**	**198**	**265**
**26**	**-1820.16268099**	**113.2**	**-112**	**77**	**184**	**276**
**27**	**-1820.14807239**	**151.6**	**-97**	**98**	**103**	**284**
**28**	**-1820.12822276**	**203.7**	**-113**	**64**	**223**	**273**
**29**	**-1820.14650155**	**155.7**	**-98**	**95**	**148**	**266**
**30**	**-1820.15134643**	**143.0**	**-115**	**-40**	**279**	**345**
**31**	**-1820.16456257**	**108.3**	**-114**	**18**	**206**	**302**
**32**	**-1820.13409381**	**188.3**	**-114**	**117**	**198**	**266**
**33**	**-1820.14952168**	**147.8**	**-95**	**-2**	**250**	**271**
34	-1820.13458024	187.0	-27	222	265	300
**35**	**-1820.18169166**	**63.3**	**-82**	**54**	**127**	**142**
**36**	**-1820.18149224**	**63.8**	**-64**	**29**	**180**	**211**
**37**	**-1820.18820477**	**46.2**	**-92**	**85**	**95**	**122**
**38**	**-1820.18982340**	**42.0**	**-42**	**130**	**163**	**200**
**39**	**-1820.18751515**	**48.0**	**-96**	**86**	**116**	**123**
**40**	**-1820.18807958**	**46.5**	**-62**	**153**	**161**	**187**
**41**	**-1820.18353979**	**58.4**	**-46**	**49**	**174**	**191**
42	-1820.17663948	76.6	-53	15	76	333
43	-1820.19293975	33.8	13	64	93	173

44	-1820.18856145	45.3	-34	72	130	249
45	-1820.18882024	44.6	-8	53	139	229
46	-1820.18413378	56.9	7	43	173	238
47	-1820.18936566	43.2	-2	43	178	275
48	-1820.16119065	117.1	-22	16	174	223
49	-1820.17430162	82.7	35	54	198	261
50	-1820.17498132	80.9	161	162	180	183
51	-1820.17908494	70.1	-8	161	203	216
52	-1820.17180368	89.3	39	54	250	306
53	-1820.17312581	85.8	161	161	167	220
**54**	**-1820.17324232**	**85.5**	**-87**	**128**	**145**	**189**
**55**	**-1820.17394115**	**83.6**	**-86**	**111**	**142**	**219**
**56**	**-1820.17488443**	**81.2**	**-83**	**130**	**142**	**175**
57	-1820.17775374	73.6	-15	181	196	214
**58**	**-1820.17237357**	**87.8**	**-75**	**137**	**161**	**195**
59	-1820.18208705	62.3	39	137	171	230
60	-1820.18003696	67.6	-47	149	188	206
61	-1820.16130150	116.8	35	157	168	294
62	-1820.18756699	47.9	8	67	78	250
63	-1820.18702493	49.3	-25	58	88	236
64	-1820.18600843	52.0	19	52	140	192
65	-1820.17873855	71.1	15	78	107	218
66	-1820.16632567	103.6	-82	91	111	305
**67**	**-1820.18281240**	**60.4**	**-80**	**65**	**131**	**158**
**68**	**-1820.17313341**	**85.8**	**-86**	**121**	**153**	**170**
**69**	**-1820.18984181**	**41.9**	**-91**	**67**	**122**	**123**
**70**	**-1820.17197831**	**88.8**	**-100**	**8**	**205**	**297**
**71**	**-1820.17411616**	**83.2**	**-99**	**-43**	**242**	**284**
**72**	**-1820.20361628**	**5.7**	**-96**	**74**	**272**	**278**
**73**	**-1820.18900967**	**44.1**	**-75**	**53**	**242**	**288**
**74**	**-1820.19053325**	**40.1**	**-78**	**45**	**254**	**268**
**75**	**-1820.20580174**	**0.0**	**-101**	**92**	**238**	**297**
**76**	**-1820.18736209**	**48.4**	**-119**	**119**	**211**	**246**
**77**	**-1820.16396082**	**109.9**	**-125**	**99**	**172**	**209**
**78**	**-1820.16317727**	**111.9**	**-114**	**102**	**169**	**176**
**79**	**-1820.18326768**	**59.2**	**-121**	**142**	**204**	**266**
**80**	**-1820.18303612**	**59.8**	**-101**	**188**	**224**	**255**
**81**	**-1820.15384000**	**136.4**	**-102**	**112**	**153**	**236**
**82**	**-1820.18731354**	**48.5**	**-166**	**-64**	**37**	**297**
83	-1820.15943315	121.7	-122	-83	12	178
**84**	**-1820.14904278**	**149.0**	**-110**	**-108**	**52**	**272**
**85**	**-1820.16673709**	**102.6**	**-74**	**113**	**142**	**197**
**86**	**-1820.17420455**	**83.0**	**-113**	**135**	**176**	**263**
87	-1820.17314002	85.8	124	136	288	289
88	-1820.16478638	107.7	-88	311	313	349
**89**	**-1820.18470047**	**55.4**	**-115**	**135**	**187**	**267**
**90**	**-1820.16704565**	**101.8**	**-47**	**123**	**137**	**195**
**91**	**-1820.18064786**	**66.0**	**-90**	**54**	**126**	**133**
**92**	**-1820.13688261**	**180.9**	**-92**	**-64**	**123**	**235**

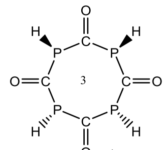
1
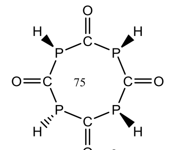
2
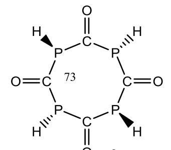
3
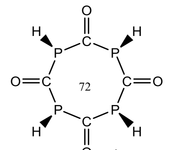
4
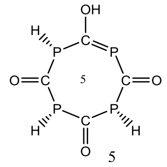
5
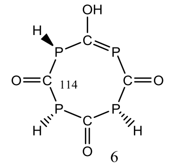
6
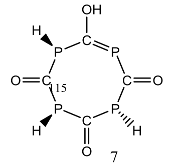
7
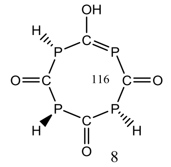
8
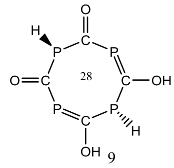
9
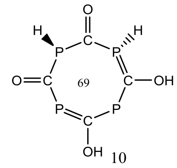
10
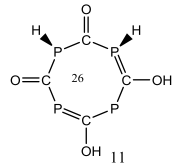
11
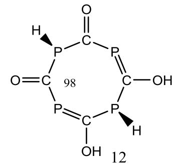
12
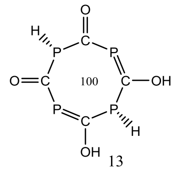
13
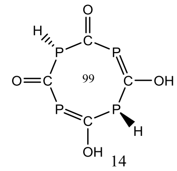
14
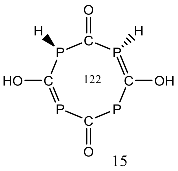
15
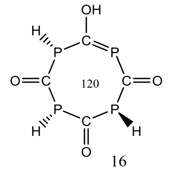
16
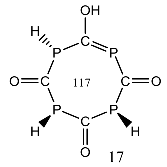
17
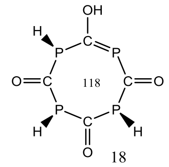
18
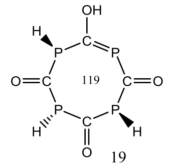
19
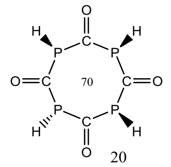
20
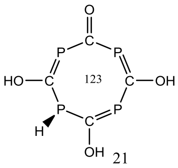
21
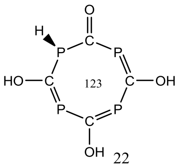
22
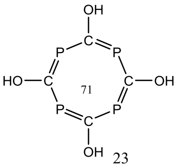
23
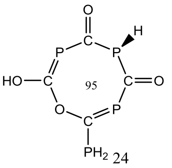
24
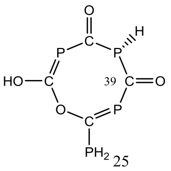
25
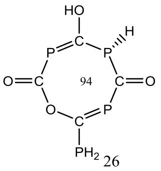
26
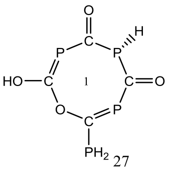
27
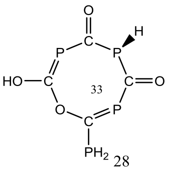
28
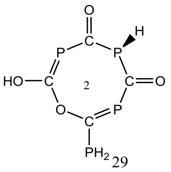
29
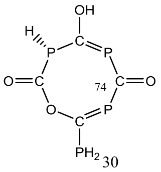
30
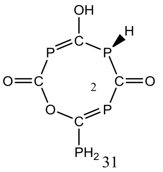
31
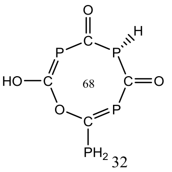
32
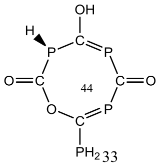
33
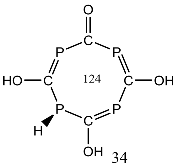
34

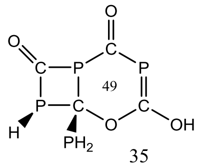
35
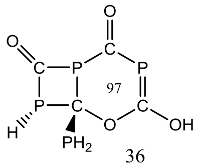
36
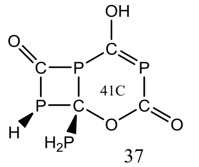
37
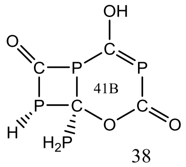
38
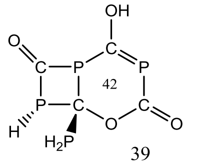
39
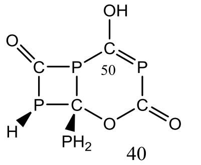
40
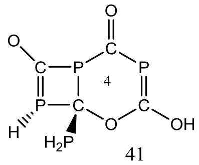
41
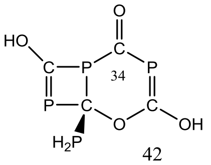
42
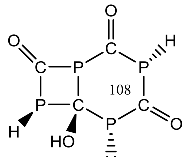
43
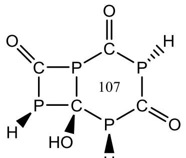
44
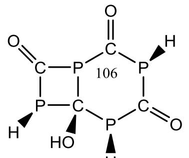
45
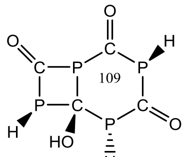
46
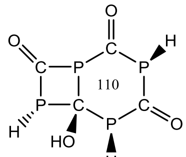
47
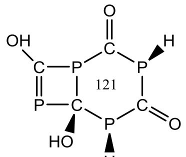
48
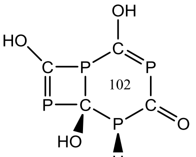
49
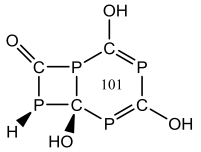
50
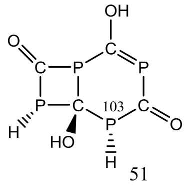
51
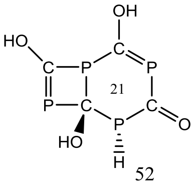
52
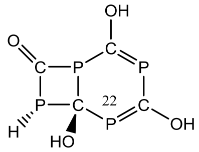
53
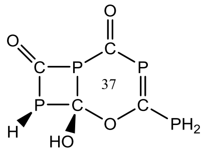
54
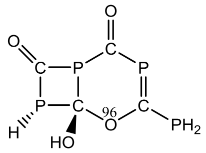
55
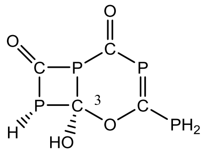
56
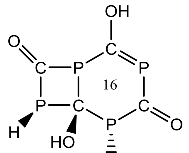
57
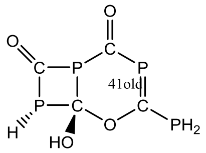
58
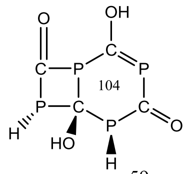
59
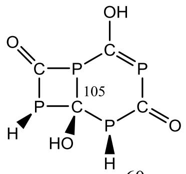
60
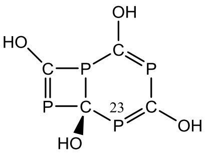
61
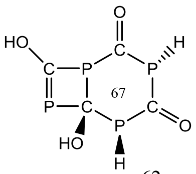
62
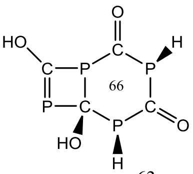
63
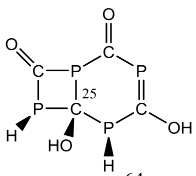
64
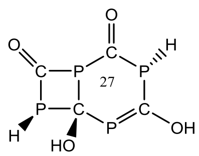
65
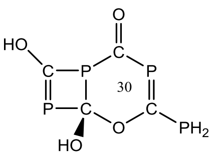
66
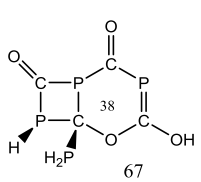
67
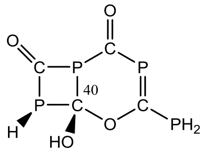
68
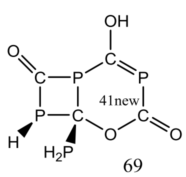
69

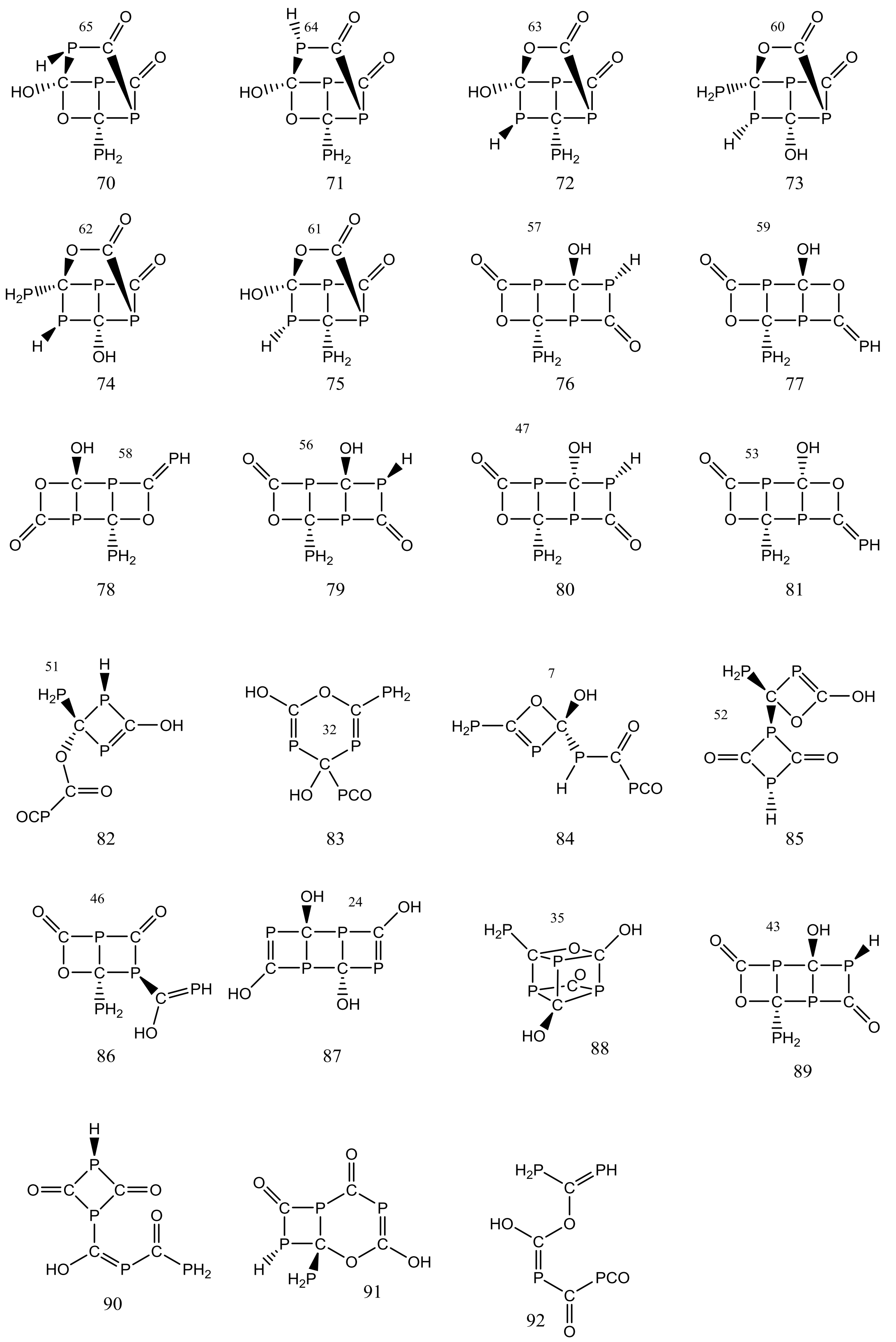

Figure S37. Computed isomers of $(HPCO)_4$.

4_01
0 1
C -0.08256500 -1.94015200 -0.18836200
O -0.48770900 -2.57873400 -1.12015000
P -1.29399300 -1.38015500 1.14039300
P 1.76130600 -1.73270300 0.09786800
C 2.09343600 0.10474400 -0.12207900
C -2.16125400 -0.04808200 0.07996800
O 2.99475500 0.43953500 -0.83735100
O -3.34719700 0.07395800 0.13508000
P 1.27635300 1.36298500 1.04296000
H 2.16012200 2.40748400 0.67822400
P -1.14943900 0.94417000 -1.16597600
C -0.11700900 2.04693900 -0.03969200
O -0.29432600 3.23157400 -0.04611200
H 2.15920400 -2.10730800 -1.20727100
H -0.40999400 -0.45298600 1.76642200
H -2.14256500 1.92699100 -1.39679700
4_02
0 1
C -1.93939700 -0.61812100 0.32426900
O -2.42249000 -0.95213100 1.37306000
P -1.16013300 -1.93163900 -0.76543300
P -2.15021900 1.14737600 -0.29669600
C -0.46022700 1.95943500 -0.08645500
C 0.66122700 -1.83169900 -0.21736900
O -0.40603100 3.01221900 0.48120800
O 1.45918400 -2.38939200 -0.91383400
P 1.03351600 1.31536900 -1.07492700
H 1.73762100 2.54070900 -0.98108300
P 1.28774200 -0.93393100 1.32617300
C 2.05136600 0.47159100 0.28017000
O 3.20732800 0.74036300 0.39951400
H -2.68486000 1.65397700 0.91200400
H 0.09628900 -0.21484200 1.60455400
H -0.89436600 -1.11317000 -1.89549500
4_03
0 1
C -1.39215800 -1.54724100 0.08475800
O -1.83031400 -2.41489900 0.78767900
P 0.09734400 -1.84599100 -1.04297100
H 0.19613500 -3.22516300 -0.73121500
P -2.43067600 0.00246700 -0.17071100
C -1.38896700 1.55009600 0.08425600
C 1.55652700 -1.36605800 0.06250000
O -1.82544200 2.41901500 0.78666300
O 2.61036800 -1.89091400 -0.14169800
P 0.10149800 1.84516800 -1.04319500
H 0.20283000 3.22437000 -0.73241000
P 1.43541800 -0.00164300 1.37802900
C 1.55934500 1.36309400 0.06306100
O 2.61403200 1.88658500 -0.14024000
H -3.02669200 0.00329200 1.11181200
H 0.01632000 -0.00016100 1.42784600
4_04
0 1
C -1.54188100 -1.43904400 0.00042800
O -2.41919100 -2.25826200 0.00104400
P -0.05932000 -1.70806300 -1.12549700
H -0.10788300 -3.11974800 -1.05061800
P -1.70828300 0.05931900 1.12539400
C -1.43904600 1.54187900 -0.00048800
C 1.43903600 -1.54187600 -0.00048500
O -2.25823300 2.41922500 -0.00115300
O 2.25820600 -2.41921800 -0.00116300
P 0.05939300 1.70832200 -1.12531700
H 0.10803100 3.11997900 -1.04998300
P 1.70804100 -0.05938600 1.12557200

H	3.11973100	-0.10802800	1.05077800
C	1.54199200	1.43893000	0.00049500
O	2.41946700	2.25796400	0.00097500
H	-3.11994400	0.10789600	1.05020800

4_05
0 1

C	-0.75799700	-1.32804100	0.80050000
O	-0.91888200	-0.84876500	1.88252000
P	0.93087500	-2.05837300	0.35895600
P	-2.28095900	-1.23258700	-0.42946400
C	-1.76086700	0.53530800	-0.56149400
C	2.00788900	-0.58521200	-0.23118000
O	-2.34480700	1.29880700	0.32135600
O	2.84458600	-0.85278300	-1.04529500
P	-0.25466400	0.82158500	-1.40020800
P	1.98923600	1.08728800	0.67046700
C	0.37739300	1.84233000	-0.00988600
O	-0.21845300	2.68125200	0.64125400
H	0.65440600	-2.46034200	-0.96981700
H	2.85109400	1.69277200	-0.28280600
H	-1.57228100	-1.63246600	-1.59632600
H	-1.79860600	2.11695600	0.46636100

4_06
0 1

C	1.93619000	0.46977200	0.46441100
O	2.32683700	0.48237300	1.59159200
P	1.12794600	2.02205300	-0.26723900
P	2.19430100	-1.09629100	-0.64182200
C	0.50971400	-1.61298400	-0.20749400
C	-0.76481100	1.79666000	-0.19057400
O	0.37033700	-2.15088200	1.01062300
O	-1.39667800	2.53042300	-0.89306300
P	-0.70292900	-1.12234100	-1.30978700
P	-1.61779500	0.68771600	1.09833200
C	-2.01690900	-0.83771900	0.02177900
O	-3.00358500	-1.48204300	0.22290600
H	1.18100900	1.65344400	-1.63406500
H	-2.89358100	1.28120800	0.92046200
H	-0.54486500	-2.42519300	1.15453300
H	2.87418600	-1.80986000	0.38160700

4_07
0 1

C	2.18199700	0.49214300	-0.02960900
O	3.12876800	1.08224200	0.39307400
P	0.81891100	1.42627700	-0.99421800
P	2.18159000	-1.41051800	-0.05265700
C	0.42640800	-1.53009600	0.38986000
C	-0.67512400	1.85312700	0.09864900
O	0.16531300	-1.49898100	1.69727400
O	-0.90459800	3.01859600	0.26412500
P	-0.57695500	-1.54360100	-1.00165500
P	-1.90287000	0.65449100	0.88194700
C	-2.14329600	-0.78180600	-0.31193500
O	-3.24581800	-1.12084600	-0.64560400
H	-3.06505000	1.38225600	0.53939600
H	-0.78748500	-1.46566700	1.86518100
H	2.73084100	-1.53921500	1.24944500
H	1.42233900	2.67460200	-0.70801100

4_08
0 1

C	2.25309600	0.48324400	-0.07020000
O	3.25466300	1.09844900	0.12198200
P	0.79602700	1.37367700	-0.94591800
P	2.24776100	-1.41939100	0.24203600
C	0.42686200	-1.45058400	0.44570100
C	-0.71219500	1.81878400	0.11962700
O	0.14180900	-1.30768000	1.74325100
O	-0.91769400	2.98476700	0.30678000

P	-0.53671500	-1.52447300	-0.96580700
P	-2.00377600	0.63372100	0.82336500
C	-2.15969600	-0.81869000	-0.35893300
O	-3.23413000	-1.19084400	-0.74454900
H	-3.14756500	1.36131300	0.42503500
H	-0.80362100	-1.14949400	1.89206900
H	1.37965300	2.64042400	-0.70215700
H	2.21650400	-1.67932100	-1.15696700

4_09
0 1

C	2.28911700	0.09124600	-0.11761600
C	-0.18113000	1.42798200	0.60291300
C	-2.45236000	-0.31204000	-0.19793700
C	-0.04182500	-1.32850400	-0.19614500
P	-1.35817100	-1.44756500	0.86476400
P	-1.87059600	1.53659500	-0.25163800
H	-1.43930400	1.43708600	-1.59888200
P	1.48884800	1.58160700	-0.34662300
P	1.70733200	-1.63481300	0.27980900
O	-0.13173400	1.53789400	1.79637400
O	-3.48551300	-0.59656600	-0.71320000
O	-0.22167300	-0.95159900	-1.47901100
H	0.59784600	-0.58713400	-1.84288400
O	3.60721900	-0.00072000	-0.40822700
H	3.96997600	0.88486000	-0.54471900
H	1.53107800	-1.45634100	1.67705100

4_10
0 1

C	-2.22077400	0.14550800	-0.01739300
C	0.39287900	1.69453300	-0.23652500
C	2.30784200	-0.54109400	-0.15585300
C	-0.19453200	-1.54363600	0.13624100
P	0.79822000	-1.08405400	-1.15604500
P	2.04808900	1.22134600	0.58541700
P	-1.12773400	1.35558900	0.86518700
P	-2.00035900	-1.54656800	-0.22874800
O	0.30201400	2.37112000	-1.21754900
O	3.36432100	-1.07925400	-0.06197500
O	0.25585500	-1.88052100	1.36166100
H	-0.49334500	-2.08360300	1.93346300
O	-3.40316100	0.65265800	-0.39840300
H	-3.49006400	1.55838500	-0.07867800
H	2.84195700	1.82786500	-0.42325700
H	-0.49649700	0.45876700	1.75260900

4_11
0 1

C	-2.15290600	0.20816100	0.09216300
C	0.38731300	1.72635400	-0.20098700
C	2.24167400	-0.56357400	-0.10747600
C	-0.23046700	-1.61601800	-0.02799400
P	0.78492300	-0.98519500	-1.23431700
P	2.22991400	1.35308400	0.17030200
H	2.07545600	1.18675300	1.57397600
P	-0.95520500	1.23457900	1.06396800
H	-0.26104100	0.19574800	1.71682600
P	-2.03520300	-1.45060900	-0.35344700
O	0.07942200	2.40519400	-1.13617000
O	3.07368100	-1.25284900	0.38585700
O	0.19248900	-2.15985200	1.12727700
H	-0.57265500	-2.38704600	1.66837700
O	-3.31293200	0.82199800	-0.18258600
H	-3.34314800	1.68118300	0.25396700

4_12
0 1

C	2.23369400	0.11459500	-0.12361400
C	-0.21973300	1.49788900	0.56645200
C	-2.42080800	-0.33851500	-0.17924100
C	-0.01849600	-1.41279600	-0.17144100

P	-1.28977600	-1.37043300	0.94637300
P	-1.85466100	1.50080200	-0.39673800
H	-1.33522000	1.24526700	-1.69139400
P	1.47955200	1.62877400	-0.32067900
P	1.66191900	-1.52444400	0.53340800
O	-0.25217000	1.68595600	1.75080600
O	-3.44257200	-0.68742900	-0.67812500
O	-0.20085800	-1.16811900	-1.48272600
H	0.64155000	-0.94262200	-1.89543300
O	3.53066600	-0.02857800	-0.48980500
H	3.91192500	0.83851600	-0.68010400
H	2.29777900	-2.24331600	-0.52267300

4_13
0 1

C	2.32114500	0.09216600	-0.04405900
C	-0.27114600	1.48451400	0.49681200
C	-2.46804100	-0.39166100	-0.11637800
C	0.01223700	-1.31437400	-0.23202000
P	-1.26492900	-1.47520400	0.86684800
P	-1.81320300	1.33924300	-0.63120200
P	1.51187700	1.57947700	-0.24093500
P	1.76385800	-1.66467000	0.17482000
O	-0.36287800	1.89945200	1.61646500
O	-3.61853500	-0.65108200	-0.29304000
O	-0.20089700	-0.88423400	-1.49419900
H	0.61760100	-0.55160600	-1.88865900
O	3.66608200	0.05698400	-0.20049100
H	4.00960700	0.95976300	-0.23926400
H	-2.63406500	1.96990300	0.34243400
H	1.60747000	-1.65358400	1.58651800

4_14
0 1

C	2.27887700	0.10107800	-0.06911900
C	-0.26521600	1.51847800	0.50448900
C	-2.44465900	-0.38780500	-0.14089400
C	0.01207600	-1.37612500	-0.21363100
P	-1.26377900	-1.44516200	0.89529800
P	-1.77046700	1.33140900	-0.67074400
P	1.51866700	1.61646000	-0.21504900
P	1.69669300	-1.57557800	0.45745200
O	-0.40262100	1.93462400	1.61859400
O	-3.58530400	-0.65510000	-0.36357100
O	-0.17715800	-1.02243000	-1.49994600
H	0.65789100	-0.73847000	-1.89361900
O	3.60169200	-0.00047100	-0.34390200
H	3.97697400	0.88044800	-0.47327000
H	2.30012600	-2.21958500	-0.66417200
H	-2.63104600	1.98393700	0.25223700

4_15
0 1

C	2.19993000	0.27106700	-0.27128200
O	2.88170600	0.58409900	-1.18802800
P	2.00620700	-1.58196200	0.24581500
P	1.26304700	1.63775200	0.76195300
C	-0.35998400	1.43153000	-0.05715100
C	0.17561500	-1.43199500	0.40433100
O	-0.30587400	1.86339500	-1.31422900
O	-0.16525100	-1.31689200	1.68107200
P	-1.91218900	1.06805600	0.76670500
P	-0.74136700	-1.21613000	-1.02201200
C	-2.33526800	-0.38449000	-0.32891100
O	-3.42355100	-0.85462100	-0.50273200
H	-1.20013300	1.85237600	-1.68479900
H	0.96212000	0.81558300	1.87063800
H	-1.00151000	-0.79231000	1.74072700
H	2.02607800	-2.01591300	-1.10406500

4_16
0 1

C	-0.75590800	-1.28786600	0.81160800
O	-0.88118500	-0.76167400	1.87870100
P	0.90590800	-2.06806700	0.38488800
P	-2.28834800	-1.21690100	-0.40231500
C	-1.74850700	0.54259600	-0.55582700
C	1.99068400	-0.61097300	-0.24007400
O	-2.31224300	1.30606100	0.34076200
O	2.75527600	-0.88107800	-1.11999900
P	-0.26476600	0.82096900	-1.43372200
P	2.08173100	1.12518900	0.55934100
C	0.42099400	1.82487500	-0.05177200
O	-0.15477800	2.65432900	0.62451500
H	-1.74266400	2.10391100	0.49697400
H	1.47698000	0.76725700	1.79437800
H	0.62389700	-2.50044900	-0.93278400
H	-1.57619700	-1.64144600	-1.55688900
4_17			
0 1			
C	1.16447900	1.44834400	0.41766700
O	1.20394200	1.81092800	1.55379100
P	-0.29339600	1.83333800	-0.77427700
P	2.66743400	0.43200400	-0.29128400
C	1.57864000	-1.04127400	-0.07880300
C	-1.83067300	1.07887400	0.08730700
O	1.69511100	-1.61759700	1.07801900
O	-2.59063600	1.78670700	0.68312800
P	0.29477900	-1.26633300	-1.23852600
P	-2.41914700	-0.70193500	-0.25684400
C	-0.91603000	-1.72856000	0.07020400
O	-0.73337500	-2.42575000	1.05468600
H	0.93184100	-2.23016900	1.23457100
H	-0.50284000	3.07385700	-0.10063800
H	2.29920800	0.57379700	-1.65620800
H	-3.05209700	-0.85218900	1.00100900
4_18			
0 1			
C	1.90279400	1.00345400	0.14552600
O	2.63019300	1.69004100	0.79173900
P	0.40161300	1.77773000	-0.77275600
P	2.47146400	-0.78610000	-0.24837100
C	0.87794300	-1.58871800	0.06146100
C	-1.18994900	1.55757100	0.27914700
O	0.66867000	-2.15297300	1.22377600
O	-1.41812800	2.30450200	1.18065500
P	-0.25464800	-1.25729400	-1.21744200
P	-2.59337700	0.40450400	-0.32650400
C	-1.67432100	-1.18497700	-0.04015100
O	-1.88354600	-1.94513100	0.88866600
H	-0.29017500	-2.40019600	1.28367900
H	0.66922900	3.00291300	-0.10748100
H	-3.27769600	0.41920500	0.91501400
H	3.04654900	-1.00001900	1.03029900
4_19			
0 1			
C	-0.72906200	-1.32999900	0.78772600
O	-0.91209200	-0.89382500	1.88428400
P	0.94532000	-2.06279500	0.33727600
P	-2.15616400	-1.23712900	-0.55768700
C	-1.76319700	0.54496200	-0.53752300
C	2.01582000	-0.57998800	-0.23951600
O	-2.33467300	1.28231000	0.37603500
O	2.78472300	-0.80866300	-1.12681500
P	-0.29471900	0.84669200	-1.43522000
P	2.06470700	1.13536600	0.59990700
C	0.40427800	1.81378400	-0.02620600
O	-0.17120500	2.65205500	0.63823100
H	-1.79239900	2.09867200	0.51777800
H	1.44421300	0.74196600	1.81659400

H -3.19384300 -1.17310200 0.41355400
H 0.65381100 -2.44710900 -0.99284400
4_20
0 1
C 1.54358600 1.35203800 -0.10834500
O 2.45511400 1.71304900 -0.79826000
P 1.75925900 -0.05264800 1.15142600
H 3.16321200 -0.12556200 0.97185400
P 0.00424400 2.41596600 -0.04454700
H 0.00499000 2.81176600 -1.40179500
C -1.53873700 1.35721600 -0.10840600
C 1.44294800 -1.50279800 -0.01785000
O -2.44894200 1.72088800 -0.79863400
O 2.18239400 -2.44359000 -0.01729300
P -1.75887000 -0.04631300 1.15191700
H -3.16317400 -0.11430000 0.97326500
P -0.00272000 -1.41474300 -1.22274700
H -0.00534400 -2.79919300 -1.52026000
C -1.44852600 -1.49746400 -0.01754700
O -2.19156700 -2.43542100 -0.01717600
4_21
0 1
4_22
0 1
C -0.75436500 -1.38949500 0.70259800
O -0.77791700 -1.16254000 1.87228300
P 0.78636300 -2.00415900 -0.22076500
P -2.44854200 -1.14635900 -0.28400600
C -1.79187200 0.56180300 -0.35075500
C 1.91823000 -0.72876800 -0.06132600
O -2.12145400 1.26029100 0.73363800
O 3.17537100 -1.08415200 -0.43081200
P -0.44839200 0.81740700 -1.40703600
P 1.86387300 0.97752900 0.63879000
C 0.54512400 1.71461200 -0.16983100
O 0.13445100 2.95853500 0.17762400
H -1.60766900 2.08169000 0.76091400
H -1.90396600 -1.49604200 -1.55327100
H 3.71908100 -0.29539800 -0.53092200
H 0.70669900 3.31748800 0.87256300
4_23
0 1
C 0.05418500 1.75148100 0.53793200
O -0.37950700 2.72538000 1.35863300
P -1.07484000 1.59965500 -0.90510500
P 1.62704400 1.11714400 0.78055900
C 1.78990000 -0.10775900 -0.56350000
C -1.75419000 0.06628500 -0.61132700
O 2.82502400 0.21931300 -1.35788900
O -2.67334100 -0.34491300 -1.51201400
P 0.98981200 -1.60346600 -0.81227500
P -1.63194700 -0.96788600 0.90420900
C -0.12476500 -1.72086100 0.63961400
O 0.23809600 -2.64664500 1.55019900
H 1.13808500 -2.93656700 1.36249100
H -2.98432800 -1.22254500 -1.26527400
H 0.29464000 2.92933900 2.02141900
H 3.02763300 -0.51188500 -1.95720100
4_24
0 1
C 2.51806600 -0.56250600 -0.03109400
C 0.03827900 -1.30978500 -0.00777800
C -1.91754900 -0.11741500 -0.01923500
C 0.52468200 1.55582300 0.28927200
P 2.12948000 1.22147800 -0.67052500
P 1.22742400 -1.23434100 1.19898900
P -1.31032700 1.44015600 -0.29854700
P -3.75051400 -0.24013200 -0.10279300

H	-3.78226300	-1.54751000	-0.66952900
H	-3.91008300	-0.72335200	1.22705800
O	-1.28957500	-1.30522700	0.30614600
O	0.27824300	-1.35147900	-1.31660800
O	3.54566700	-1.08314100	-0.32306900
O	0.62103800	2.18155600	1.31267300
H	-0.54198700	-1.20100700	-1.80519000
H	1.56954400	0.73406700	-1.87933700
4_25			
0 1			
C	-2.20977200	-0.40296900	0.02528600
C	0.14964400	-1.74831200	-0.03207200
C	1.68331000	0.20387900	-0.11764700
C	-0.34269400	1.99644600	-0.18364100
P	-1.37439400	0.88767100	1.04460200
P	-1.63554200	-1.95121400	-0.46414200
P	1.55102200	1.88833400	-0.06354300
P	3.35408600	-0.55525000	0.04255400
H	3.05570000	-1.37054500	1.16649600
H	3.14714200	-1.60798100	-0.88944400
O	0.52409300	-0.46567400	-0.40560100
O	0.88315900	-2.56959700	0.41729900
O	-3.51208200	-0.11370200	-0.11860700
O	-0.87710500	2.86357600	-0.80289600
H	-3.97267300	-0.84558100	-0.55061500
H	-2.48519700	1.76989400	1.00838000
4_26			
0 1			
C	-2.20977200	-0.40296900	0.02528600
C	0.14964400	-1.74831200	-0.03207200
C	1.68331000	0.20387900	-0.11764700
C	-0.34269400	1.99644600	-0.18364100
P	-1.37439400	0.88767100	1.04460200
P	-1.63554200	-1.95121400	-0.46414200
P	1.55102200	1.88833400	-0.06354300
P	3.35408600	-0.55525000	0.04255400
H	3.05570000	-1.37054500	1.16649600
H	3.14714200	-1.60798100	-0.88944400
O	0.52409300	-0.46567400	-0.40560100
O	0.88315900	-2.56959700	0.41729900
O	-3.51208200	-0.11370200	-0.11860700
O	-0.87710500	2.86357600	-0.80289600
H	-3.97267300	-0.84558100	-0.55061500
H	-2.48519700	1.76989400	1.00838000
4_27			
0 1			
C	-2.19354600	0.51709900	-0.14676100
C	1.53593100	-0.40432500	-0.08575400
C	-0.73884900	-1.88978800	-0.09632600
H	-0.68431000	-0.22289300	1.74600600
C	0.24052200	1.65003200	-0.09055900
P	-1.37030100	2.21434500	0.05381900
P	-1.77523800	-0.86059200	1.10831100
P	1.14507600	-2.01725700	0.22510300
P	3.27035900	0.20859300	-0.22085800
H	3.36658800	0.83193700	1.05368700
H	3.82732000	-1.02337300	0.21819900
O	0.50180500	0.41466000	-0.52605600
O	1.34352900	2.36195900	0.13924300
H	1.10536500	3.21089500	0.52817800
O	-1.25295900	-2.50211500	-0.98314800
O	-3.05834500	0.32412000	-0.94570000
4_28			
0 1			
C	2.51808100	-0.56256500	-0.03134200
C	0.03815400	-1.30958100	-0.00778500
C	-1.91767500	-0.11731500	-0.01923600
C	0.52483700	1.55567200	0.28945100

P 2.12964800 1.22139500 -0.67033000
H 1.56976700 0.73437200 -1.87936100
P 1.22749000 -1.23461600 1.19879900
P -1.31022000 1.44027400 -0.29855100
P -3.75065700 -0.24020500 -0.10299700
H -3.78197200 -1.54808900 -0.66858000
H -3.91052200 -0.72234700 1.22723500
O -1.28961400 -1.30495200 0.30648300
O 0.27784000 -1.35094700 -1.31667700
O 3.54560800 -1.08322100 -0.32344900
O 0.62126100 2.18107600 1.31307500
H -0.54233200 -1.19955900 -1.80510300
4_29
0 1
C -2.24365000 -0.35689200 -0.00970100
C 0.13326000 -1.60892600 0.09579600
C 1.72558900 0.19008800 -0.17051200
C -0.40295800 1.83295600 -0.13031500
P -1.53644900 0.92206500 1.11120600
P -1.62466000 -1.88905600 -0.45789700
P 1.48746700 1.81573200 0.19617600
P 3.43252400 -0.49942800 -0.16086100
H 3.11239700 -1.67674300 0.56694200
H 3.31758900 -1.14102500 -1.42734200
O 0.63758000 -0.54443400 -0.61705000
O 0.74360300 -2.23659600 0.90400900
O -3.48249000 0.02332700 -0.36549900
O -0.86401300 2.46951600 -1.02683000
H -0.52578500 0.12159000 1.69105300
H 0.13946607 -2.57602756 1.56839201
4_30
0 1
C 2.26967400 -0.17624800 -0.29311700
C -0.02564800 -1.46943700 0.18914700
C -1.82157100 0.08593500 0.04135400
C 0.27773500 1.70268400 0.40112700
P 1.72214100 1.36100000 -0.77980100
P 1.66858400 -1.38353900 0.99834400
P -1.47462900 1.66497000 -0.40997200
P -3.44992400 -0.67436800 -0.32542500
H -2.93250500 -1.92212400 -0.76928300
H -3.68090600 -1.13749200 1.00218100
O -0.86756900 -0.60438100 0.80413800
O -0.26789700 -2.10644100 -0.78663300
O 3.29094900 -0.67161800 -1.02241600
O 0.41003900 2.08867600 1.52485700
H 3.55570400 -1.52869400 -0.67003100
H 1.33980900 -0.44012400 1.99930800
4_31
0 1
C -2.24365000 -0.35689200 -0.00970100
C 0.13326000 -1.60892600 0.09579600
C 1.72558900 0.19008800 -0.17051200
C -0.40295800 1.83295600 -0.13031500
P -1.53644900 0.92206500 1.11120600
P -1.62466000 -1.88905600 -0.45789700
P 1.48746700 1.81573200 0.19617600
P 3.43252400 -0.49942800 -0.16086100
H 3.11239700 -1.67674300 0.56694200
H 3.31758900 -1.14102500 -1.42734200
O 0.63758000 -0.54443400 -0.61705000
O 0.74360300 -2.23659600 0.90400900
O -3.48249000 0.02332700 -0.36549900
O -0.86401300 2.46951600 -1.02683000
H -3.83829500 -0.58136700 -1.02867400
H -0.52578500 0.12159000 1.69105300
4_32
0 1

C -2.52404000 -0.59012000 -0.01914000
C -0.01446100 -1.29735500 0.14310000
C 1.94226200 -0.12099400 0.01333500
C -0.55478900 1.55260400 -0.27768700
P -2.02193200 1.05306100 0.83620600
P -1.18413200 -1.41024700 -1.07940400
P 1.32792600 1.45411900 0.13751100
P 3.77925100 -0.21864600 0.01448000
H 3.85189200 -1.43062400 0.76049400
H 3.87470800 -0.88797900 -1.23883500
O 1.31637000 -1.34143800 -0.14189500
O -0.28326700 -1.13785000 1.43784100
O -3.66277300 -0.94114300 0.00305400
O -0.73598200 2.29128000 -1.20803400
H 0.51791000 -0.88894700 1.91939300
H -2.92982000 1.80163800 0.04167500
4_33
0 1
C 2.04855500 -0.33354800 -0.15033200
C -0.01192100 -1.78876500 0.14882800
C -1.68832800 -0.02027600 -0.18202800
C 0.65110400 1.92425200 0.10187800
P 1.92205600 1.11133600 -1.02667000
P 0.99271600 -0.71882400 1.32487300
P -1.23279500 1.57930500 0.14155500
P -3.53964200 -0.16524300 -0.19995700
H -3.58351900 -0.88379700 -1.42884600
H -3.58073300 -1.35408000 0.58526300
O -1.08114500 -1.20678800 -0.47212200
O 0.28560000 -2.90522300 -0.14605300
O 2.93142000 -1.26059800 -0.55449200
O 0.94047800 2.92586600 0.69967400
H 1.75046500 -1.84625200 1.74589700
H 2.67148200 -2.13052800 -0.22544400
4_34
0 1
C -2.30480000 0.13447500 -0.06948900
O -3.64787600 0.14824400 -0.24317700
P -1.45356100 1.58698500 -0.33663200
P -1.84066000 -1.63664300 0.25939600
C -0.07964500 -1.38189700 -0.16904500
C 0.32641800 1.54282900 0.38518300
O 0.01883300 -1.26554800 -1.49424700
O 0.46071800 2.13100100 1.41998700
P 1.10119800 -1.15011700 1.03666500
P 1.95028200 1.26040800 -0.64578200
C 2.32748700 -0.28697500 -0.03845900
O 3.49724400 -0.92203500 -0.21930400
H 0.88966100 -0.89247800 -1.71256100
H -1.67498700 -1.52489400 1.66711400
H 4.12669300 -0.33655100 -0.65976700
H -3.94836400 1.06054900 -0.35469900
4_35
0 1
C 0.81731600 1.75439200 0.49774200
C -0.51487000 -0.88972800 0.10688700
C -1.94911900 1.08216800 -0.41511900
C 1.77179700 -0.55688500 -0.65619600
P 2.38545600 0.86271400 0.14432200
P -0.70779900 0.77349100 0.95591500
P -2.04403500 -0.74257100 -0.99647800
P -0.44910300 -2.44259400 1.16780900
H 0.71721800 -2.05621800 1.89050500
H -1.33278200 -1.94468800 2.16592800
O 0.56389300 -1.04683900 -0.84786400
O 2.62449800 -1.34489500 -1.30000500
O -2.51390900 2.04966200 -0.79885200
O 0.77583100 2.95426600 0.57006600

H -2.99301500 -1.08850100 0.00805900
H 3.48755600 -0.91344900 -1.32462500
4_36
0 1
C 0.63754700 -0.84910300 -0.08017900
C -1.77337700 -1.02413200 0.20438100
C -0.97923800 1.54261700 -0.57256600
C 1.52265900 1.38644300 0.46117000
P 0.65236200 0.75177500 -1.10621100
P -2.40627800 0.59587000 0.06589100
P 1.33968700 -0.16926600 1.55140300
O -1.06925900 2.73299200 -0.72848200
O 2.01844900 2.43568500 0.69226700
O -0.58856900 -1.57320600 0.06889800
H 0.08548300 0.33783200 2.01189100
P 1.74472500 -2.09591400 -0.94716500
H 2.89364200 -1.25615100 -0.92491100
H 2.14263200 -2.82011800 0.21316500
O -2.61269800 -1.99516400 0.54178100
H -3.50813500 -1.63592700 0.56852900
4_37
0 1
C 1.39369200 1.19527700 0.51156300
C -0.78652800 -0.69637600 0.14647300
C -1.43793100 1.60287900 -0.53278900
C 1.38003300 -1.23205200 -0.89272000
P 2.48217400 0.00308600 -0.05000600
P -0.35471600 0.96804800 0.88190600
P -2.32681500 -0.08419300 -0.77492200
P -1.08119200 -2.14673000 1.30654600
H 0.23555200 -2.14654400 1.85218400
H -1.61182800 -1.36006100 2.36842800
O 0.01870700 -1.16893100 -0.93422800
O 1.93219900 -2.06378700 -1.54936900
O -1.55489400 2.64478000 -1.07736400
O 1.81205700 2.40344700 0.91751100
H 2.74408300 2.52047700 0.68901900
H -3.11972500 0.14051800 0.38993600
4_38
0 1
C 1.33456100 1.08253700 -0.53934500
C -0.80934800 -0.71581300 -0.08143000
C 1.46215500 -1.61763000 0.25013700
P 2.42189700 -0.03115200 0.16045100
P -0.38329800 0.84483000 -1.08679100
P -2.25100800 -1.54326800 -0.96529200
P -1.11439700 0.13854900 1.58359600
O 0.13637100 -1.76842300 -0.01507000
O 2.09342000 -2.59247900 0.53305300
O 1.70116000 2.34650000 -0.80195700
H -2.83320300 -2.14341600 0.18754700
H 2.61633000 2.49199000 -0.52698500
H -3.10610100 -0.40499200 -0.90854100
C -1.08382300 1.67748300 0.50600000
O -1.38917200 2.80306800 0.70698400
H -2.53044800 0.05324400 1.67226900
4_39
0 1
C -1.46366100 1.09375800 -0.54050500
C 0.82693200 -0.63876900 -0.17204300
C 1.33346800 1.65479100 0.55382500
C -1.26297200 -1.25888900 0.95925300
P -2.45798500 -0.16363800 0.05306100
P 0.29641400 1.00522000 -0.90471300
P 2.43753800 0.10490100 0.51729800
P 1.12751200 -2.10298100 -1.31015600
H -0.18304600 -2.12289300 -1.87211200
H 1.67133800 -1.33027800 -2.37439000

O 0.09128500 -1.10507900 0.95575500
O -1.74360700 -2.07455700 1.68844100
O 1.32349200 2.63649700 1.21105700
O -1.97680800 2.25343200 -0.98028000
H 2.21692300 -0.28887700 1.86315700
H -2.91491500 2.30181000 -0.75197800
4_40
0 1
C -1.33302500 1.08290800 -0.48967600
C 0.80259200 -0.72871000 -0.05439500
C 1.06128600 1.68598700 0.38859200
C -1.47475000 -1.62692700 0.19658600
P -2.41953900 -0.02183800 0.22092400
P 0.34768000 0.75497400 -1.13261800
P 1.11367600 0.21948300 1.56621500
P 2.36161900 -1.59098400 -0.65451400
H 1.89952800 -1.84660600 -1.97673500
H 3.04094100 -0.39307100 -1.02280100
O -0.12574500 -1.78584400 0.02137800
O -2.12343300 -2.61499200 0.37065100
O 1.29732400 2.84268500 0.51912000
O -1.77360100 2.34202100 -0.65456800
H 2.53492600 0.12847100 1.61537900
H -1.05990900 2.93617800 -0.91526600
4_41
0 1
C 0.60753700 -0.86702400 -0.07824700
C -1.80743400 -0.97148200 0.19557000
C -0.94146000 1.57696400 -0.56192400
C 1.57132300 1.33531500 0.45948300
P 0.66643800 0.73743200 -1.10294400
P -2.38487900 0.67270300 0.09492700
P 1.14219200 -0.12168100 1.57105800
O -0.99566400 2.76925000 -0.72180600
O 2.20047900 2.32056200 0.65666400
O -0.64763800 -1.56104700 0.02502700
P 1.68265200 -2.14975200 -0.93211800
H 2.85802800 -1.34960200 -0.90928600
H 2.04899800 -2.88353900 0.23348600
O -2.67890100 -1.91634300 0.52423400
H -3.55988600 -1.52425400 0.56676400
H 2.45081500 -0.66516100 1.68295700
4_42
0 1
C 0.64709500 -0.88281600 -0.08161500
C -1.79535700 -0.97473200 0.28737600
C -1.01195000 1.51153000 -0.62977500
C 1.43126000 1.25426300 0.51610700
P 0.67191300 0.74246100 -1.06320500
P -2.44422200 0.62448000 0.07182700
P 1.40067100 -0.15622000 1.48500000
O -1.09316100 2.69140500 -0.86553100
O 1.92856800 2.46747100 0.68390400
H 2.30103400 2.56149200 1.56882200
O -0.61706600 -1.55165500 0.12135600
P 1.63880400 -2.20082900 -0.97871600
H 2.81635000 -1.41235400 -1.08375000
H 2.09723100 -2.89329900 0.18011600
O -2.67315200 -1.87491700 0.71184100
H -2.20991200 -2.71213100 0.84608900
4_43
0 1
C -0.79978600 -0.92210300 0.57516200
C 2.04839800 -0.79024500 -0.55136000
C 0.85251300 1.62247600 0.27984900
C -1.93074600 0.99358900 -0.55622800
P -0.76393500 0.92894400 0.89643100
P 2.36587600 0.57833200 0.68762900

P	-2.40563500	-0.85599700	-0.49753500
O	0.92164600	2.71801800	-0.19716500
O	-2.27656600	1.86782800	-1.27780900
O	2.86716900	-1.10067800	-1.37010200
O	-0.91889000	-1.56075300	1.82210800
H	-0.89211700	-2.51334900	1.68439000
P	0.47562100	-1.82125000	-0.46631900
H	-3.26944600	-0.64186900	0.61474700
H	3.25949100	1.29179100	-0.14895900
H	0.05401800	-1.40464400	-1.75405300

4_44
0 1

C	0.61989200	-1.15677300	-0.44183600
C	-2.01104600	-0.44163500	0.57018900
C	-0.37810700	1.70371800	-0.43145600
C	2.02888900	0.69504800	0.47617400
P	0.97045600	0.57871200	-1.10104900
P	-2.18236200	1.18371100	-0.34313800
P	1.41691500	-0.89733800	1.26747000
O	-0.08135000	2.83997800	-0.18630700
O	2.79656500	1.52092200	0.83671000
O	-2.51124700	-0.63111900	1.64079100
O	1.33947100	-1.99106500	-1.32981100
H	1.30272300	-2.89577500	-1.00115500
P	-1.12626800	-1.84602000	-0.35224500
H	-1.50413400	-1.40906700	-1.65402800
H	2.58716000	-1.68447300	1.07551500
H	-2.47214900	1.99147600	0.78459700

4_45
0 1

C	0.43081100	-1.24020500	-0.23248700
C	-2.32005200	-0.10428900	0.11135800
C	-0.11300400	1.70262000	-0.41384000
C	2.22645800	0.41793200	0.18955700
P	0.90008900	0.31865600	-1.19400400
P	-1.48803100	1.40805600	0.82711400
P	1.40978100	-0.82561300	1.33943100
O	0.20068300	2.82730200	-0.68933000
O	3.21200300	1.06682500	0.27642900
O	-3.48942400	-0.10834900	-0.16022700
O	1.01310700	-2.26672500	-1.01957600
H	1.00019500	-3.08901200	-0.51900100
P	-1.36606800	-1.71977700	0.03492800
H	-1.66758900	-1.92614600	-1.33529000
H	2.37844100	-1.86831500	1.28266300
H	-2.38381800	2.35486400	0.27370900

4_46
0 1

C	0.43246400	-1.26778100	-0.32682300
C	-2.23159000	-0.09864300	0.28965300
C	-0.05337500	1.68350800	-0.65624700
C	2.07765600	0.40104800	0.51711100
P	1.13185900	0.28891000	-1.14771900
P	-1.34701000	1.50446000	0.67923600
P	1.11799100	-0.94580600	1.42229300
O	0.17890500	2.76765100	-1.11595700
O	2.97185100	1.10122600	0.85169200
O	-3.38029200	-0.21777000	0.62117000
O	1.10901300	-2.30628500	-1.01856700
H	0.92482900	-3.14416800	-0.58378800
P	-1.40272500	-1.50909600	-0.62012500
H	2.15487800	-1.92217300	1.40847000
H	-1.65906700	-2.44716800	0.41774800
H	-2.30911500	2.36911300	0.10341600

4_47
0 1

C	0.44857300	-1.24938500	-0.26355600
C	-2.30281300	-0.11688500	0.13606100

C	-0.13427000	1.70372500	-0.44125400
C	2.17281600	0.48136000	0.21638000
P	0.89904800	0.32723900	-1.20517500
P	-1.47882000	1.41002400	0.82916000
P	1.63615800	-1.05892900	1.20210100
O	0.16218400	2.82636800	-0.74169500
O	3.00481700	1.29347800	0.42755700
O	-3.47044800	-0.13385400	-0.14195600
O	0.95725700	-2.26981400	-1.11770800
H	1.07840100	-3.07579600	-0.60402700
P	-1.33120400	-1.72011400	0.12083200
H	-2.39580300	2.34911700	0.29888800
H	-1.68235300	-2.02571300	-1.21775000
H	0.78570800	-0.26321500	2.02376400

4_48
0 1

C	-1.53321900	-0.98437400	0.79078900
O	-1.88639100	-0.80207300	1.90833700
P	-0.08054100	-2.13362800	0.34841500
P	-2.45357300	-0.01051900	-0.64467100
C	-1.05905400	1.16775500	-0.62518900
C	1.11732700	-0.91347000	-0.41087900
O	-1.26279100	2.31490100	-0.05849000
O	1.83236100	-1.70669300	-1.32011200
P	0.57342100	0.75884900	-1.18803400
P	2.14799700	-0.02994900	0.92620300
C	1.34104700	1.44506700	0.41507900
O	1.08701800	2.56589300	0.84234500
H	-0.37081400	2.69303700	0.28378400
H	-0.65665300	-2.55579200	-0.88555200
H	-1.88412100	-0.77962400	-1.69378200
H	2.74386200	-1.39502400	-1.32857500

4_49
0 1

C	-0.39165500	-1.26116100	0.33157500
C	2.27560700	-0.35135000	-0.14253600
C	0.39414700	1.65282600	0.12815100
C	-2.14321200	0.30028600	-0.11687800
P	-0.71715700	0.46541300	0.98393000
P	2.07678400	1.51485800	-0.13173100
P	-2.13536600	-1.36109200	-0.47435500
O	-0.15519600	2.86989300	-0.04533800
O	-2.93102700	1.34132800	-0.40557400
O	3.32372300	-0.86216700	0.14138400
O	-0.15383800	-2.09288400	1.42844100
H	0.07731600	-2.96911900	1.10837700
P	0.89778500	-1.31514300	-1.02537300
H	-1.11028100	2.84791700	0.09304700
H	-3.65742700	1.07401900	-0.98030400
H	1.38109100	-2.60633500	-0.66134200

4_50
0 1

C	-0.29320700	-1.32489700	0.27557300
C	2.17112900	0.01759400	-0.13569700
C	-0.07979000	1.60400100	0.35745800
C	-2.16490400	0.21913100	-0.28755000
P	-0.92757100	0.20777600	1.16586500
P	1.47692600	1.69191600	-0.33405100
P	-1.35665600	-1.16434000	-1.27961100
O	-0.78553500	2.74858900	0.36397100
O	-3.03404900	0.99980500	-0.52152200
O	3.51707000	0.03784900	-0.31198800
H	3.82962700	0.94951500	-0.34409500
O	-0.76918200	-2.45323200	0.99220900
H	-0.13091900	-2.66281600	1.68463600
P	1.54916300	-1.56543800	0.03383900
H	-1.73812000	2.56623500	0.36958000
H	-2.31431900	-2.16070900	-0.94080900

4_51
0 1

C	0.38042400	-1.30122300	-0.43732900
C	-2.11491600	-0.05051700	0.38250200
C	-0.05078500	1.64918600	-0.50115200
C	1.97733500	0.28103900	0.65733700
P	1.09228700	0.32583200	-1.04681900
P	-1.63076200	1.72900900	0.15228600
P	1.18145400	-1.32923700	1.28699900
O	0.53781900	2.85600100	-0.64283300
O	2.73747400	1.04252100	1.14708500
O	-3.04013400	-0.26484300	1.12775700
O	1.05594300	-2.24018900	-1.26538900
H	0.92225000	-3.12321600	-0.90676400
P	-1.46352300	-1.47564300	-0.65302800
H	1.39611800	2.78203700	-1.07801500
H	-1.67445700	-2.43820400	0.37327600
H	0.18308300	-0.58887300	1.97882800

4_52
0 1

C	-1.29111500	0.41764200	-0.68579300
C	1.06915000	1.53835700	0.62360500
C	1.48638600	-0.90152200	-0.60714000
C	-0.94279500	-1.34595100	0.88760700
P	-0.33847800	-1.19419500	-0.81520100
P	2.40206200	0.31882600	0.14519700
P	-2.07475600	-0.07788500	0.98141300
O	2.18627600	-1.95600900	-1.07540600
O	-0.55277800	-2.31662900	1.70348400
O	1.23080400	2.20979900	1.60920600
O	-2.17457300	0.46172900	-1.78349200
H	-2.78437800	1.19521800	-1.66122700
P	-0.26729400	1.98735000	-0.62148700
H	1.58662800	-2.56871100	-1.52094200
H	-1.00907500	-2.24771700	2.55007800
H	-1.06377900	2.66749200	0.34327100

4_53
0 1

C	0.28671400	-1.32129900	-0.29481200
C	-2.16330900	0.03324800	0.14734000
C	0.10464800	1.58733900	-0.38212500
C	2.14907900	0.19625600	0.32801300
P	0.96724100	0.18584000	-1.17348500
P	-1.45319100	1.70442700	0.29707100
P	1.41140300	-1.29929000	1.23953700
O	0.81806300	2.72767800	-0.41106500
O	2.96378700	1.00984800	0.62955100
O	-3.50450500	0.06209500	0.36621700
H	-3.81880500	0.97339100	0.35118000
O	0.74110600	-2.36920200	-1.14725500
H	0.43753600	-3.21232600	-0.79343400
P	-1.55468400	-1.54489200	-0.06544800
H	1.76841400	2.53944000	-0.35292700
H	0.64093800	-0.40838400	2.03996900

4_54
0 1

C	-0.32369000	-1.16699600	0.43122900
C	1.75814800	0.04517000	-0.00480900
C	-0.47732800	1.72225700	0.27557900
C	-2.38034800	-0.10684100	-0.38197400
P	-1.32217100	0.27712100	1.15297100
P	1.30298500	1.67894100	-0.24003600
P	-1.14679000	-1.20940800	-1.29153100
O	-1.11452600	2.73101200	0.12327100
O	-3.46358500	0.26772000	-0.68143800
O	1.09959600	-1.05896700	0.34997000
H	-1.84079400	-2.44155900	-1.12863900
O	-0.57517100	-2.24043400	1.25177200

H	-0.19970300	-3.03541600	0.85801300
P	3.54363600	-0.39395200	-0.14497300
H	3.41061800	-1.16637400	-1.33392200
H	3.93376800	0.79661900	-0.81065300

4_55
0 1

C	-0.31881200	-1.17972300	0.44471400
C	1.74766700	0.06104000	-0.01726500
C	-0.50022100	1.71682000	0.29647300
C	-2.36009300	-0.11106000	-0.40384500
P	-1.33453600	0.25721300	1.15440200
P	1.28066700	1.69642000	-0.22034200
P	-1.23442200	-1.42851800	-1.20999100
O	-1.14540400	2.72186500	0.15670600
O	-3.37437100	0.36447400	-0.78365700
O	1.09668100	-1.05552800	0.30931400
O	-0.53772900	-2.23464500	1.30120300
H	-0.25204000	-3.05297600	0.87946700
P	3.53728600	-0.36186000	-0.16300400
H	3.41172700	-1.12596200	-1.35817500
H	3.91585800	0.83740700	-0.81999800
H	-0.53512700	-0.39911100	-1.90624500

4_56
0 1

C	2.40566700	-0.10710400	-0.35716800
C	-1.77028700	0.03378200	0.03545800
C	0.45070700	1.74713700	0.22806100
C	0.33634300	-1.15769700	0.41778600
P	1.18442400	-1.21095900	-1.27054500
P	1.29622300	0.32513200	1.13350100
P	-1.36556600	1.68784000	-0.14186900
P	-3.48947100	-0.48048300	-0.39958500
H	-3.81809600	-0.96930100	0.89647300
H	-4.08239400	0.79422800	-0.20412100
O	-1.08906800	-1.07227600	0.34330900
O	0.61262400	-2.31057100	1.11553100
H	0.28404500	-2.21390100	2.01692000
O	1.08101900	2.74495100	-0.00072700
O	3.50088900	0.25339300	-0.62859900
H	1.85401400	-2.45466500	-1.11271900

4_57
0 1

C	0.32769300	-1.35454000	-0.32511600
C	-2.24390600	0.07662000	0.27042600
C	0.01703600	1.60736700	-0.43322200
C	2.06938400	0.25615100	0.45970700
P	0.99503800	0.21542100	-1.11767500
P	-1.57009700	1.79938100	0.17733400
P	1.17864900	-1.13085500	1.37255300
O	0.72290100	2.75481100	-0.49915800
O	2.93303000	1.01527700	0.76243700
O	-3.33172200	-0.03516300	0.78285100
O	0.93745100	-2.35878300	-1.12035100
H	0.61973800	-3.22077500	-0.83607900
P	-1.52967900	-1.52339400	-0.42185900
H	1.67565900	2.59068300	-0.53809300
H	-1.73142900	-2.21132200	0.80420700
H	2.21285600	-2.08960000	1.17767500

4_58
0 1

C	-0.31175000	1.84316300	0.14189200
C	-0.33419000	-1.06360400	0.45024400
C	-2.44975200	-0.04820400	-0.34382300
C	1.72582700	0.03469500	-0.21935800
P	1.54737500	1.69990800	0.08521100
P	-1.26167500	0.49069800	1.01166000
P	-1.76778500	-1.80666300	-0.57218700
O	0.78657000	-0.91948600	-0.42595800

O	-3.33504000	0.52015800	-0.89024400
O	-0.87161600	2.82748900	-0.25668900
H	-1.21989900	-1.50544600	-1.84865400
O	0.04441000	-1.76340400	1.57498400
H	0.36659700	-2.63245300	1.31183600
P	3.39365700	-0.72806600	-0.34781100
H	3.28889700	-1.08559000	-1.72141800
H	4.11542600	0.47097600	-0.58533400

4_59
0 1

C	-0.48914200	-1.26212400	0.29161800
C	2.28005100	-0.33574900	-0.09723900
C	0.37134100	1.63172500	0.16325400
C	-2.12146800	0.57740900	-0.09765700
P	-0.67578200	0.44175000	1.08542600
P	2.01338700	1.50774200	-0.27914700
P	-2.18076000	-1.22563900	-0.61706500
O	-0.21200900	2.83564600	0.05567100
O	-2.70151700	1.55759900	-0.46982500
O	3.35928900	-0.76489300	0.20887400
O	-0.50799600	-2.14465100	1.38870700
H	-0.53046700	-3.04959700	1.06422000
P	0.99049500	-1.50163700	-0.85565800
H	-1.18049500	2.75103800	-0.00630300
H	-1.71497800	-0.92768800	-1.92722300
H	1.46900400	-2.64415200	-0.16130400

4_60
0 1

C	-0.34139900	-1.33026300	0.24518500
C	2.33999900	0.04876100	-0.14326000
C	0.05511700	1.60401900	0.26416500
C	-2.17579400	0.30743300	-0.16922000
P	-0.81315200	0.24810100	1.15358400
P	1.62539000	1.74495600	-0.39484300
P	-1.52790900	-1.09521700	-1.23464900
O	-0.66366600	2.74136000	0.26500100
O	-3.04722000	1.10941700	-0.31599200
O	3.53928700	-0.04130900	-0.06418900
O	-0.75121000	-2.34841400	1.13805800
H	-0.59373600	-3.20443200	0.72630300
P	1.44759900	-1.61854800	-0.23600300
H	-1.61480200	2.56771200	0.34497400
H	1.84785800	-1.96839000	1.08015300
H	-2.50330800	-2.05239000	-0.83702600

4_61
0 1

C	-0.56649800	-1.20093800	0.57011100
C	2.06990600	-0.41420200	-0.30297300
C	0.33813800	1.67894900	0.42345900
C	-1.94358400	0.36054900	-0.60365400
P	-1.02334400	0.56815900	0.94925600
P	1.93434800	1.38377100	-0.07956700
P	-1.83836200	-1.32082900	-0.85751000
O	-0.00152500	2.97957700	0.52278900
O	-2.54859000	1.37008400	-1.22405900
O	3.30755700	-0.73219500	-0.77404500
H	3.81341300	0.07274900	-0.93511700
O	-0.86522800	-1.96226100	1.71786600
H	-0.45925200	-2.83048600	1.62140800
P	1.05587300	-1.77447100	-0.15353500
H	-0.83553800	3.06307800	1.00386300
H	-2.97183300	1.05742300	-2.03188500

4_62
0 1

C	-0.48215400	-1.10829200	0.40438300
C	2.29277100	-0.50770500	-0.29553600
C	0.44678400	1.68733100	0.00461800
C	-2.25297100	0.42279900	-0.10789900

P	-0.82363300	0.67545900	0.95882400
P	2.19230200	1.19916900	0.54157600
H	2.86157800	1.87007400	-0.51344800
P	0.71228500	-1.19806500	-1.02654900
P	-2.22648600	-1.27052600	-0.33967700
O	3.36094100	-1.02916400	-0.44579600
O	0.19348000	2.62984400	-0.68998000
O	-3.08495000	1.39238200	-0.45923300
H	-3.80633400	1.03806700	-0.99189600
O	-0.29036100	-2.04308700	1.42104300
H	0.61662000	-1.99267100	1.73606600
H	1.05164600	-2.57061000	-0.88499100

4_63
0 1

C	0.36779600	-1.15207500	-0.22618400
C	-2.17993200	-0.46970300	0.15913500
C	-0.35934600	1.73167900	0.02296000
C	2.23482900	0.34455200	-0.09481400
P	0.69044800	0.55681200	-1.00659500
P	-2.25363600	1.40270000	-0.05431000
P	-0.75533100	-1.00791300	1.27267600
P	2.16939400	-1.30275300	0.35282900
O	-2.94467200	-1.22222200	-0.38300100
O	0.07457600	2.68145200	0.60433000
O	3.13572300	1.30674300	0.04144700
H	3.89647900	0.98575500	0.53913400
O	0.02609800	-2.16831500	-1.10810000
H	-0.92987200	-2.17116600	-1.24081900
H	-1.11329800	-2.38500000	1.30104300
H	-2.33030200	1.32976000	-1.47234400

4_64
0 1

C	-0.57513800	-1.03762400	0.44960000
C	2.11342700	-0.45976500	-0.28591400
C	0.53680700	1.75422400	0.13209600
C	-2.21989500	0.66478400	-0.28440900
P	-0.82147300	0.74998300	0.95453000
P	2.30713700	1.14220900	0.27299700
P	-2.32241200	-1.24552300	-0.32810800
O	0.29543300	2.85511600	-0.27747000
O	-2.83930900	1.49592200	-0.85867600
O	3.15680300	-1.29492800	-0.40959800
H	3.97259600	-0.84556700	-0.15035500
H	-2.99557800	-1.26493600	0.92882000
O	-0.27206400	-1.80782500	1.57285000
H	-0.13351900	-2.71876400	1.29920600
P	0.59268300	-1.18935800	-1.00530900
H	0.95938300	-2.54638200	-0.77440500

4_65
0 1

C	-0.68195500	-0.90553600	0.57955100
C	1.94204400	-0.72371900	-0.45502100
C	0.70178700	1.70170000	0.21796000
C	-2.09308800	0.79945600	-0.56031400
P	-0.86346100	0.93770300	0.84355300
P	2.21980000	0.65063700	0.72063900
P	-2.37995800	-1.07544900	-0.33135100
O	0.80409000	2.77370500	-0.29549800
O	-2.55218600	1.58695600	-1.31665800
O	3.00436500	-1.12186900	-1.17999700
H	3.70622200	-0.46391900	-1.14535500
H	-3.17101800	-0.82198100	0.82872900
O	-0.59397800	-1.54888600	1.82527400
H	-0.30529700	-2.45458700	1.67487800
P	0.49964800	-1.60420900	-0.71188000
H	3.11859800	1.35960200	-0.13068100

4_66
0 1

C	-0.30532300	-1.23069600	0.49521000
C	1.74395100	0.07703000	-0.00607300
C	-0.47609300	1.75075000	0.36283600
C	-2.15586000	-0.19729500	-0.50116600
P	-1.36629200	0.24489500	1.08517900
P	1.30996100	1.73163800	-0.11343700
P	-1.25534100	-1.51683000	-1.11016100
O	-1.11789700	2.76395900	0.26277700
O	-3.22362600	0.44948200	-0.93590600
H	-3.50962100	0.09633800	-1.78693200
O	-0.42167900	-2.33602200	1.30387800
H	-0.02485900	-2.13990700	2.15963500
O	1.11181900	-1.04793700	0.31223900
P	3.44618300	-0.38937200	-0.55389400
H	4.03456800	0.88379100	-0.33522700
H	3.85327000	-0.92975700	0.69847400

4_67
0 1

C	-0.49506400	-0.90166300	0.10780300
C	1.79785500	-0.49501800	-0.64996000
C	0.77858200	1.76628600	0.48024100
C	-1.97573600	1.03077100	-0.41026500
P	-0.72742600	0.75524300	0.96097100
P	2.38189800	0.94517800	0.11769100
P	-2.03256200	-0.80045300	-0.98110500
O	0.68837000	2.96459100	0.54578400
O	-2.55881900	1.98573400	-0.79770500
O	2.71430200	-1.23846000	-1.25363000
H	2.28470100	-2.03305000	-1.59665400
H	-2.96743900	-1.15519900	0.03082800
P	-0.22994900	-2.33253800	1.28805000
H	-1.38066400	-2.07841100	2.08132300
H	-0.86172600	-3.33829600	0.50055300
O	0.58263400	-1.00046000	-0.86533200

4_68
0 1

C	-0.38820700	-1.05106700	0.49750500
C	1.72492900	-0.02774500	-0.16102900
C	-0.23690100	1.87471600	0.16518200
C	-2.41468100	0.01386400	-0.45493900
P	-1.28788900	0.54576400	0.94575400
P	1.61191000	1.63766000	0.18591000
P	-1.73097300	-1.75703000	-0.65985200
O	-0.73110300	2.89504300	-0.23118900
O	-3.27943800	0.57868500	-1.03623500
H	-2.54718200	-2.25384900	0.39631700
O	0.77116500	-0.97046400	-0.34043400
O	-0.07056500	-1.73587700	1.64922900
H	0.27549700	-2.60185100	1.40771300
P	3.34155400	-0.75185200	-0.65562000
H	4.13695400	0.31359500	-0.15793400
H	3.48439600	-1.57374200	0.49975300

4_69
0 1

C	-0.79237600	-0.69696900	0.14724500
C	1.37157200	-1.23084700	-0.90367500
C	1.39181600	1.18507000	0.51817100
C	-1.43557000	1.59724000	-0.54760300
P	-0.36123000	0.97116100	0.88105800
P	2.47612700	-0.00555200	-0.05036500
P	-2.33179500	-0.08817200	-0.77029200
O	1.81296800	2.39086600	0.93005200
O	-1.53665000	2.63423000	-1.10459500
O	1.91977000	-2.05121000	-1.57733700
H	-3.11675400	0.15751800	0.39367000
P	-0.90056300	-2.06833100	1.42077700
H	-1.91869600	-1.45964700	2.20633300
H	-1.79945400	-2.90190500	0.69830200

O 0.00878700 -1.16967200 -0.93596100
H 2.74518200 2.50676000 0.70192100
4_70
0 1
C -0.31310300 -0.91359800 1.56584000
C -0.35436700 1.45726800 -0.02885900
C 1.17858000 -0.04330100 -0.24795500
C -1.46008000 -0.94993400 -0.93437000
P 0.13969500 -1.65975900 -0.12264500
P 0.66294600 0.71693200 1.42232600
O -2.06560100 -1.59882500 -1.73069500
O -0.95945800 -1.34706500 2.46303500
P 2.94633000 -0.38719900 -0.64310800
H 2.74713500 -0.62184600 -2.03252000
H 3.32488700 0.97333000 -0.81138900
O -0.37567900 2.82590800 0.06584600
H -0.70044700 3.19136400 -0.76502800
O 0.56594300 0.99329700 -1.01937600
P -2.06987400 0.72417700 -0.33013900
H -2.18583800 1.25577300 -1.64600700
4_71
0 1
C -0.43761600 -1.09285000 1.37144000
C -0.17791400 1.50457200 0.11805400
C 1.20066700 -0.12275000 -0.25841300
C -1.62032900 -0.71833700 -0.90768500
P 0.00691500 -1.60645900 -0.41469400
P 0.67634600 0.44121600 1.48155900
O -2.54089800 -1.38613200 -1.27103000
O -1.17048900 -1.57736500 2.16776700
P 2.94630100 -0.59504300 -0.62332200
H 2.81262900 -0.58090400 -2.04010000
H 3.47096300 0.72413900 -0.54558200
O -0.02333900 2.82526200 0.45470100
H -0.31946400 3.37196000 -0.28296400
O 0.73867500 1.06928100 -0.90156400
P -1.83899600 1.13631000 -0.65529200
H -2.64307500 0.97227600 0.50550000
4_72
0 1
C -0.33470200 -1.43124700 1.10220700
C -0.91599500 1.25665800 0.35324000
C 1.13924100 0.10263500 -0.22414900
C -1.55134100 -0.63768400 -1.04021300
P 0.15001300 -1.42277800 -0.75079800
P 0.32063200 0.31010500 1.49314600
P 0.52212200 1.77628500 -0.78980200
O -1.88920800 0.47436100 -0.34264700
O -2.34824700 -1.16957600 -1.74578900
O -0.84393800 -2.25172800 1.78448300
H -0.04446900 1.45954600 -2.06222800
P 2.95328300 -0.30792700 -0.22542500
H 3.18200300 -0.11664400 -1.61798500
H 3.41896100 0.98744400 0.13525000
O -1.60666500 2.19728900 1.05635900
H -2.26600800 2.58945500 0.47238300
4_73
0 1
C 1.35747300 -0.54176100 1.27762200
C -1.17359500 -0.12048200 0.00840000
C 0.71294100 1.35954300 -0.21677400
C 0.58425600 -1.45434100 -1.00450400
P 1.85451100 -0.12150200 -0.53167300
P -0.08676700 0.69350400 1.36790800
P -0.87150000 1.28341500 -1.25024800
P -2.96285800 -0.49482900 0.38543600
H -2.69975500 -1.36163400 1.48386400
H -3.15884800 0.64622000 1.21202700

O -0.69218200 -1.34343000 -0.55306800
O 0.93620800 -2.43321900 -1.57855700
O 1.51063600 2.49597900 -0.22351000
O 1.81144700 -1.29934500 2.06439800
H -1.56227100 2.25338000 -0.45542700
H 1.00476100 3.23557800 0.12563600
4_74
0 1
C 1.32426400 -0.56178200 1.28898000
C -1.18107400 -0.09768900 0.00062600
C 0.75168400 1.34095500 -0.23162900
C 0.57128300 -1.45594700 -1.00756300
P 1.86460400 -0.16682700 -0.50290400
P -0.08824200 0.70460700 1.38022000
P -0.89438100 1.40385800 -1.11701600
P -2.97466700 -0.45856000 0.36914800
H -2.74179000 -1.29065700 1.50111900
H -3.17911700 0.71380500 1.14683600
O -0.70719300 -1.31630100 -0.57528900
O 0.90053900 -2.43319300 -1.60000000
O 1.58046200 2.45504300 -0.23151300
O 1.73899600 -1.32952400 2.08824600
H -0.65222400 0.73967100 -2.35551700
H 1.06405400 3.22960300 0.01182200
4_75
0 1
C -0.39296800 -1.43134200 1.10205100
C -0.86799200 1.28294800 0.34940200
C 1.13988300 0.05771300 -0.20823900
C -1.57920600 -0.58849200 -1.03390800
P 0.10388600 -1.42849000 -0.75941900
P 0.31918600 0.29332300 1.48235000
P 0.52015400 1.69013200 -0.91256700
O -1.87696300 0.53644200 -0.33523100
O -2.40121700 -1.10532100 -1.72020900
O -0.92903600 -2.24315200 1.77318900
H 1.22454200 2.49548500 0.03120600
P 2.94096400 -0.39107400 -0.22607300
H 3.17996700 -0.13598200 -1.60668000
H 3.43313700 0.87605000 0.19825200
O -1.50811400 2.27800000 1.02231100
H -2.17617100 2.65336600 0.43653400
4_76
0 1
C 2.17718300 -0.85005900 -0.07680600
C 0.79984800 0.81673700 0.11260600
C -0.60291900 -1.28531500 0.06978200
P 0.65522900 -0.58714500 -1.16806900
P -0.54425900 0.22528800 1.29583400
P 1.01746200 2.63544700 -0.18524700
H 1.83470900 2.48143500 -1.34190800
H -0.18366600 2.79634300 -0.92635900
O 2.04216300 0.28814600 0.66715300
O -0.26992300 -2.56863200 0.45701500
O 3.04635000 -1.64271100 0.01680300
H -0.96500800 -2.92553200 1.01852300
P -2.38754300 -0.99401100 -0.46690700
C -2.02144300 0.71412500 0.20335200
O -2.51995900 1.77661800 0.00001000
H -2.30442500 -0.74620600 -1.86586400
4_77
0 1
C 2.47155700 -0.15147800 -0.01186000
C 0.60380300 0.94535300 0.11908700
C 0.00933800 -1.47035500 -0.04454900
P 1.02074600 -0.36131500 -1.20282400
P -0.47995500 -0.12274200 1.25676600
P 0.16745300 2.72890200 -0.13042900

H	0.96639800	2.91526300	-1.29468000
H	-1.01788100	2.45724100	-0.86670900
O	1.92361200	0.85129500	0.73809300
O	0.52820800	-2.69728200	0.14947600
O	3.54596100	-0.61905900	0.13168300
H	-0.05053200	-3.20374600	0.73087500
C	-1.88424100	-0.46192100	0.10766500
O	-1.33896200	-1.51197200	-0.57422200
P	-3.41304000	0.22747100	-0.05695300
H	-3.79933800	-0.60695100	-1.15019200

4_78
0 1

C	2.39075500	-0.59103100	0.07810900
C	0.40659700	-1.44347700	-0.07416800
C	0.06724500	1.02089600	0.11659400
P	0.97630000	-0.18374200	1.25997200
P	-0.54176500	-0.24389800	-1.19565000
O	1.74026300	-1.55311100	-0.64496700
O	3.50693600	-0.23792300	-0.08448700
C	-1.91387500	0.18093700	-0.03835600
O	-1.26201000	1.12316000	0.70144000
P	-3.51199500	-0.33985900	0.06450000
H	-3.82153000	0.49054000	1.18552100
P	0.87238500	2.62063500	-0.32170800
H	-0.28789200	3.18111400	-0.92407700
H	0.63973000	3.23723900	0.93955300
O	-0.17816000	-2.64491300	0.09783300
H	0.38526000	-3.20757900	0.64067900

4_79
0 1

C	2.15480900	-0.90457400	-0.06005600
C	0.83835100	0.81351300	0.10674100
C	-0.62580400	-1.25673500	0.07287500
P	0.65691300	-0.58880100	-1.17121200
P	-0.54107500	0.24760200	1.26218300
P	1.10297800	2.62483600	-0.18860500
H	1.86770200	2.46377200	-1.37962500
H	-0.12249100	2.82437400	-0.87991300
O	2.05920900	0.24491700	0.67150100
O	-0.32544800	-2.54550700	0.46870900
O	2.98809000	-1.73223500	0.04935500
H	-0.97276100	-2.84026500	1.11621900
P	-2.36128400	-0.92919200	-0.65246200
H	-3.02460400	-1.36577500	0.53947200
C	-2.02204700	0.75200800	0.19302200
O	-2.55718700	1.80481900	0.05790900

4_80
0 1

C	0.47314100	1.61285200	-0.88117000
C	1.24620300	-0.18886900	0.05396800
C	-1.06847100	0.16539200	1.07489000
P	0.48836300	1.27764100	0.98104600
P	-0.11507900	-1.40414400	0.57391300
P	2.98104100	-0.83764300	0.13627200
H	3.62119500	0.42795800	0.27601900
H	2.95618900	-1.09414500	1.53492600
O	1.06330700	0.43924300	-1.25210700
O	-1.77572900	0.24021500	2.26952300
O	0.12490100	2.47913400	-1.60422200
C	-1.23249800	-1.25122700	-0.96601400
P	-2.28370600	0.22402500	-0.37964300
H	-3.20058600	-0.66440600	0.25542900
O	-1.25990400	-1.88001000	-1.96831000
H	-1.16693600	0.17486000	3.01068100

4_81
0 1

C	-0.60303400	0.51177100	1.74181200
C	-1.16677400	-0.41080700	-0.13847400

C	0.56499000	1.31751500	-0.71028900
P	-1.12838100	1.44955600	0.18384700
P	0.25001100	-0.43725300	-1.44838300
P	-2.62548200	-1.46429300	-0.58864200
H	-3.60250100	-0.72411000	0.13787100
H	-2.88750300	-0.79552900	-1.81668100
O	-0.76358400	-0.73757700	1.21703000
O	1.00652200	2.41599900	-1.37954400
O	-0.26512200	0.77433400	2.83996300
C	1.64766400	-0.47943900	-0.26021700
P	2.65305000	-1.71679300	0.26947600
H	3.37304300	-0.89061600	1.18562400
O	1.64302900	0.84549300	0.09146900
H	0.25512300	2.92177600	-1.69963000

4_82

0 1

C	-3.08303700	1.22522300	0.17560500
C	0.64502600	-0.29551300	0.38638500
C	2.54786900	0.69089400	-0.60202700
C	-1.51652700	-0.74019600	-0.51224800
P	-3.27920000	-0.19111600	-0.73047700
P	1.60401400	-0.58944300	-1.22359100
P	1.57625500	1.28532200	0.81895000
P	0.73160800	-1.62163600	1.71448000
H	0.50202900	-2.74101100	0.86935200
H	2.14625900	-1.75304400	1.67209800
O	-0.74301300	0.08826000	0.21048700
O	-1.13668800	-1.76680700	-1.00138600
O	3.68633400	1.26343500	-0.97700200
O	-3.07180800	2.20705200	0.76526200
H	2.39978500	0.78934600	1.87841600
H	4.02319800	0.83982000	-1.77549300

4_83

0 1

C	1.19773900	-0.42616900	1.92304700
C	-0.44766300	1.75853700	-0.12323000
C	-1.47493700	-0.40552800	-0.23990400
C	1.28322200	-0.32738400	-0.71679100
P	2.28488800	-0.99625900	0.77257900
P	1.11886700	1.53359400	-0.77797100
P	-0.25226300	-1.36952800	-0.88535600
P	-3.11952200	-1.18908200	-0.07052400
H	-3.30402400	-0.88096400	1.30830900
H	-3.87971300	-0.06778600	-0.51291800
O	-1.42469100	0.89327700	0.20037800
O	-0.83990000	3.00254400	0.15276800
O	0.49294900	-0.06613100	2.75796100
O	2.18216100	-0.70453300	-1.75783300
H	-1.79007200	2.99224600	0.31999100
H	1.85994000	-0.32235400	-2.58122600

4_84

0 1

C	-1.73402600	-0.35093600	0.05479500
C	1.54442500	0.10882100	-1.16900000
C	2.02899700	-1.06041100	1.19297400
C	-0.69084800	1.43899000	0.16149600
P	-1.27293000	0.29693600	1.56941200
P	1.80768400	-1.59091800	-0.39211800
O	-1.28800400	2.66900400	0.17720900
H	-1.04770300	3.14877400	-0.62432500
O	2.22777100	-0.83099600	2.30061700
P	-2.42032700	-1.90397700	-0.59869800
H	-3.12590100	-2.23651000	0.58730300
H	-3.53724100	-1.30505900	-1.24626200
O	-1.29323300	0.55313300	-0.84997100
O	1.66886300	0.18545000	-2.35696500
P	1.17124600	1.63124800	-0.11608800
H	1.01127200	2.46196000	-1.26305700

4_85
0 1

C	1.82998100	-1.03172100	0.63006100
C	0.89957900	0.59340600	-0.23610000
C	-1.32238400	-1.26189200	-0.65023300
P	2.23752600	-0.55275800	-0.96970500
P	-0.72258500	0.47943400	-1.12411500
P	1.29020900	2.36980800	0.22286500
H	2.66899400	2.12114900	0.48034100
H	1.53124700	2.78217900	-1.11703300
O	0.88318700	-0.17402300	1.03568000
O	-0.98967600	-2.32752200	-1.05580700
O	2.22327800	-1.95684500	1.47262600
C	-1.99696300	0.92678900	0.21004400
P	-2.60231800	-0.81410200	0.67508600
H	-3.76008700	-0.84696200	-0.15227400
O	-2.32457100	1.98675900	0.63653600
H	1.71835500	-1.87854400	2.29208600

4_86
0 1

C	-1.47563900	-1.13830100	-0.96286200
C	-0.91291900	0.72634300	-0.02240700
C	-0.08431300	-1.14634200	1.35067200
P	-1.88684200	-0.67083900	0.82993600
P	0.62486300	0.56384100	1.04285900
P	-1.48867700	2.46168100	-0.35056100
H	-2.83025800	2.12984100	-0.68973500
H	-1.79040400	2.75528500	1.00802000
O	-0.83695800	0.01591000	-1.29026000
O	0.35547600	-2.16240300	1.76378800
O	-1.67063500	-2.06363600	-1.66717100
C	2.10570300	0.44846700	-0.02799300
P	2.34313500	-0.88518500	-1.04159200
H	3.61666000	-0.44335700	-1.51789300
O	2.97991500	1.47313700	0.09043200
H	2.69743200	2.07070200	0.79121500

4_87
0 1

C	-0.41807100	-1.23019900	0.19403700
C	2.24714300	-0.24689700	-0.23898100
C	0.39851600	1.19310500	-0.13139000
C	-2.22964100	0.26381900	0.15011300
P	-0.70703200	0.36271600	1.16033700
P	-2.17888700	-1.31384900	-0.49476400
O	-3.14327800	1.21024400	-0.03146000
O	3.14019600	-1.22932400	-0.21437100
O	0.11355600	-2.29526000	0.90259500
H	0.02016400	-3.09306200	0.37473400
P	0.67939200	-0.39521100	-1.15782400
P	2.17019300	1.29368600	0.50319400
H	-2.78803200	2.05788700	0.25775100
H	3.87649900	-0.99451900	0.36208200
O	-0.22383800	2.33718600	-0.62380700
H	0.35560700	2.75783100	-1.26503300

4_88
0 1

C	1.20050600	-0.02602000	-0.06213800
C	-0.34303500	1.47795700	0.04421100
C	-0.91167900	-0.74793300	1.51786300
C	-1.04496900	-0.69248100	-0.98651800
P	0.12575700	-1.62765100	0.19442900
P	-1.90173200	0.36825000	0.34660600
P	0.21201800	0.58869200	-1.56770300
O	-1.75862500	-1.32990000	-1.98565700
O	0.81722500	1.08298400	0.75987600
P	3.01467100	-0.32066300	0.12281200
H	3.09140000	-1.29413600	-0.90976300
H	3.45022500	0.74840400	-0.71382400

O	-0.63040300	2.78734600	0.22508000
H	0.17543000	3.29654700	0.07784800
H	-2.39005500	-1.93551700	-1.58776500
O	-0.86477800	-0.79216400	2.70205500

4_89
0 1

C	-0.62012700	-1.25788000	0.06541500
C	2.16204900	-0.88894000	-0.05462500
C	0.84465300	0.82265800	0.11531300
C	-2.03576200	0.74173300	0.19348300
P	-0.54383300	0.22716700	1.24579800
P	-2.35456100	-0.93536200	-0.66616800
O	-2.59465500	1.78412900	0.09711400
O	2.98104700	-1.73243800	0.06012600
H	-3.02062300	-1.39159500	0.51244100
O	-0.38003500	-2.49113900	0.64140900
H	-0.43755500	-3.18166000	-0.02582300
P	0.68346500	-0.56021400	-1.17971600
P	0.95414800	2.60470900	-0.34815300
H	1.47977500	3.03969300	0.90042900
H	2.23374700	2.48800000	-0.95923700
O	2.06257900	0.25140200	0.68613100

4_90
0 1

C	-1.56032300	-0.40111700	1.22918800
C	1.63187000	-0.56074400	-0.04348000
C	0.23506000	1.86673800	0.02135000
C	-1.62322100	-0.33066700	-1.21152100
P	-2.18826100	-1.70630300	-0.01448600
P	-1.42697200	1.06693500	0.04632500
P	1.84768100	1.29477900	-0.01091000
P	3.26490600	-1.46506900	0.09745000
H	2.92914700	-2.47242400	-0.84241100
H	3.97563900	-0.63796300	-0.81212300
O	0.59864400	-1.19050700	-0.05908300
O	-1.49210400	-0.33939400	-2.38866200
O	0.12406400	3.20727000	0.02943500
O	-1.37006300	-0.47712400	2.39577100
H	-3.53790600	-1.24157400	0.03106100
H	-0.81182400	3.44961400	0.05487300

4_91
0 1

C	1.00682800	1.62729400	0.57672400
C	-0.62454800	-0.82579600	0.14388300
C	-1.76032400	1.27388600	-0.46999500
C	1.63580500	-0.70253100	-0.73033100
P	2.44486000	0.57200700	0.13580500
P	-0.64499900	0.85203900	0.99260900
P	-2.28390800	-0.53645200	-0.73169700
P	-0.60671600	-2.39664900	1.17508600
H	0.55676400	-2.06751400	1.93073500
H	-1.49886700	-1.89627200	2.16312800
O	0.36790900	-1.02530300	-0.89076000
O	2.35705400	-1.54074500	-1.46443500
H	3.26491300	-1.21504200	-1.50520600
O	-2.05979300	2.28117200	-1.01539800
O	1.11973400	2.81656200	0.71669600
H	-1.78716000	-0.67596000	-2.05622300

4_92
0 1

C	1.97435100	-0.92563400	-0.28646400
C	-1.66874100	0.74497000	-0.17240700
C	2.49026000	1.50943200	0.39119900
C	-0.65728200	-1.48929400	-0.13626800
P	0.80018600	-2.40222000	-0.11960400
P	1.94403800	0.18347000	1.27826100
P	-1.08370500	2.30355000	-0.50107800
P	-3.39717200	0.22634400	0.23003100

H	-3.15720000	-0.27293400	1.53934400
H	-3.77236100	1.51869600	0.67779900
O	-0.68446100	-0.19635400	-0.41683600
O	-1.86682200	-2.02365700	0.03325000
H	-1.78187900	-2.94569200	0.30026100
O	2.86174900	2.41122900	-0.21209900
O	2.71880500	-0.81035600	-1.21030900
H	-2.30447000	2.94901800	-0.15996500

4. References

[1] D. Heift, Z. Benkő, H. Grützmacher, *Dalton Trans.* **2014**, *43*, 831–840.

[2] G. R. Fulmer, A. J. M. Miller, N. H. Sherden, H. E. Gottlieb, A. Nudelman, B. M. Stoltz, J. E. Bercaw, K. I. Goldberg, *Organometallics* **2010**, *29*, 2176–2179.

[3] M. J. Frisch, G. W. Trucks, H. B. Schlegel, G. E. Scuseria, M. A. Robb, J. R. Cheeseman, G. Scalmani, V. Barone, B. Mennucci, G. A. Petersson, H. Nakatsuji, M. Caricato, X. Li, H. P. Hratchian, A. F. Izmaylov, J. Bloino, G. Zheng, J. L. Sonnenberg, M. Hada, M. Ehara, K. Toyota, R. Fukuda, J. Hasegawa, M. Ishida, T. Nakajima, Y. Honda, O. Kitao, H. Nakai, T. Vreven, J. A. Montgomery, J. E. Peralta, F. Ogliaro, M. Bearpark, J. J. Heyd, E. Brothers, K. N. Kudin, V. N. Staroverov, R. Kobayashi, J. Normand, K. Raghavachari, A. Rendell, J. C. Burant, S. S. Iyengar, J. Tomasi, M. Cossi, N. Rega, J. M. Millam, M. Klene, J. E. Knox, J. B. Cross, V. Bakken, C. Adamo, J. Jaramillo, R. Gomperts, R. E. Stratmann, O. Yazyev, A. J. Austin, R. Cammi, C. Pomelli, J. W. Ochterski, R. L. Martin, K. Morokuma, V. G. Zakrzewski, G. A. Voth, P. Salvador, J. J. Dannenberg, S. Dapprich, A. D. Daniels, Ö. Farkas, J. B. Foresman, J. V. Ortiz, J. Cioslowski, D. J. Fox, *Gaussian 09 Rev. D.01*, Gaussian Inc., Wallingford CT, **2009**.

[4] E. D. Glendening, C. R. Landis, F. Weinhold, *J. Comput. Chem.* **2013**, *34*, 1429–1437.

[5] E. D. Glendening, F. Weinhold, *J. Comput. Chem.* **1998**, *19*, 593–609.

[6] E. D. Glendening, F. Weinhold, *J. Comput. Chem.* **1998**, *19*, 610–627.
